# Sidechain Diversification of Grandifloracin Allows Identification of Analogues with Enhanced Anti‐Austerity Activity against Human PANC‐1 Pancreatic Cancer Cells

**DOI:** 10.1002/cmdc.201900549

**Published:** 2019-12-10

**Authors:** Benjamin E. Alexander, Sijia Sun, Matthew J. Palframan, Gabriele Kociok‐Köhn, Dya Fita Dibwe, Shiro Watanabe, Lorenzo Caggiano, Suresh Awale, Simon E. Lewis

**Affiliations:** ^1^ Department of Chemistry University of Bath Bath BA2 7AY UK; ^2^ Institute of Natural Medicine University of Toyama 2630 Sugitani Toyama 930-0194 Japan; ^3^ Materials and Chemical Characterisation Facility (MC^2^) University of Bath Bath BA2 7AY UK; ^4^ Department of Pharmacy and Pharmacology University of Bath Bath BA2 7AY UK

**Keywords:** cancer, nutrient deprivation, antiproliferation, dearomatization, dimerization

## Abstract

The natural product (+)‐grandifloracin is a potent “anti‐austerity” agent, able to suppress the ability of various pancreatic cancer cell lines to tolerate conditions of nutrient deprivation. Such anti‐austerity agents represent a promising approach to cancer chemotherapy. Here we report the synthesis and biological evaluation of racemic analogues of grandifloracin bearing diverse sidechains, of which two show enhanced potency in comparison with the natural product. Additionally, several unexpected by‐products containing modifications of the grandifloracin core were isolated, identified and similarly evaluated for biological activity.

## Graphical abstract

**Austerity measures**: Grandifloracin is a natural product that is known to exhibit anti‐austerity activity, *i. e*. it is able to abolish the ability of pancreatic cancer cells to survive under “austere” conditions of nutrient deprivation. Herein we report the synthesis and biological evaluation of analogues of grandifloracin, as well as our findings on rearrangement and fragmentation reactions of the dimeric grandifloracin core. Live imaging, fluorescence microscopy and colony formation experiments with the most active analogue are described.

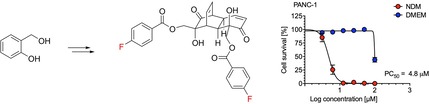

## Introduction

Pancreatic cancer is one of the most aggressive human malignancies, and as it is often initially asymptomatic, most patients already have metastases upon presentation. Therefore, surgical resection is usually not appropriate.^1^ Pancreatic tumours show intrinsic resistance to chemotherapeutic agents that are effective against other tumour types.^2^ There are no truly effective drugs for pancreatic cancer – It has a median survival of less than 6 months and the lowest 5‐year survival rate (5.5 %) of all cancers. For example, ≈23,000 people in Japan^3^ and ≈10,000 in the UK^4^ die of pancreatic cancer each year. Across the EU, pancreatic cancer has now surpassed breast cancer to become the third leading cause of cancer related death. Although use of gemcitabine as a single agent therapy has been superseded by combination therapies as the first line therapies of choice, even these impart only modest survival benefits.^5^ It is clear a major breakthrough will require the use of entirely new therapeutic strategies exploiting emerging targets.^6^

**Figure 1:**
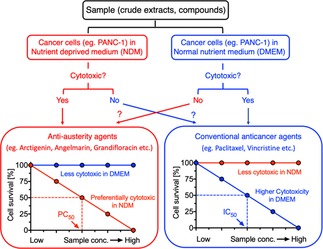
The anti‐austerity strategy in anticancer drug discovery.

The “anti‐austerity” therapeutic strategy was first described by the Esumi group at the Japan National Cancer Center Research Institute in 2000.^7^ It targets the ability of certain cancer cell lines to survive severe nutrient deprivation. Pancreatic tumours are inherently hypovascular in nature, leading to limited supply of essential nutrients to rapidly growing tumour cells.^7^ Therefore these cells are under constant metabolic stress, which may contribute to genomic instability, impaired cellular repair, mutagenesis and chemoresistance. In order to survive under such extreme conditions, the cancer cells alter their metabolism and acquire tolerance of nutrient starvation. Pancreatic cancer cell lines, in contrast to other cell lines, demonstrate an extraordinary capacity for survival in conditions of severe nutrient deprivation. Thus, discovery of agents that eliminate the cancer cells’ tolerance of nutrient starvation (anti‐austerity agents, Figure 1) is a novel strategy in anticancer drug discovery.^8^, ^9^

Elucidation of how pancreatic cancer cells can survive and proliferate in such “austere” conditions is ongoing; numerous proteins and pathways have been implicated.^10^ Nevertheless, Esumi has described an assay to identify anti‐austerity agents without knowledge of their specific target.^11^ Briefly, two cell cultures are grown in different media: one in normal medium (DMEM, Dulbecco's modified Eagle's medium) and one in nutrient‐deprived medium (NDM). The cultures are treated with serial dilutions of the agent being evaluated and incubated for 24 h, and then cell survival is determined. Compounds which are cytotoxic in NDM, without cytotoxicity in DMEM are deemed anti‐austerity agents. Quantitative data are expressed as PC_50_ values, denoting preferential cytotoxicity in NDM. By this approach, several dozen anti‐austerity agents have been identified.^10^, ^12^, ^13^, ^14^, ^15^, ^16^, ^17^, ^18^, ^19^, ^20^, ^21^, ^22^, ^23^, ^24^ In several cases efficacy has been demonstrated *in vivo* (xenograft PANC‐1 tumours in nude mice).^8^, ^9^, ^25^

The natural product arctigenin has been identified as a potent anti‐austerity agent, and has commenced clinical evaluation in the form of “GBS‐01”, an extract of the fruit of *Arctium lappa* Linné (rich in arctigenin), which is registered in the Japanese Pharmacopoeia as a traditional herbal medicine. A successful phase I trial has established favourable clinical responses and toxicity profile in patients with advanced pancreatic cancer refractory to gemcitabine.^26^ A Phase IIa trial to evaluate efficacy and safety has been completed, and a patent was recently granted on use of arctigenin in combination therapy with gemcitabine.^27^ Thus, although no chemotherapeutic agent with known anti‐austerity activity has yet received approval, the approach is exceedingly promising.

**Figure 2:**
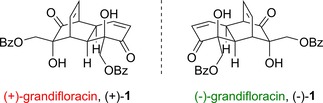
The two enantiomers of grandifloracin **1**.

Grandifloracin is a dimeric natural product which is notable for having been isolated from Nature in both enantiomeric forms. The first reported isolation of (−)‐grandifloracin, (−)‐**1** was in 1997, from the tropical flowering plant *Uvaria grandiflora*,^28^, ^29^ followed by later isolations from *Uvaria rufa* 30 and *Uvaria calamistrata*.^31^ Although the relative stereochemistry of (−)‐**1** was established, the absolute configuration remained unknown until 2011. As part of our ongoing interest in polyoxygenated cyclohexene natural products,^32^ we (S.E.L. and co‐workers) carried out a total synthesis of grandifloracin^33^ employing a starting material of known absolute configuration.^34^ Our synthetic grandifloracin had a positive optical rotation, from which we could therefore infer the absolute configuration of (−)‐grandifloracin, (−)‐**1**, as shown in Figure 2.

At that point, (+)‐grandifloracin had not been isolated from a natural source, so we described the synthetic (+)‐**1** as the “non‐natural enantiomer of grandifloracin”.^33^ However, in 2012 we (S.A. and co‐workers) reported the isolation of (+)‐grandifloracin from *Uvaria dac* Pierre ex Finet & Gagnep,^35^ thereby showing that both enantiomers occur in Nature. The isolation from Nature of several other polyoxygenated cyclohexene natural products in both enantiomeric series has been reported for different species in several genera, or even from the same species. Care must therefore be taken when assigning absolute configuration of new natural products in this class, as it cannot be inferred from the absolute configuration of co‐isolates, nor from biosynthetic speculation. The possible biosynthetic origins of this enantiodivergence have previously been discussed.^32^ The stems of *Uvaria dac* were also found to contain several further novel polyoxygenated cyclohexenes.^35^, ^36^ All the isolates were evaluated in the anti‐austerity assay, with (+)‐**1** proving the most potent, exhibiting PC_50_=14.5 μM against PANC‐1 cells in nutrient‐deprived medium.

These results prompted us (S.A. and co‐workers) to undertake more extensive biological investigations of (+)‐**1**, which showed that it induces PANC‐1 cell death under conditions of nutrient deprivation through hyperactivation of autophagy.^37^ While suppression of autophagy is a more well‐established strategy in cancer chemotherapy,^38^ hyperactivation of autophagy is an alternative approach which has been clinically validated in other tumour types – for example, the autophagy inducer temozolomide has therapeutic benefits against apoptosis‐resistant cancers.^39^, ^40^ In the case of (+)‐**1**, we found no change in Bcl‐2 and caspase‐3 expression (apoptosis marker proteins), but instead observed pronounced upregulation of microtubule‐associated protein 1 light chain 3 (LC3), an autophagy marker. This was dose‐dependent and time‐dependent following administration of (+)‐**1**. Furthermore, LC3 activation mediated by (+)‐**1** was marginal in DMEM, but vastly more pronounced in NDM. In addition to the above, it was found that (+)‐**1** strongly inhibited the activation of Akt. Specifically, we observed complete inhibition of Akt phosphorylation at Ser473 upon treatment with (+)‐**1**. Complete inhibition of mTOR phosphorylation at Ser2448 was similarly observed (mTOR is a downstream effector of Akt). The serine/threonine kinase Akt/mTOR pathway is constitutively activated in most pancreatic cancer cell lines and is associated with tolerance of nutrient deprivation,^41^ tumour progression and metastasis.^42^ Three previously identified anti‐austerity agents have been shown to suppress Akt activation^43^, ^44^, ^45^ and it is believed their activity derives at least in part from this. However, our study explicitly shows (+)‐**1** to be a dual inhibitor of both Akt *and* mTOR, both clinically relevant prosurvival factors.

**Figure 3:**
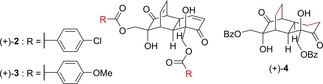
Analogues of (+)‐**1** previously reported by us.

Motivated by these findings, we (S.E.L., L.C. and co‐workers) undertook an initial analogue study on (+)‐**1**.^46^ We prepared three analogues of (+)‐**1** via modifications of our previous synthesis,^33^ all of which were slightly more active than (+)‐**1** (Figure 3). Two of these, (+)‐**2** and (+)‐**3**, were variants bearing different ester sidechains, whereas the third, (+)‐**4**, was the product of hydrogenation of (+)‐**1**. All three of these showed slightly enhanced potency with respect to (+)‐**1**, and the retention of activity in the saturated analogue (+)‐**4** indicated that the enone motif is not required for anti‐austerity activity. From this we concluded that covalent modification through Michael addition is not the mechanism of action by which (+)‐**1** interacts with its target(s). Encouraged by these findings, we embarked on a programme of synthesis of further analogues of (+)‐**1**, the results of which are reported here.

**Scheme 1:**
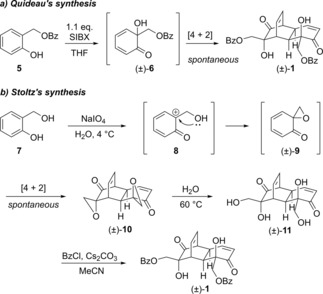
Reported syntheses of (±)‐**1**.

Our previous syntheses of grandifloracin and analogues employed a single‐enantiomer starting material and hence gave the final products as single enantiomers also. While ours remains the only synthesis of enantiopure grandifloracin (+)‐**1**, two syntheses of *racemic* grandifloracin, (±)‐**1** have been reported. In 2007, Quideau *et al*. published a synthesis of (±)‐**1** that employed an oxidative dearomatisation of an achiral starting material, mediated by SIBX, a stabilised formulation of IBX (λ^5^‐iodane 2‐iodoxybenzoic acid).^47^ Thus, salicyl benzoate **5** undergoes oxidation to form cyclohexadienone **6**, which in turn spontaneously dimerises by a Diels‐Alder reaction to afford (±)‐**1** (Scheme 1a). In 2015 Stoltz and co‐workers reported a total synthesis of (±)‐**1** whereby the oxidative dearomatisation/dimerisation sequence occurs prior to introduction of the ester sidechains.^48^ Thus, salicyl alcohol undergoes oxidation by sodium periodate to afford intermediate **8**. The primary alcohol then effects an intramolecular nucleophilic attack to give spirocyclic epoxide (±)‐**9**. Here also a spontaneous Diels‐Alder dimerisation occurs to give bis(epoxide) (±)‐**10**. This reaction sequence was first reported by Adler,^49^ and variants thereof have been employed to access diverse synthetic targets.^50^, ^51^ Hydrolytic opening of the epoxides in (±)‐**10** gave previously unknown tetraol (±)‐**11**, which underwent selective benzoylation at the primary alcohols in preference to the tertiary alcohols, giving (±)‐**1** (Scheme 1b).

Both these racemic syntheses (as well as our earlier synthesis) rely on the Diels–Alder dimerization as the key complexity‐forming event in what are overall very concise synthetic routes. This cycloaddition is remarkably regio‐ and stereoselective and the origins of this selectivity have been studied.^52^ The Stoltz route in particular is appropriate for analogue synthesis, since sidechain diversity may be introduced in the final step, by derivatisation of common building block (±)‐**11**. Therefore, for the present study we opted to adopt the Stoltz approach. Whilst this synthetic route produces racemic analogues, any that were found to be of particular interest could then be subject to resolution and biological evaluation of the individual enantiomers; conditions for preparative chiral HPLC resolution of (±)‐**1** have been reported.^48^

## Results and Discussion

### Synthesis

**Figure 4:**
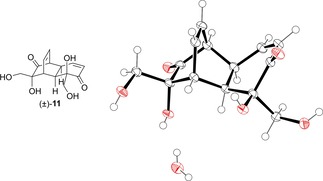
Solid‐state structure of (±)‐**11**⋅H_2_O, crystallised from ethanol/water. Ellipsoids are represented at 50 % probability. H atoms are shown as spheres of arbitrary radius. CCDC #1952565.

**Scheme 2:**
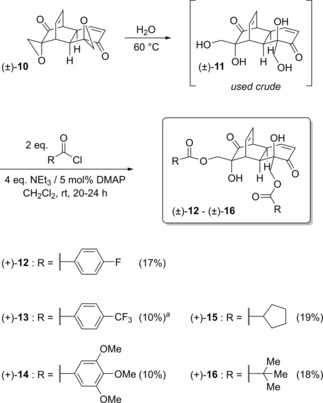
Telescoped synthesis of grandifloracin ester analogues from (±)‐**10**. Yields shown are isolated yield over two steps. ^*a*^4 equivalents of pyridine used as base/catalyst.

Tetraol (±)‐**11** was synthesised and purified by chromatography on silica, as per the reported procedure in Scheme 1b. From this purified material we were able to grow crystals of (±)‐**11** as its monohydrate, suitable for x‐ray diffraction, and the structure obtained is shown in Figure 4. In the solid state, (±)‐**11** is engaged in a complex network of intermolecular hydrogen bonding interactions (see ESI). For the purposes of synthesising new ester analogues of (±)‐**1**, we instead opted to take crude (±)‐**11** directly into the acylation step with the relevant acid chloride (Scheme 2).

As shown in Scheme 2, both aliphatic and aromatic ester analogues were synthesised. Unoptimised yields over two steps for these analogues were moderate, but this telescoped procedure rapidly afforded pure material in sufficient quantity for biological evaluation.

**Scheme 3:**
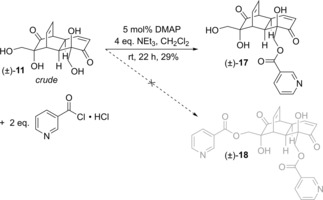
Unexpected synthesis of monoacyl product (±)‐**17**.

For analogues (±)‐**12** to (±)‐**16**, no under‐ or over‐acylated byproducts were isolated, implying a good selectivity for acylation of the two primary hydroxyl groups in (±)‐**11** over the two tertiary ones. In contrast, the use of nicotinoyl chloride as the acylating agent resulted in the isolation only of monoacyl product (±)‐**17** instead of the expected bis(nicotinoyl) ester (±)‐**18** (Scheme 3). The structure of (±)‐**17** was assigned based on 2D‐NMR data. Thus, the ester carbonyl carbon exhibits a ^3^ *J*_CH_ coupling to the adjacent methylene *AB* protons which is observable in the HMBC spectrum. These same methylene protons also exhibit a ^3^ *J*_CH_ coupling to a second carbonyl carbon, again observable in the HMBC spectrum. The chemical shift of this carbonyl (δ=198.1 ppm) identifies it as the conjugated ketone (and differentiates it from the non‐conjugated ketone carbonyl carbon at δ=204.2 ppm), thereby confirming the connectivity present in (±)‐**17** (see ESI for full details).

**Scheme 4:**
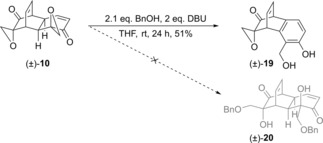
Unexpected synthesis of byproduct (±)‐**19**.

In addition to ester analogues (±)‐**12** to (±)‐**17**, we also sought to synthesise ether analogues of grandifloracin. We reasoned that ether sidechains would be much more hydrolytically stable than ester analogues, and might therefore exhibit appreciably different PC_50_ values. Our first attempts to access analogues of grandifloracin bearing ether sidechains involved treating bis(epoxide) (±)‐**10** with an oxygen nucleophile, in the hope of effecting epoxide ring opening. Such an approach would provide the desired analogues in a single step. Thus, as shown in Scheme 4, we treated (±)‐**10** with benzyl alcohol under basic conditions. However, this afforded unexpected product (±)‐**19**, with none of the bis(benzyl ether) (±)‐**20** being produced.

**Figure 5:**
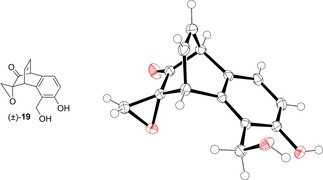
Solid‐state structure of (±)‐**19**, crystallised from ethyl acetate/petrol (40‐60 °C). Ellipsoids are represented at 50 % probability. H atoms are shown as spheres of arbitrary radius. CCDC #1952563.

No incorporation of a benzyl group took place in the formation of byproduct (±)‐**19**. Instead, aromatisation of the ring containing the enone occurred, with the remaining epoxide being unchanged. The same product formed when benzyl alcohol was omitted from the reaction. The structure of (±)‐**19** was confirmed by x‐ray crystallography (Figure 5).

**Scheme 5:**
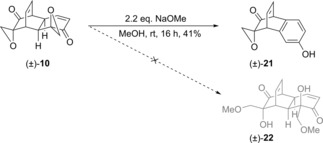
Unexpected synthesis of byproduct (±)‐**21**.

In an attempt to favour the desired reaction pathway, we employed a pre‐formed alkoxide in place of an alcohol,^53^ treating (±)‐**10** with sodium methoxide in methanol. Here again, however, aromatisation was favoured over incorporation of the nucleophile, with byproduct (±)‐**21** being formed instead of the desired bis(methoxy) analogue (±)‐**22** (Scheme 5).

**Scheme 6:**
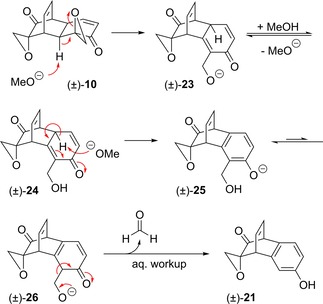
Proposed mechanism for formation of byproduct (±)‐**21**.

It is interesting to note that while the reactions shown in Schemes 4 and 5 both failed to provide the desired ether analogues, they did not form the same byproduct. Rather, (±)‐**21** is an analogue of (±)‐**19** from which a molecule of formaldehyde has formally been lost. A mechanistic proposal which would account for these outcomes is shown in Scheme 6. Exposure of bis(epoxide) (±)‐**10** to base leads to an elimination and concomitant epoxide opening to give cyclohexadienone (±)‐**23**. The alkoxide in (±)‐**23** is reprotonated to give (±)‐**24**, which can then undergo aromatisation *via* base‐mediated elimination of the doubly allylic proton to give phenoxide (±)‐**25**. This phenoxide would be in equilibrium with cyclohexadienone tautomer (±)‐**26**, from which a retro‐aldol process would lead to extrusion of formaldehyde and formation of the observed byproduct (±)‐**21**.

In the case of byproduct (±)‐**19**, the mechanism shown in Scheme 6 may proceed only as far as intermediate (±)‐**25**, before protonation in the workup to afford (±)‐**19**. The selectivity between the two sets of reaction conditions is seemingly high, with no (±)‐**19** being isolated in the synthesis of (±)‐**21** and *vice versa*. The different solvents employed in the reactions to form (±)‐**19** and (±)‐**21** may influence the reaction outcome by altering the position of the tautomeric equilibrium between (±)‐**25** and (±)‐**26**. Thus, for retro‐aldol product (±)‐**21** to form at an appreciable rate, tautomer (±)‐**26** must be sufficiently populated. The hydrogen bond donor properties of methanol might stabilise tautomer (±)‐**26** sufficiently for this to be the case, whereas in THF if the concentration of (±)‐**26** is negligible, (±)‐**19** will be formed in preference. Base strength may also play a role, as (±)‐**25** and (±)‐**26** will also be in equilibrium with their (neutral) conjugate acids. Since the *p*Ka of protonated DBU is lower than that of methanol,^54^, ^55^ (±)‐**26** will be more extensively protonated under the DBU/THF reaction conditions, and the conjugate acid of (±)‐**26** may undergo the retro‐aldol process less readily. It is interesting to note that Stoltz *et al*. reported isolating a byproduct with a structure closely analogous to (±)‐**19** (specifically, with no loss of formaldehyde) when performing acylations of tetraol (±)‐**11** in acetonitrile using cesium carbonate as base.

**Scheme 7:**
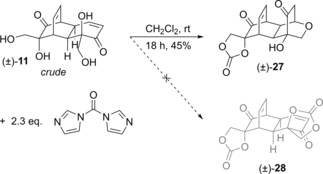
Unexpected synthesis of oxa‐Michael product (±)‐**27**.

As we were unable to access grandifloracin analogues through the direct opening of epoxides with nucleophiles, we next explored the possibility of functional group interconversion to replace the epoxides with alternative electrophilic groups. Specifically, we attempted to derivatise the vicinal diol motifs in tetraol (±)‐**11** as cyclic carbonates, which might then undergo nucleophilic substitution at the methylene carbons, giving the desired analogues with loss of carbon dioxide. However, treatment of (±)‐**11** with carbonyldiimidazole^56^, ^57^ led to unexpected byproduct (±)‐**27** instead of the desired bis(carbonate) (±)‐**28** (Scheme 7).

**Figure 6:**
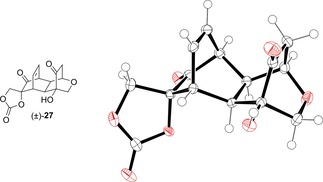
Solid‐state structure of (±)‐**27**, crystallised from MeOH/CH_2_Cl_2_. Ellipsoids are represented at 50 % probability. H atoms are shown as spheres of arbitrary radius. CCDC #1952564.

Formation of (±)‐**27** seemingly occurs by one vicinal diol forming the cyclic carbonate as expected, and the primary alcohol of the other vicinal diol participating in an intramolecular oxa‐Michael reaction with the enone. Surprisingly, attempts to induce this oxa‐Michael reaction by treating (±)‐**11** with other reagents such as imidazole itself were unsuccessful. This may imply that prior formation of the cyclic carbonate from the other diol is necessary to induce a conformational change that favours the oxa‐Michael process. The structure of (±)‐**27** was confirmed by x‐ray crystallography (Figure 6).

**Scheme 8:**
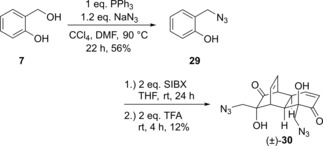
Synthesis of bis(azido) analogue (±)‐**30**.

Finally, we briefly explored the synthesis of nitrogen‐containing analogues of grandifloracin. Since we had been unable to effect nucleophilic addition of nucleophiles to bis(epoxide) (±)‐**10** or a cyclic carbonate derivative, we adopted a different synthetic approach, based on Quideau's route (Scheme 1a), incorporating nitrogen into the salicyl substrate for oxidative dearomatisation / dimerization. Thus, as shown in Scheme 8, salicyl alcohol underwent a benzylic substitution to give known azide **29**.^58^ This was treated with SIBX to give bis(azido) analogue (±)‐**30**.

## Biological Evaluation

**Figure 7:**
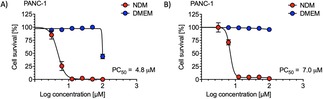
Preferential cytotoxic activity of A) (±)‐**12** and B) (±)‐**13** against the PANC‐1 human pancreatic cancer cell line in nutrient‐deprived medium (NDM) and Dulbecco's modified Eagle's medium (DMEM).

Grandifloracin analogues (±)‐**12** to (±)‐**17** and (±)‐**30**, as well as byproducts (±)‐**19**, (±)‐**21** and (±)‐**27**, were evaluated in the anti‐austerity assay against PANC‐1 cells as previously described. (Figure 7, Table 1). Their activity is represented by a PC_50_ value, the concentration at which 50 % of cancer cells were killed preferentially in nutrient‐deprived medium (NDM) without apparent toxicity in the normal nutrient‐rich medium (DMEM). Among the tested compounds, (±)‐**12** and (±)‐**13** displayed the most potent activity with PC_50_ values of 4.8 μM and 7.0 μM respectively, more potent than the natural product (+)‐grandifloracin (**1**, PC_50_ 14.5 μM). Interestingly, introducing a *p*‐fluoro or *p*‐trifluoromethyl‐substituent onto the phenyl groups increased the activity by 3‐fold and 2‐fold respectively. (**12**>**13**>**1**). However, the introduction of a methoxy substituent decreases the activity. Replacement of the phenyl groups with other ring systems or isobutyl groups led to dramatic loss of antiausterity activity. Byproducts (±)‐**19** and (±)‐**21** were weakly active in both nutrient‐deprived and normal media, likely due to their epoxides acting as electrophiles for non‐selective alkylation.^59^ This evidence suggests that the grandifloracin core is essential for the antiausterity activity, and that (±)‐**12** is an ideal candidate for further investigation of biological activity.

**Table 1:** Measured PC_50_ values for compounds tested. ^*a*^ See reference 35.

Compound	PC_50_/μM
(+)‐**1**	14.5^*a*^
(±)‐**12**	4.8
(±)‐**13**	7.0
(±)‐**14**	27.4
(±)‐**15**	>100
(±)‐**16**	51.8
(±)‐**17**	>100
(±)‐**19**	96.6
(±)‐**21**	36.6
(±)‐**27**	>100
(±)‐**30**	>100

**Figure 8:**
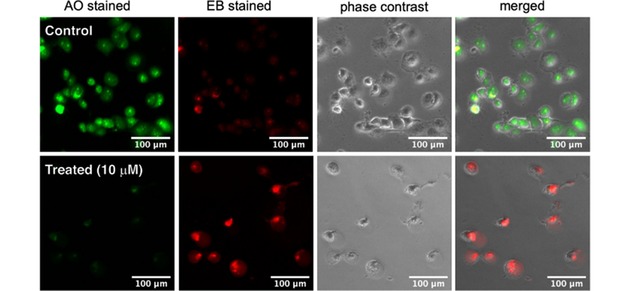
Morphological changes of PANC‐1 cells induced by (±)‐**12** (bottom row), in comparison to untreated cells (i. e., the control, top row) in nutrient‐deprived medium (NDM). PANC‐1 tumour cells were treated with 10 μM of (±)‐**12** in NDM and incubated for 24 h. Cells were stained with ethidium bromide (EB) and acridine orange (AO) and photographed under fluorescence (red and green) and phase contrast modes using an EVOS FL digital microscope.

PANC‐1 cells, even when exposed to complete nutrient deprivation for 24 hours, remain alive and show intact cellular morphology. On the other hand, treatment with (±)‐**12** dramatically alters the cancer cells’ morphology, resulting in the swelling and rounding of cells and leakage of the cytoplasmic contents into the media. Such difference of live and dying cells can be visualized by the ethidium bromide (EB) – acridine orange (AO) double‐staining fluorescence assay. AO is a cell‐membrane permeable dye and emits bright‐green fluorescence in living cells, while EB is only permeable through the membrane of dead or dying cells and emits predominantly red fluorescence. As shown in Figure 8, PANC‐1 cells treated with (±)‐**12** (10 μM) and the untreated control in NDM were incubated for 24 h, and then stained with the EB/AO reagent. The PANC‐1 cells in the control group showed intact morphology and emitted bright green fluorescence, suggesting 100 % cell survival even in the conditions complete nutrition starvation. However, PANC‐1 cells, when treated with (±)‐**12** emitted predominantly red fluorescence due to EB, with rounded morphology characteristic of dead cells.

**Figure 9:**
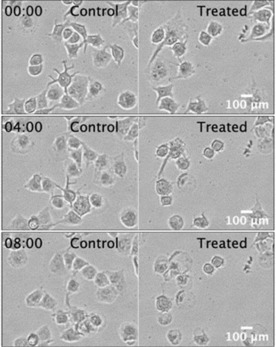
Captures of the live imaging of the effect of (±)‐**12** (10 μM) on PANC‐1 cells at different intervals of time (hour: minute)

The effect of (±)‐**12** against PANC‐1 cells in NDM was further investigated in a parallel real‐time time‐lapse microscopy experiment. The control (NDM only) and treated (25 μM of (±)‐**12** in NDM) PANC‐1 cells were incubated within a CO_2_ incubator equipped with real‐time microscopy system. The images were captured every 15 min on the digital cell imaging system for 24 h. Treatment of PANC‐1 cells with (±)‐**12** inhibited cell mobility within 30 minutes and induced cell death within 4 h, leading to cell death after 8 h (Figure 9, Movie 1). PANC‐1 cells cultured under the same conditions in NDM (control) continued to survive until the end of the experiment (24 h). Thus, this study further demonstrated the first live evidence of the anticancer therapeutic potential of (±)‐**12**.

**Figure 10:**
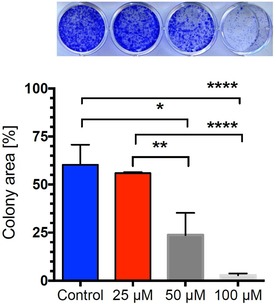
Effect of (±)‐**12** on colony formation by PANC‐1 cells. (A) PANC‐1 cell colonies treated with different concentrations of (±)‐**12**. (B) Graph showing mean values of the area occupied by PANC‐1 cell colonies (three replicates). ****p<0.0001, *p<0.05 when compared with the untreated control group.

Pancreatic cancer is known for its high metastatic potential. Most patients at the point of diagnosis show colonies of migrated pancreatic tumours in the duodenum, liver, and other organs where the tumour cells have sufficient nutrition to grow into large tumours. Compounds having the ability to inhibit such colony formation have therapeutic benefit against cancer ‘colonization’. Therefore, the effect of (±)‐**12** was investigated for its effect against PANC‐1 cell colony formation in normal nutrient‐rich medium. Compound (±)‐**12** was exposed to PANC‐1 cells at the concentration of 25, 50 and 100 μM in nutrient‐rich DMEM medium for 24 hours. The medium was then replaced by fresh DMEM and incubated for a further 10 days to allow colony formation. As shown in Figure 10, (±)‐**12** inhibited the colony formation ability of PANC‐1 cells, even when exposed for only 24 hours at the non‐cytotoxic concentration. The average colony area of each well within 12 well plates was found to be 60 % for the control, which was significantly decreased to 55 %, 23 % and 3 % for those treated with (±)‐**12** at 25, 50 and 100 μM concentrations. (See ESI for statistical analysis).

## Conclusions

We have prepared several analogues of grandifloracin and determined their PC_50_ values in the anti‐austerity assay. Of the compounds tested, (±)‐**12** exhibited the best PC_50_ value and hence was subjected to additional biological evaluation. The fluorescence microscopy, live imaging and colony formation experiments all provide additional evidence of the anti‐austerity activity of (±)‐**12**. The measured PC_50_ value for (±)‐**12** indicates a 3‐fold increase in potency compared to (+)‐**1**. However, it should be noted that (±)‐**12** was prepared and evaluated as a racemate. If (±)‐**12** were resolved, the single enantiomer (+)‐**12** would be expected to show a further increase in potency. Taken as a whole, these results are strongly encouraging for drug discovery activities related to the grandifloracin core; further efforts in this regard are ongoing in our laboratories and will be reported in due course.

## Experimental Section

### General Conditions

All reactions were carried out under an atmosphere of N_2_ or argon, through the use of a Schlenk line, unless otherwise stated. Anhydrous solvents were either purchased from Fisher Scientific or Sigma‐Aldrich, or purified through anhydrous alumina columns using an Innovative Technology Inc. PS‐400‐7 solvent purification system. Solvents were deoxygenated either by channelling a stream of N_2_ through the liquid (sparging), or by the freeze‐thaw‐pump method. Thin layer chromatography (TLC) was carried out on aluminium plates coated with silica gel (Alugram®SIL G/UV 254 nm), and visualisation was achieved with UV light or KMnO_4_, ceric ammonium molybdate and iodine dips, followed by gentle heating. Solvents were removed using Büchi rotary evaporators and with high vacuum on a Schlenk line. Flash column chromatography was carried out using Davisil LC 60 Å silica gel (35–70 micron) purchased from Sigma‐Aldrich. NMR spectra were run in CDCl_3_ unless otherwise stated, on Bruker Avance 250, Bruker Avance 300, Bruker Avance 400, Bruker Avance 500 II+ or Agilent A500a instruments. IR spectra were recorded on a Perkin‐Elmer 1600 FT‐IR instrument. Capillary melting points were recorded on a Büchi 535 melting point apparatus, and are uncorrected. High resolution mass spectrometry (HRMS) was carried out using a micrOTOF ESI‐TOF spectrometer coupled to an Agilent 1200 LC system for autosampling. X‐ray crystallography was carried out on a Nonius Kappa CCD diffractometer with Mo−Kα radiation (λ=0.71073 Å).

### Synthetic Procedures

(1R*,4 S*,4aS*,8R*,8aR*,10R*)‐8,10‐Dihydroxy‐8,10‐bis(hydroxymethyl)‐4,4a,8,8a‐tetrahydro‐1,4‐ethanonaphthalene‐7,9(1H)‐dione (±)‐**11**. This was synthesised according to a literature procedure.^48^ Bis(epoxide) (±)‐**10** 49 (240 mg, 1.00 mmol) was dissolved in water (10 mL) and stirred at 60 °C for 48 h. Toluene (2 mL) was added and the combined solvents were removed *in vacuo*. No further purification was conducted.

*General procedure for synthesis of analogues* (±)‐**12** to (±)‐**17**. To crude (±)‐**11** (280 mg, 1.00 mmol, 1 eq.) in dichloromethane at room temperature was added the relevant acyl chloride, as well as DMAP, trimethylamine, and/or pyridine. The resulting solution was stirred, then the solvent was removed *in vacuo* and the residue partitioned between ethyl acetate (25 mL) and water (25 mL). The organic layer was washed further, then dried over MgSO_4_, filtered and the solvent removed *in vacuo*. The crude mixture was then purified by column chromatography (SiO_2_), and then recrystallized if necessary to give the desired product.

((1R*,4 S*,4aS*,8R*,8aR*,10R*)‐8,10‐Dihydroxy‐7,9‐dioxo‐1,4,4a,7,8,8a‐hexahydro‐1,4‐ethanonaphthalene‐8,10‐diyl)bis(methylene) bis(4‐fluorobenzoate) (±)‐**12**. The general procedure was employed, using 10 mL of CH_2_Cl_2_, para‐fluorobenzoyl chloride (0.26 mL, 2.20 mmol, 2.2 eq.), DMAP (10 mg, 0.05 mmol, 0.05 eq.) and the dropwise addition of triethylamine (0.56 mL, 4.00 mmol, 4 eq.). Reaction time was 24 h at room temperature. Further washes employed HCl_(aq)_ (0.1 M, 25 mL), water (25 mL), brine (25 mL). Chromatography eluent was petrol:EtOAc (4:1→1:1), and subsequent recrystallization from chloroform and petrol gave the desired product as a white solid, 90 mg (17 % yield): R_*f*_ 0.33 (petrol:EtOAc 1 : 1); m.p. 185–189 °C (crystallised from petrol:EtOAc); ν_max_/cm^−1^ (ATR) 3461 (bs), 1724 (s), 1687 (s); ^1^H NMR (CDCl_3_, 500 MHz) δ ppm 8.09‐8.05 (m, 2H), 7.96–7.93 (m, 2H), 7.15–7.12 (m, 2H), 7.11‐7.08 (m, 2H), 6.59 (dd, *J*=10.1, 4.3 Hz, 1H), 6.43 (ddd, *J*=8.0, 6.6, 1.3 Hz, 1H), 6.21 (dd, *J*=10.1, 1.6 Hz, 1H), 6.02 (ddd, *J*=7.9, 6.1, 1.6 Hz, 1H), 4.45 (d, *J*=11.9 Hz, 1H), 4.40 (d, *J*=11.2 Hz, 1H), 4.36 (d, *J*=11.2 Hz, 1H) 4.31 (s, 1H), 4.22 (d, *J*=11.9 Hz, 1H), 3.66 (dt, *J*=6.7, 1.9 Hz, 1H), 3.44 (ddt, *J*=8.4, 4.2, 2.0 Hz, 1H), 3.39 (ddd, *J*=6.2, 2.3, 1.3 Hz, 1H), 3.35 (dd, *J*=8.5, 2.2 Hz, 1H), 3.21 (br s, 1H); ^13^C NMR (CDCl_3_, 126 MHz) δ ppm 208.2, 198.2, 166.23 (d, ^1^ *J*_CF_=254 Hz), 166.15 (d, ^1^ *J*_CF_=254 Hz), 165.9, 165.0, 146.8, 135.2, 132.63 (d, ^3^ *J*_CF_=9.5 Hz), 132.45 (d, ^3^ *J*_CF_=9.4 Hz), 128.7, 128.2, 125.70 (d, ^4^ *J*_CF_=3.2 Hz), 125.67 (d, ^4^ *J*_CF_=3.1 Hz), 115.90 (d, ^2^ *J*_CF_=22.2 Hz), 115.78 (d, ^2^ *J*_CF_=22.1 Hz), 75.5, 74.6, 68.5, 52.3, 41.2, 40.1, 37.4; ^19^F NMR (CDCl_3_, 471 MHz) δ ppm −104.6 (s, 1F), −104.8 (s, 1F); ESI‐MS [M+Na]^+^ *m/z* C_28_H_22_F_2_NaO_8_ requires 547.1180, found 547.1155.

((1R*,4 S*,4aS*,8R*,8aR*,10R*)‐8,10‐Dihydroxy‐7,9‐dioxo‐1,4,4a,7,8,8a‐hexahydro‐1,4‐ethanonaphthalene‐8,10‐diyl)bis(methylene) bis(4‐(trifluoromethyl)benzoate) (±)‐**13**. The general procedure was employed, using 20 mL of CH_2_Cl_2_, *para*‐(trifluoromethyl)benzoyl chloride (0.30 mL, 2.00 mmol, 2.0 eq.), and pyridine (0.32 mL, 4.00 mmol, 4 eq.). Reaction time was 1 h at 0 °C and then 4 h at room temperature. Further washes employed NaHCO_3(aq)_ (saturated, 2×20 mL), water (20 mL), brine (20 mL). Chromatography eluent was petrol:EtOAc (6:1→1:1), and subsequent recrystallization from chloroform gave the desired product as a white solid, 60 mg (10 % yield): R_*f*_ 0.72 (1 : 1 petrol:ethyl acetate); m.p. 186–188 °C (crystallised from petrol:EtOAc); ν_max_/cm^−1^ (ATR) 3422 (bs), 1712 (s), 1710 (s); ^1^H NMR (CDCl_3_, 500 MHz) δ ppm 8.17 (br d, *J*=8.1 Hz, 2H), 8.04 (br d, *J*=8.2 Hz, 2H), 7.74 (br d, *J*=8.2 Hz, 2H), 7.69 (br d, *J*=8.5 Hz, 2H), 6.61 (dd, *J*=10.1, 4.3 Hz, 1H), 6.44 (ddd, *J*=8.1, 6.6, 1.3 Hz, 1H), 6.22 (dd, *J*=10.1, 1.6 Hz, 1H), 6.04 (ddd, *J*=7.8, 6.1, 1.6 Hz, 1H), 4.50 (d, *J*=11.9 Hz, 1H), 4.43 (d, *J*=11.2 Hz), 4.40 (d, *J*=11.2 Hz), 4.33 (s, 1H), 4.26 (d, *J*=11.9 Hz, 1H), 3.68 (dt, *J*=6.6, 1.9 Hz, 1H), 3.45 (ddt, *J*=8.3, 4.1, 1.9 Hz, 1H), 3.41 (ddd, *J*=6.1, 2.3, 1.3 Hz, 1H), 3.36 (dt, *J*=8.5, 2.2 Hz, 1H), 3.15 (s, 1H); ^13^C NMR (CDCl_3_, 126 MHz) δ ppm 208.2, 198.0, 165.6, 164.7, 146.9, 135.11, 135.10 (q, ^2^ *J*_CF_=32.7 Hz), 135.01 (q, ^2^ *J*_CF_=32.7 Hz), 132.69 (q, ^4^ *J*_CF_=1.1 Hz), 132.61 (q, ^4^ *J*_CF_=1.0 Hz), 130.4, 130.3, 128.8, 128.2, 125.74 (q, ^3^ *J*_CF_=3.8 Hz), 125.69 (q, ^3^ *J*_CF_=3.5 Hz), 123.67 (q, ^1^ *J*_CF_=272 Hz, 2 C), 75.5, 74.5, 72.2, 68.8, 52.3, 41.1, 40.1, 37.4; ^19^F NMR (CDCl_3_, 471 MHz) δ ppm −63.23 (s, 3F), −63.25 (s, 3F); ESI‐MS [M+Na]^+^ *m/z* C_30_H_22_F_6_NaO_8_ requires 647.1117, found 647.1100.

((1R*,4 S*,4aS*,8R*,8aR*,10R*)‐8,10‐Dihydroxy‐7,9‐dioxo‐1,4,4a,7,8,8a‐hexahydro‐1,4‐ethanonaphthalene‐8,10‐diyl)bis(methylene) bis(4‐(trifluoromethyl)benzoate) (±)‐**14**. The general procedure was employed, using 10 mL of CH_2_Cl_2_, 3,4,5‐trimethoxybenzoyl chloride (0.51 g, 2.20 mmol, 2.2 eq.), DMAP (10 mg, 0.05 mmol, 0.05 eq.) and triethylamine (0.56 mL, 4.00 mmol, 4 eq.). Reaction time was 22 h at room temperature. Further washes employed HCl_(aq)_ (0.1 M, 25 mL), water (25 mL), brine (25 mL). Chromatography eluent was petrol:EtOAc (2:1→1:1), and subsequent recrystallization from dichloromethane gave the desired product as a white solid, 70 mg (10 % yield): R_*f*_ 0.16 (petrol:EtOAc 1 : 1); m.p. 171–173 °C (dec., crystallised from dichloromethane); ν_max_/cm^−1^ (ATR) 3446 (bs), 1716 (s), 1588 (s); ^1^H NMR (CDCl_3_, 500 MHz) δ ppm 7.30 (s, 2H), 7.19 (s, 2H), 6.60 (dd, *J*=10.1, 4.2 Hz, 1H), 6.43 (ddd, *J*=8.0, 6.5, 1.3 Hz, 1H), 6.21 (dd, *J*=10.1, 1.6 Hz, 1H), 6.02 (ddd, *J*=7.9, 6.0, 1.5 Hz, 1H), 4.43 (d, *J*=11.9 Hz, 1H), 4.38 (d, *J*=11.1 Hz, 1H), 4.32 (d, *J*=11.2 Hz, 1H), 4.30 (s, 1H), 4.25 (d, *J*=11.9 Hz, 1H), 3.92 (s, 6H), 3.91 (s, 3H), 3.89 (s, 3H), 3.89 (s, 6H), 3.65 (dt, *J*=6.6, 1.9 Hz, 1H), 3.45–3.43 (m, 1H), 3.39 (ddd, *J*=6.2, 2.3, 1.4 Hz, 1H), 3.36 (dd, *J*=8.5, 2.1 Hz), 3.34 (s, 1H); ^13^C NMR (CDCl_3_, 126 MHz) δ ppm 208.2, 198.3, 166.6, 165.5, 153.2, 153.1, 146.8, 142.9, 142.7, 135.2, 128.6, 128.2, 124.3, 107.3, 107.1, 75.6, 74.7, 72.0, 68.7, 61.10, 61.07, 56.5, 56.45, 56.36, 52.4, 41.2, 40.2, 37.3; ESI‐MS [M+Na]^+^ *m/z* C_34_H_36_NaO_14_ requires 691.2003, found 691.1962.

((1R*,4 S*,4aS*,8R*,8aR*,10R*)‐8,10‐Dihydroxy‐7,9‐dioxo‐1,4,4a,7,8,8a‐hexahydro‐1,4‐ethanonaphthalene‐8,10‐diyl)bis(methylene) dicyclopentanecarboxylate (±)‐**15**. The general procedure was employed, using 10 mL of CH_2_Cl_2_, 3,4,5‐ cyclopentane carbonyl chloride (0.27 mL, 2.20 mmol, 2.2 eq.), DMAP (10 mg, 0.05 mmol, 0.05 eq.) and triethylamine (0.56 mL, 4.00 mmol, 4 eq.). Reaction time was 16 h at 40 °C. Further washes employed HCl_(aq)_ (0.1 M, 25 mL), water (25 mL), brine (25 mL). Chromatography eluent was CH_2_Cl_2_:EtOAc (9 : 1), giving the desired product as a white solid, 90 mg (19 % yield): R_*f*_ 0.38 (9 : 1 CH_2_Cl_2_:EtOAc) m.p. 149–153 °C (crystallised from CH_2_Cl_2_:EtOAc); ν_max_/ cm^−1^ (ATR) 3435 (bs), 1726 (s), 1687 (s); ^1^H NMR (CDCl_3_, 500 MHz) δ ppm 6.53 (dd, *J*=10.1, 4.3 Hz, 1H), 6.33 (ddd, *J*=8.0, 6.6, 1.3 Hz, 1H), 6.14 (dd, *J*=10.1, 1.6 Hz, 1H), 5.95 (ddd, *J*=7.9, 6.0, 1.6 Hz, 1H), 4.25 (d, *J*=11.9 Hz, 1H), 4.20 (d, *J*=11.2 Hz, 1H), 4.17 (s, 1H), 4.08 (d, *J*=11.2 Hz, 1H), 3.92 (d, *J*=11.9 Hz, 1H), 3.50 (dt, *J*=6.8, 1.9 Hz, 1H), 3.36 (ddt, *J*=8.5, 4.2, 2.0 Hz, 1H), 3.30 (ddd, *J*=6.1, 2.3, 1.3 Hz, 1H), 3.23–3.20 (m, 2H), 2.77 (p, *J*=8.4 Hz, 1H), 2.66 (tt, *J*=8.5, 6.8 Hz, 1H), 1.90 (dddd, *J*=14.5, 8.7, 7.5, 3.8 Hz, 2H), 1.84–1.77 (m, 4H), 1.74–1.65 (m, 6H), 1.60–1.55 (m, 4H); ^13^C NMR (CDCl_3_, 126 MHz) δ ppm 208.2, 198.2, 177.2, 176.2, 146.5, 135.1, 128.6, 128.2, 75.6, 74.6, 71.2, 67.7, 52.3, 43.79, 43.74, 41.1, 40.1, 37.3, 30.20, 30.16, 30.15, 30.02, 25.91 (2 C), 25.85, 25.84; ESI‐MS [M+Na]^+^ *m/z* C_26_H_32_NaO_8_ requires 495.1989, found 495.2023.

((1R*,4 S*,4aS*,8R*,8aR*,10R*)‐8,10‐Dihydroxy‐7,9‐dioxo‐1,4,4a,7,8,8a‐hexahydro‐1,4‐ethanonaphthalene‐8,10‐diyl)bis(methylene) dicyclopentanecarboxylate (±)‐**16**. The general procedure was employed, using 10 mL of CH_2_Cl_2_, 3,4,5‐ pivaloyl chloride (0.27 mL, 2.20 mmol, 2.2 eq.), DMAP (10 mg, 0.05 mmol, 0.05 eq.) and triethylamine (0.56 mL, 4.00 mmol, 4 eq.). Reaction time was 20 h at room temperature. Further washes employed HCl_(aq)_ (0.1 M, 25 mL), water (25 mL), brine (25 mL). Chromatography eluent was CH_2_Cl_2_:MeOH (99 : 1), giving the desired product as a white solid, 80 mg (18 % yield): R_*f*_ 0.23 (CH_2_Cl_2_:MeOH 99 : 1); m.p. 158–161 °C (crystallised from CH_2_Cl_2_:MeOH); ν_max_/cm^−1^ (ATR) 3451 (bs), 1719 (s), 1695 (s); ^1^H NMR (CDCl_3_, 500 MHz) δ ppm 6.54 (dd, *J*=10.1, 4.3 Hz, 1H), 6.34 (ddd, *J*=8.2, 6.6, 1.3 Hz, 1H), 6.14 (dd, *J*=10.1, 1.7 Hz, 1H), 5.96 (ddd, *J*=7.9, 6.1, 1.6 Hz, 1H), 4.25 (d, *J*=11.9 Hz, 1H), 4.19 (d, *J*=11.2 Hz, 1H), 4.17 (s, 1H), 4.07 (d, *J*=11.2 Hz, 1H), 3.92 (d, *J*=11.9 Hz, 1H), 3.50 (dt, *J*=6.6, 2.0 Hz, 1H), 3.36 (ddt, *J*=8.4, 4.0, 1.8 Hz, 1H), 3.31 (ddd, *J*=6.1, 2.3, 1.3 Hz, 1H), 3.22 (dd, *J*=8.5, 2.2 Hz, 1H), 3.15 (s, 1H), 1.22 (s, 9H), 1.13 (s, 9H); ^13^C NMR (CDCl_3_, 126 MHz) δ ppm 208.2, 198.1, 179.0, 178.0, 146.5, 135.1, 128.6, 128.1, 75.6, 74.6, 71.3, 67.9, 52.3, 41.1, 40.1, 39.1, 38.9, 37.3, 27.3, 27.2; ESI‐MS [M−H]^−^ *m/z* C_24_H_31_O_8_ requires 447.2024, found 447.2043.

(1R*,4 S*,4aS*,8R*,8aR*,10R*)‐8,10‐Dihydroxy‐10‐(hydroxymethyl)‐7,9‐dioxo‐1,4,4a,7,8,8a‐hexahydro‐1,4‐ethanonaphthalen‐8‐yl)methyl nicotinate (±)‐ **17**. The general procedure was employed, using 20 mL of CH_2_Cl_2_, 3,4,5‐ nicotinoyl chloride (0.39 mL, 2.20 mmol, 2.2 eq.), DMAP (10 mg, 0.05 mmol, 0.05 eq.) and triethylamine (0.56 mL, 4.00 mmol, 4 eq.). Reaction time was 22 h at room temperature. Further washes employed HCl_(aq)_ (0.1 M, 25 mL), water (25 mL), brine (25 mL). Chromatography eluent was CH_2_Cl_2_:MeOH (99 : 1), giving the desired product as a cream‐coloured solid, 110 mg (29 % yield): R_*f*_ 0.56 (CH_2_Cl_2_:MeOH 99 : 1) m.p. 220–224 °C (crystallised from CH_2_Cl_2_:methanol); ν_max_/cm^−1^ (ATR) 3460 (s), 1731 (s), 1688 (s); ^1^H NMR (CD_2_Cl_2_, 500 MHz) δ ppm 9.07 (dd, *J*=2.2, 0.9 Hz, 1H), 8.76 (dd, *J*=4.9, 1.7 Hz, 1H), 8.18 (dd, *J*=7.9, 2.2, 1.7 Hz, 1H), 7.39 (ddd, *J*=8.0, 4.8, 0.9 Hz, 1H), 6.66 (dd, *J*=10.2, 4.2 Hz, 1H), 6.43 (dddd, *J*=8.1, 6.6, 1.5, 0.6 Hz, 1H), 6.22 (dd, *J*=10.1, 1.8 Hz, 1H), 6.03 (dddd, *J*=8.1, 6.3, 1.7, 0.6 Hz, 1H), 4.44 (d, *J*=11.2 Hz, 1H), 4.40 (d, *J*=11.2 Hz, 1H), 4.32 (s, 1H), 3.53 (ddddd, *J*=8.5, 4.3, 2.4, 1.8, 0.6 Hz, 1H), 3.48 (ddd, *J*=6.3, 2.5, 1.4 Hz, 1H), 3.14 (dt, *J*=6.6, 2.0 Hz, 1H), 3.10 (d, *J*=6.2 Hz, 1H), 3.06 (dd, *J*=8.5, 2.2 Hz, 1H), 2.92 (d, *J*=6.1 Hz, 1H). ^13^C NMR (CD_2_Cl_2_, 126 MHz) δ ppm 204.2, 198.1, 165.0, 154.4, 151.4, 147.8, 137.5, 134.8, 130.0, 128.5, 125.9, 123.9, 76.0, 72.3, 58.6, 54.4, 53.7, 41.5, 40.2, 39.5; ESI‐MS [M−H]^−^ *m/z* C_20_H_18_NO_6_ requires 368.1129, found 368.1144.

(1′R*,2R*,4′S*)‐7′‐Hydroxy‐8′‐(hydroxymethyl)‐1′,4′‐dihydrospiro[oxirane‐2,10′‐[1,4]ethanonaphthalen]‐9′‐one (±)‐**19**. Bis(epoxide) (±)‐**10** ^35^ (240 mg, 1.00 mmol, 1 eq.) was dissolved in anhydrous THF (12 mL) and DBU (0.30 mL, 2.00 mmol, 2 eq.) was added dropwise. The resulting mixture was stirred at room temperature for 24 h, then HCl_(aq)_ (1.0 M, 10 mL) was added and then extracted with EtOAc (2×20 mL). The combined organic layers were washed with brine (10 mL), then dried over MgSO_4_, filtered and the solvent removed *in vacuo*. The crude product was purified by column chromatography (SiO_2_) eluting with petrol:EtOAc (1 : 1) to give (±)‐**19** as a white solid, 120 mg (51 % yield): R_*f*_ 0.23 (Petrol:EtOAc 1 : 1); m.p. 186–191 °C (crystallised from petrol:EtOAc); ν_max_/cm^−1^ (ATR) 3445 (bs), 3199 (bs), 1729; ^1^H NMR (CD_3_CN, 500 MHz) δ ppm 7.83 (s, 1H), 7.16 (d, *J*=8.0 Hz, 1H), 6.79–6.75 (m, 2H), 6.71 (d, *J*=8.1 Hz, 1H), 4.82 (dd, *J*=12.6, 5.2 Hz, 1H), 4.75 (dd, *J*=12.6, 5.6 Hz, 1H), 4.43 (dd, *J*=5.4, 2.2 Hz, 1H), 4.09 (dd, *J*=5.9, 2.1 Hz, 1H), 3.39 (t, *J*=5.4 Hz, 1H), 3.03 (d, *J*=5.9 Hz, 1H), 2.97 (d, *J*=5.9 Hz, 1H); ^13^C NMR (CD_3_CN, 126 MHz) δ ppm 199.3, 156.6, 140.5, 136.0, 133.4, 128.3, 126.4, 124.0, 115.0, 57.9, 57.5, 57.1, 53.5, 43.6; ESI‐MS [M+Na]^+^ *m/z* C_14_H_12_NaO_4_ requires 267.0633, found 267.0617.

(1′R*,2R*,4′S*)‐7′‐Hydroxy‐1′,4′‐dihydrospiro[oxirane‐2,10′‐[1,4]ethanonaphthalen]‐9′‐one (±)‐**21**. Bis(epoxide) (±)‐**10** ^35^ (240 mg, 1.00 mmol, 1 eq.) was dissolved in methanol (10 mL) and stirred at 0 °C. Sodium methoxide (0.5 M in MeOH, 4.40 mL, 2.20 mmol, 2.2 eq.) was then added dropwise and the reaction mixture stirred for 16 h at room temperature. The reaction mixture was extracted with dichloromethane (3×10 mL), washed with NaHCO_3(aq)_ (saturated, 10 mL), water (10 mL), and brine (10 mL), then dried over MgSO_4_, filtered and the solvent removed *in vacuo*. The crude product was purified by column chromatography (SiO_2_), eluting with petrol:EtOAc (1:1→1:2) to give (±)‐**21** as a white solid, 80 mg (41 % yield). R_*f*_ 0.47 (petrol:EtOAc 1 : 1); m.p. 170–174 °C (crystallised from petrol:EtOAc); ν_max_/cm^−1^ (ATR) 3345 (bs), 1731 (s); ^1^H NMR (CD_3_OD, 500 MHz) δ ppm 7.16 (d, *J*=8.1 Hz, 1H), 6.82 (d, *J*=2.4 Hz, 1H), 6.79 (ddd, *J*=7.8, 6.2, 1.6 Hz, 1H), 6.72 (ddd, *J*=7.7, 6.0, 1.8 Hz, 1H), 6.67 (dd, *J*=8.1, 2.4 Hz, 1H), 4.40 (ddd, *J*=6.0, 1.6, 0.5 Hz, 1H), 3.65 (dd, *J*=6.2, 1.8 Hz, 1H), 2.99 (d, *J*=6.0 Hz, 1H), 2.98 (d, *J*=6.0 Hz, 1H); ^13^C NMR (CD_3_OD, 126 MHz) δ ppm 200.0, 158.5, 142.5, 136.3, 133.3, 127.6, 127.0, 114.6, 113.8, 57.6, 57.5, 53.4, 48.0; ESI‐MS [M+Na]^+^ *m/z* C_13_H_10_NaO_3_ requires 237.0522, found 237.0521.

(1′R*,4R*,4′R*,4a′R*,5′R*,8′S*,8a′S*)‐4′‐Hydroxy‐1′,4′,4a′,5′,8′,8a′‐hexahydrospiro[[1,3]dioxolane‐4,10′‐[1,4](epoxymethano)[5,8]ethanonaphthalene]‐2,3′,9′(2′H)‐trione (±)‐**27**. Crude tetraol (±)‐**11** (280 mg, 1.00 mmol, 1 eq.) was stirred in anhydrous dichloromethane (10 mL) and carbonyl diimidazole (370 mg, 2.3 mmol, 2.3 eq.) was added. The reaction mixture was stirred for 18 h at room temperature, water (10 mL) was added and then extracted with dichloromethane (2×10 mL), washed with brine (10 mL), dried over MgSO_4_, filtered and the solvent removed *in vacuo*. The crude product was purified by column chromatography (SiO_2_), eluting with (CH_2_Cl_2_:MeOH 9 : 1) to give (±)‐**27** as a white crystalline solid, 130 mg (45 % yield). R_*f*_ 0.35 (CH_2_Cl_2_:MeOH 9 : 1); m.p. 200–206 °C (crystallised from CH_2_Cl_2_:MeOH); ν_max_/cm^−1^ (ATR) 2927 (bs), 1805 (s), 1726 (s); ^1^H NMR (CD_3_CN, 500 MHz) δ ppm 6.26 (dddd, *J*=7.0, 6.2, 1.7, 0.8 Hz, 1H), 6.05 (tt, *J*=6.9, 1.0 Hz, 1H), 4.21 (app q, *J*=3.4 Hz, 1H), 4.18 (d, *J*=8.8 Hz, 1H), 4.12 (d, *J*=8.8 Hz, 1H), 3.91 (s, 1H), 3.80 (dd, *J*=8.8, 1.0 Hz, 1H), 3.56 (d, *J*=8.9 Hz, 1 H), 3.55 (dt, *J*=6.9, 2.0 Hz, 1H), 3.43 (dt, *J*=6.3, 1.6 Hz, 1H), 3.05‐2.99 (m, 2H), 2.71 (ddd, *J*=19.7, 3.2, 1.2 Hz, 1H), 2.50 (ddd, *J*=19.7, 2.1, 1.0 Hz, 1H); ^13^C NMR (CD_3_CN, 126 MHz) δ ppm 210.7, 202.4, 154.9, 132.1, 131.8, 80.4, 75.1, 72.3, 71.5, 70.7, 48.9, 43.9, 42.3, 41.8, 40.1; ESI‐MS [M−H−H_2_O]^−^ *m/z* C_15_H_11_O_6_ requires 287.0561, found 287.0564.

(1R*,4 S*,4aS*,8R*,8aR*,10R*)‐8,10‐Bis(azidomethyl)‐8,10‐dihydroxy‐4,4a,8,8a‐tetrahydro‐1,4‐ethanonaphthalene‐7,9(1H)‐dione (±)‐**30**. To a stirred solution of 2‐(azidomethyl)phenol **29** 44 (0.22 g, 1.49 mmol, 1 eq.) in anhydrous THF (10 mL) was added stabilised 2‐iodoxybenzoic acid (SIBX) (1.85 g, 2.98 mmol of IBX, 2 eq.) as a solid in one portion. The resulting suspension was stirred at room temperature for 16 h, after which trifluoroacetic acid (0.68 mL, 8.94 mmol, 1 eq.) was added and stirred for a further 4 h. The reaction mixture was diluted with CH_2_Cl_2_ (100 mL) and H_2_O (50 mL). NaHCO_3(aq)_ was added slowly, with shaking, until all the acid had been neutralised. The aqueous phase was extracted with CH_2_Cl_2_ (2×20 mL). The combined organic phases were then washed with NaHCO_3(aq)_ (20 mL), H_2_O (20 mL), brine (20 mL) and then vigorously shaken with Na_2_S_2_O_3(aq)_ (1.0 M, 20 mL), washed again with brine (20 mL) and dried over MgSO_4_, filtered and the solvent evaporated *in vacuo* to give a dark brown oil. The crude mixture was then purified by column chromatography (SiO_2_), eluting with CH_2_Cl_2_/MeOH 99 : 1 to give the desired product as a light brown oil, 30 mg (12 % yield): R_*f*_ 0.22 (petrol:EtOAc 1 : 1); ν_max_/cm^−1^ (ATR) 3447 (bs), 2100 (s), 1727 (s), 1690 (s); ^1^H NMR (CDCl_3_, 500 MHz) δ ppm 6.55 (dd, *J*=10.1, 4.0 Hz, 1H), 6.33 (ddd, *J*=8.1, 6.6, 1.2 Hz, 1H), 6.20 (dd, *J*=10.1, 1.5 Hz, 1H), 5.95 (ddd, *J*=8.2, 6.6, 1.6 Hz, 1H), 4.31 (s, 1H), 3.60 (dt, *J*=6.7, 1.9 Hz, 1H), 3.43 (d, *J*=12.5 Hz, 1H), 3.37 (d, *J*=13.0 Hz, 1H), 3.36‐3.32 (m, 2H), 3.29‐3.27 (m, 1H), 3.27 (d, *J*=12.4 Hz, 1H), 3.20 (d, *J*=12.9 Hz, 1H), 2.87 (br s, 1H); ^13^C NMR (CDCl_3_, 126 MHz) δ ppm 208.7, 198.4, 146.8, 135.3, 128.8, 128.1, 77.4, 75.2, 61.4, 57.1, 52.2, 41.6, 39.9, 38.4; ESI‐MS [M+Na]^+^ *m/z* C_14_H_14_N_6_NaO_4_ requires 353.0969, found 353.0993.

### Biological Evaluation

#### Preferential Cytotoxicity Assay Against PANC‐1 Cells

The human pancreatic cancer cell line PANC‐1 (RBRC‐RCB2095, Tsukuba, Japan) was purchased from the Riken BRC cell bank and maintained in standard Dulbecco's modified Eagle's medium (DMEM) supplemented with 10 % foetal bovine serum (FBS) at 37 °C under a humidified atmosphere of 5 % CO_2_ incubator. For preferential cytotoxicity assay, PANC‐1 cells were seeded in 96‐well plates (1.5×10^4^/well) in DMEM and incubated overnight CO_2_ incubator for the cell attachment. The cells were then washed twice with phosphate‐buffered saline (PBS), and then added serially diluted test samples in both nutrient‐rich medium (DMEM) and nutrient‐deprived medium (NDM) with a control and blank in each test plate. The treated plates were incubated for 24 h. The medium in each well was then replaced by 100 μL of DMEM containing 10 % WST‐8 cell counting kit solution, followed by 3 h of incubation, and the absorbance at 450 nm was measured on an EnSpire Multimode plate reader (PerkinElmer, Inc., Waltham, MA, USA). Cell viability was calculated from the mean values from three wells using the following equation:

$$\mathrm{Cell}\;\mathrm{viability}\;\left(\%\right)=\;\left[\mathrm{Abs}{}_{\left(\mathrm{test}\;\mathrm{sample}\right)}-\mathrm{Abs}{}_{\left(\mathrm{blank}\right)}/\mathrm{Abs}{}_{\left(\mathrm{control}\right)}-\mathrm{Abs}{}_{\left(\mathrm{blank}\right)}\right]\times 100\;\%$$

### Morphological Assessment of Cancer Cells

PANC‐1 cells were seeded at a density of 2×10^5^ cells/well in DMEM in a 12‐well plate dish and incubated overnight in a CO_2_ incubator for the cell attachment. The cells were then washed twice with PBS, followed by treatment with (±)‐12 (10 mM) in NDM, or, untreated control cells in NDM. The cells were then incubated for 24 h, and then treated with EB/AO reagent, and cell morphology was captured using Evos FL digital microscope (20× objective) with phase‐contrast and fluorescence modes.

### Colony Formation Assay

PANC‐1 cells were seeded at a density of 2 × 10^3^ cells/well in DMEM (2 mL/well) in a 12‐well plate dish and incubated for 24 h for the cell attachment. The medium was then changed to DMEM containing (±)‐12 at 100, 50, 25, or untreated control in DMEM, with three replicates in each group, and incubated for 24 h. The cells were then washed twice with PBS and the medium was replaced by fresh DMEM (2 mL) without any test compound. Then the cells were allowed to grow for 10 days. On the last day, the cells were washed with PBS, fixed with 4 % formaldehyde, and stained with crystal violet for 10 min. Finally, the colony area was measured using ImageJ plugin “Colony Area”,^60^ and the data were analysed by the GraphPad software Prism 6.

### Time‐Lapse Imaging

PANC‐1 cells (1×10^5^) were plated in 35‐mm cell culture dishes and allowed to attach overnight in DMEM. The cells were then washed with PBS, followed by treatment with 10 μM (±)‐12 in nutrient‐deprived medium (NDM), and placed immediately inside a humidified CO_2_ incubator at 37 °C, equipped real‐time digital time‐lapse microscopy system for the both control and treated group in parallel. Live images were captured for every 15 min for 24 h.

## Conflict of interest

The authors declare no conflict of interest.

## Supporting information

As a service to our authors and readers, this journal provides supporting information supplied by the authors. Such materials are peer reviewed and may be re‐organized for online delivery, but are not copy‐edited or typeset. Technical support issues arising from supporting information (other than missing files) should be addressed to the authors.

Supplementary

Supplementary

## References

[1] M. Hidalgo , N. Engl. J. Med. 2010, 362, 1605. 20427809 10.1056/NEJMra0901557

[2] E. M. O′Reilly , Gastrointest. Cancer Res. 2009, 3, S11. PMC2684727 19461915

[3] H. Tsukuma , W. Ajiki , A. Ioka , A. Oshima , Jpn. J. Clin. Oncol. 2006, 36, 602. 16870692 10.1093/jjco/hyl068

[4] C. R. Smittenaar , K. A. Petersen , K. Stewart , N. Moitt , Brit. J. Cancer 2016, 115, 1147. 27727232 10.1038/bjc.2016.304 PMC5117795

[5] M. T. Saung , L. Zheng , Clin. Ther. 2017, 39, 2125. 28939405 10.1016/j.clinthera.2017.08.015 PMC5705388

[6] H. H. Wong , N. R. Lemoine , Nat. Rev. Gastroenterol. Hepatol. 2009, 6, 412. 19506583 10.1038/nrgastro.2009.89 PMC2882232

[7] K. Izuishi , K. Kato , T. Ogura , T. Kinoshita , H. Esumi , Cancer Res. 2000, 60, 6201. 11085546

[8] J. Lu , S. Kunimoto , Y. Yamazaki , M. Kaminishi , H. Esumi , Cancer Sci. 2004, 95, 547. 15182438 10.1111/j.1349-7006.2004.tb03247.x PMC11158080

[9] S. Awale , J. Lu , S. K. Kalauni , Y. Kurashima , Y. Tezuka , S. Kadota , H. Esumi , Cancer Res. 2006, 66, 1751. 16452235 10.1158/0008-5472.CAN-05-3143

[10] J. Magolan , M. J. Coster , Curr. Drug Delivery 2010, 7, 355. 10.2174/156720110793566272 20973759

[11] H. Esumi , J. Lu , Y. Kurashima , T. Hanaoka , Cancer Sci. 2004, 95, 685. 15298733 10.1111/j.1349-7006.2004.tb03330.x PMC11159109

[12] J. Magolan , N. B. P. Adams , H. Onozuka , N. L. Hungerford , H. Esumi , M. J. Coster , ChemMedChem 2012, 7, 766. 22431333 10.1002/cmdc.201100564

[13] C. M. Farley , D. F. Dibwe , J.-Y. Ueda , E. A. Hall , S. Awale , J. Magolan , Bioorg. Med. Chem. Lett. 2016, 26, 1471. 26832787 10.1016/j.bmcl.2016.01.054

[14] J. Magolan , M. J. Coster , J. Org. Chem. 2009, 74, 5083. 19459593 10.1021/jo900613u

[15] T. Devji , C. Reddy , C. Woo , S. Awale , S. Kadota , D. Carrico-Moniz , Bioorg. Med. Chem. Lett. 2011, 21, 5770. 21880488 10.1016/j.bmcl.2011.08.005

[16] M. Jun , A. F. Bacay , J. Moyer , A. Webb , D. Carrico-Moniz , Bioorg. Med. Chem. Lett. 2014, 24, 4654. 25205194 10.1016/j.bmcl.2014.08.038

[17] P. A. Turner , E. M. Griffin , J. L. Whatmore , M. Shipman , Org. Lett. 2011, 13, 1056. 21268595 10.1021/ol103103n

[18] P. A. Turner , Samiullah , J. L. Whatmore , M. Shipman , Tetrahedron Lett. 2013, 54, 6538.

[19] S. Awale , T. Okada , D. F. Dibwe , T. Maruyama , S. Takahara , T. Okada , S. Endo , N. Toyooka , Bioorg. Med. Chem. Lett. 2019, 29, 1779. 31097375 10.1016/j.bmcl.2019.05.010

[20] S. Fayez , D. Feineis , V. Mudogo , S. Awale , G. Bringmann , RSC Adv. 2017, 7, 53740.

[21] S. M. Kavatsurwa , B. K. Lombe , D. Feineis , D. F. Dibwe , V. Maharaj , S. Awale , G. Bringmann , Fitoterapia 2018, 130, 6. 30059720 10.1016/j.fitote.2018.07.017

[22] S. Fayez , D. Feineis , L. A. Assi , M. Kaiser , R. Brun , S. Awale , G. Bringmann , Fitoterapia 2018, 131, 245. 30419265 10.1016/j.fitote.2018.11.006

[23] S. Awale , D. F. Dibwe , C. Balachandran , S. Fayez , D. Feineis , B. K. Lombe , G. Bringmann , J. Nat. Prod. 2018, 81, 2282. 30303002 10.1021/acs.jnatprod.8b00733

[24] B. K. Lombe , D. Feineis , V. Mudogo , R. Brun , S. Awale , G. Bringmann , RSC Adv. 2018, 8, 5243. 10.1039/c8ra00363g PMC9078195 35542436

[25] T. Masuda , S. Ohba , M. Kawada , M. Osono , D. Ikeda , H. Esumi , S. Kunimoto , J. Antibiot. 2006, 59, 209. 16830887 10.1038/ja.2006.29

[26] M. Ikeda , A. Sato , N. Mochizuki , K. Toyosaki , C. Miyoshi , R. Fujioka , S. Mitsunaga , I. Ohno , Y. Hashimoto , H. Takahashi , H. Hasegawa , S. Nomura , R. Takahashi , S. Yomoda , K. Tsuchihara , S. Kishino , H. Esumi , Cancer Sci. 2016, 107, 1818. 27685612 10.1111/cas.13086 PMC5198948

[27] H. Esumi, M. Ikeda, K. Tsuchihara, S. Chiba, S. Yomoda, T. Kawashima, T. Okubo, Y. Tezuka, K. Murata, “Anticancer agent, side-effect-alleviating agent”, 2018, US Patent #9907852B2.

[28] Y.-H. Liao , L.-Z. Xu , S.-L. Yang , J. Dai , Y.-S. Zhen , M. Zhu , N.-J. Sun , Phytochemistry 1997, 45, 729.

[29] Y.-H. Liao , Z.-M. Zhou , J. Guo , L.-Z. Xu , M. Zhu , S.-L. Yang , J. Chin. Pharm. Sci. 2000, 9, 170.

[30] C.-R. Zhang , S.-P. Yang , S.-G. Liao , Y. Wu , J.-M. Yue , Helv. Chim. Acta 2006, 89, 1408.

[31] G.-X. Zhou , Y.-J. Zhang , R.-Y. Chen , D.-Q. Yu , Nat. Prod. Res. Dev. 2007, 19, 433.

[32] M. J. Palframan , G. Kociok-Köhn , S. E. Lewis , Chem. Eur. J. 2012, 18, 4766. 22378592 10.1002/chem.201104035

[33] M. J. Palframan , G. Kociok-Köhn , S. E. Lewis , Org. Lett. 2011, 13, 3150. 21591657 10.1021/ol201057r

[34] S. E. Lewis , Chem. Commun. 2014, 50, 2821. 10.1039/c3cc49694e 24514270

[35] S. Awale , J.-Y. Ueda , S. Athikomkulchai , S. Abdelhamed , S. Yokoyama , I. Saiki , R. Miyatake , J. Nat. Prod. 2012, 75, 1177. 22676269 10.1021/np300295h

[36] S. Awale , J.-Y. Ueda , S. Athikomkulchai , D. F. Dibwe , S. Abdelhamed , S. Yokoyama , I. Saiki , R. Miyatake , J. Nat. Prod. 2012, 75, 1999. 23092429 10.1021/np300596c

[37] J.-Y. Ueda , S. Athikomkulchai , R. Miyatake , I. Saiki , H. Esumi , S. Awale , Drug Des. Dev. Ther. 2014, 8, 39. 10.2147/DDDT.S52168 PMC3872082 24379655

[38] Z. J. Yang , C. E. Chee , S. Huang , F. A. Sinicrope , Mol. Cancer Ther. 2011, 10, 1533. 21878654 10.1158/1535-7163.MCT-11-0047 PMC3170456

[39] K. S. Choi , Exp. Mol. Med. 2012, 44, 109. 22257886 10.3858/emm.2012.44.2.033 PMC3296807

[40] T. Kanzawa , I. M. Germano , T. Komata , H. Ito , Y. Kondo , S. Kondo , Cell Death Differ. 2004, 11, 448. 14713959 10.1038/sj.cdd.4401359

[41] K. Izuishi , K. Kato , T. Ogura , T. Kinoshita , H. Esumi , Cancer Res. 2000, 60, 6201. 11085546

[42] D. A. Guertin , D. M. Sabatini , Cancer Cell 2007, 12, 9. 17613433 10.1016/j.ccr.2007.05.008

[43] J. Lu , S. Kunimoto , Y. Yamazaki , M. Kaminishi , H. Esumi , Cancer Sci. 2004, 95, 547. 15182438 10.1111/j.1349-7006.2004.tb03247.x PMC11158080

[44] H. Esumi , J. Lu , Y. Kurashima , T. Hanaoka , Cancer Sci. 2004, 95, 685. 15298733 10.1111/j.1349-7006.2004.tb03330.x PMC11159109

[45] S. Awale , J. Lu , S. K. Kalauni , Y. Kurashima , Y. Tezuka , S. Kadota , H. Esumi , Cancer Res. 2006, 66, 1751. 16452235 10.1158/0008-5472.CAN-05-3143

[46] M. Ali Khan , P. J. Wood , N. M. Lamb-Guhren , L. Caggiano , G. Kociok-Köhn , D. Tosh , S. E. Lewis , Bioorg. Med. Chem. Lett. 2014, 24, 2815. 24835628 10.1016/j.bmcl.2014.04.111

[47] N. Lebrasseur , J. Gagnepain , A. Ozanne-Beaudenon , J.-M. Léger , S. Quideau , J. Org. Chem. 2007, 72, 6280. 17628111 10.1021/jo0708893

[48] M. Bergner , D. C. Duquette , L. Chio , B. M. Stoltz , Org. Lett. 2015, 17, 3008. 26060887 10.1021/acs.orglett.5b01292

[49] E. Adler , S. Brasen , H. Miyake , Acta Chem. Scand. 1971, 25, 2055.

[50] *For reviews, see*:

[50a] V. Singh, *Synlett* **2013**, 2641;

[50b] V. Singh , Acc. Chem. Res. 1999, 32, 324.

[51] *For selected recent examples, see*:

[51a] S. N. Good , R. J. Sharpe , J. S. Johnson , J. Am. Chem. Soc. 2017, 139, 12422; 28853553 10.1021/jacs.7b07745 PMC5597490

[51b] R. Sahu , V. Singh , J. Org. Chem. 2017, 82, 6268; 28552002 10.1021/acs.joc.7b00867

[51c] D. B. Jarhad , V. Singh , J. Org. Chem. 2016, 81, 4304; 27149076 10.1021/acs.joc.6b00728

[51d] H. Todoroki , M. Iwatsu , D. Urabe , M. Inoue , J. Org. Chem. 2014, 79, 8835; 25152070 10.1021/jo501666x

[51e] B. Das , S. M. Mobin , V. Singh , Tetrahedron 2014, 70, 4768.

[52] J. Gagnepain , R. Méreau , D. Dejugnac , J.-M. Léger , F. Castet , D. Deffieux , L. Pouységu , S. Quideau , Tetrahedron 2007, 63, 6493.

[53] T. B. Phan , H. Mayr , Can. J. Chem. 2005, 83, 1554.

[54] W. N. Olmstead , Z. Margolin , F. G. Bordwell , J. Org. Chem. 1980, 45, 3295.

[55] R. Schwesinger , H. Schlemper , C. Hasenfratz , J. Willaredt , T. Dambacher , T. Breuer , C. Ottaway , M. Fletschinger , J. Boele , H. Fritz , D. Putzas , H. W. Rotter , F. G. Bordwell , A. V. Satish , G.-Z. Ji , E.-M. Peters , K. Peters , H. G. von Schnering , L. Walz , Liebigs Ann. 1996, 1055.

[56] H. A. Staab , Angew. Chem. 1956, 68, 754.

[57] G. W. Anderson , R. Paul , J. Am. Chem. Soc. 1958, 80, 4423.

[58] Q. Zhang , J. M. Takacs , Org. Lett. 2008, 10, 545. 18189407 10.1021/ol702890s

[59] J.-P. Gesson , M. Mondon , Bioorg. Med. Chem. Lett. 1993, 3, 735.

[60] C. Guzmán , M. Bagga , A. Kaur , J. Westermarck , D. Abankwa , PLoS One 2014, 9, e92444. 24647355 10.1371/journal.pone.0092444 PMC3960247

