# Supplementary material for: Sidechain Diversification of Grandifloracin Allows Identification of Analogues with Enhanced Anti‐Austerity Activity against Human PANC‐1 Pancreatic Cancer Cells
Source: ChemMedChem. 2019 Dec 10;15(1):125–35. doi: 10.1002/cmdc.201900549 (PMC7003952; doi:10.1002/cmdc.201900549)
Supplement: Supplementary file 1 — Supplementary [file CMDC-15-125-s001.pdf]

CHEMMEDCHEM

Supporting Information

© Copyright Wiley-VCH Verlag GmbH & Co. KGaA, 69451 Weinheim, 2020

Sidechain Diversification of Grandifloracin Allows Identification of Analogues with Enhanced Anti-Austerity Activity against Human PANC-1 Pancreatic Cancer Cells

Benjamin E. Alexander, Sijia Sun, Matthew J. Palframan, Gabriele Kociok-Köhn, Dya Fita Dibwe, Shiro Watanabe, Lorenzo Caggiano,* Suresh Awale,* and Simon E. Lewis*© 2019 The Authors. Published by Wiley-VCH Verlag GmbH & Co. KGaA. This is an open access article under the terms of the Creative Commons Attribution License, which permits use, distribution and reproduction in any medium, provided the original work is properly cited.

ELECTRONIC SUPPORTING INFORMATION

Spectra of novel compounds . . . . Page 2

Colony count assay – statistical analysis . . . . . . Page 51

X-ray crystallographic data . . . . Page 52

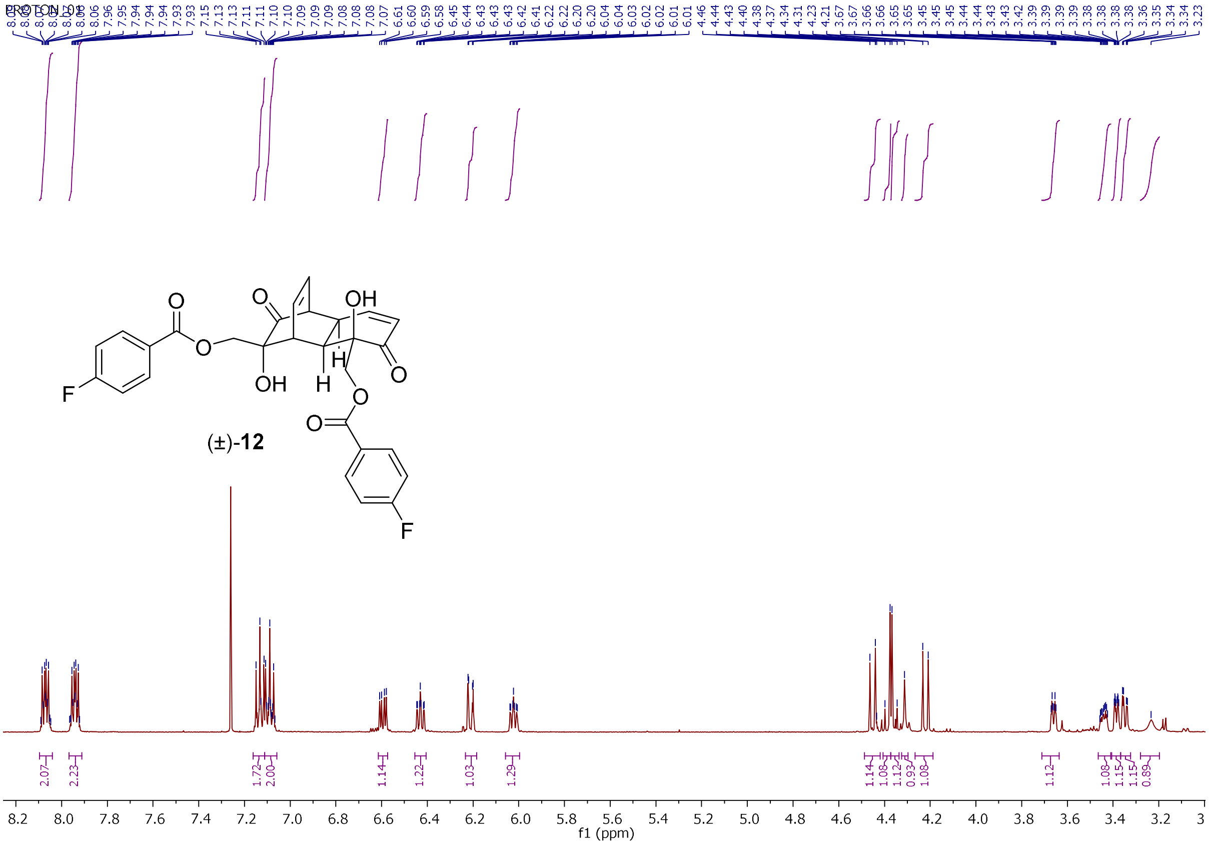
PROTON_01
f1 (ppm)

(±)-**12**

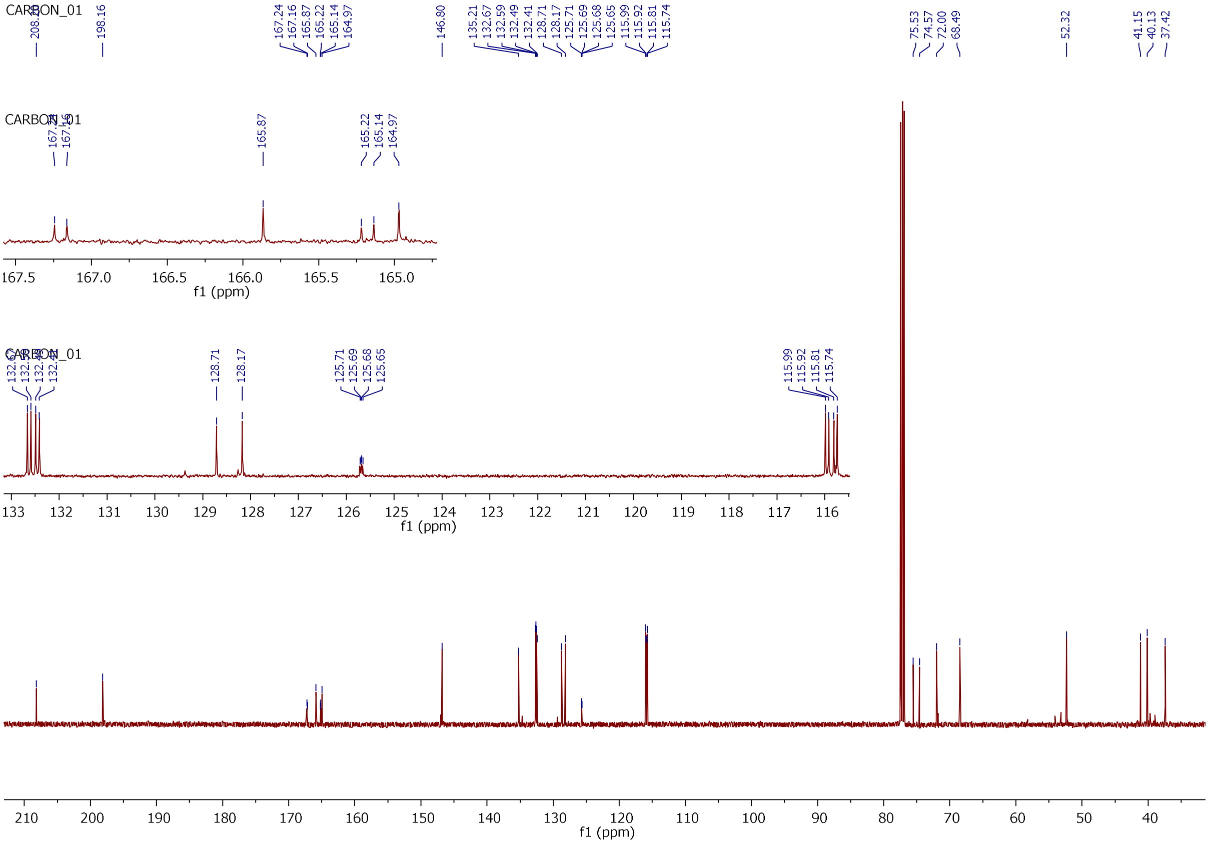
CARBON_01
CARBON_01
f1 (ppm)
CARBON_01
f1 (ppm)
f1 (ppm)

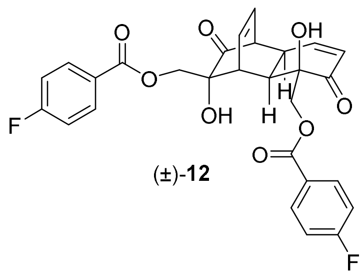
O
O
OH
O
F
OH
H
H
O
O
F

(±)-**12**

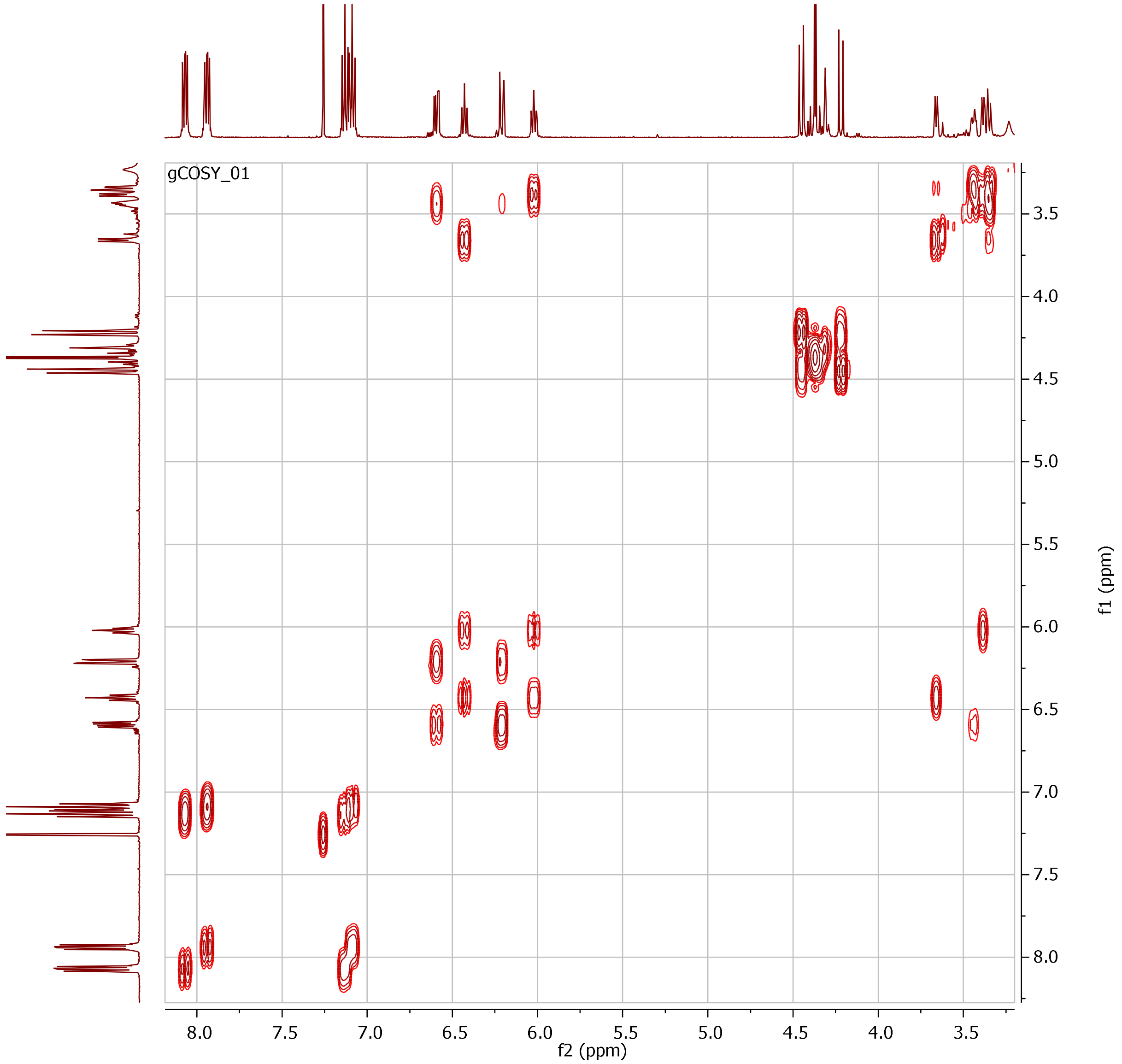
gCOSY_01
f2 (ppm)
f1 (ppm)
8.0
7.5
7.0
6.5
6.0
5.5
5.0
4.5
4.0
3.5

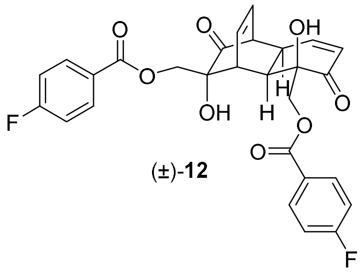

O
O
OH
O
F
OH
H
H
O
O
O
F

(±)-**12**

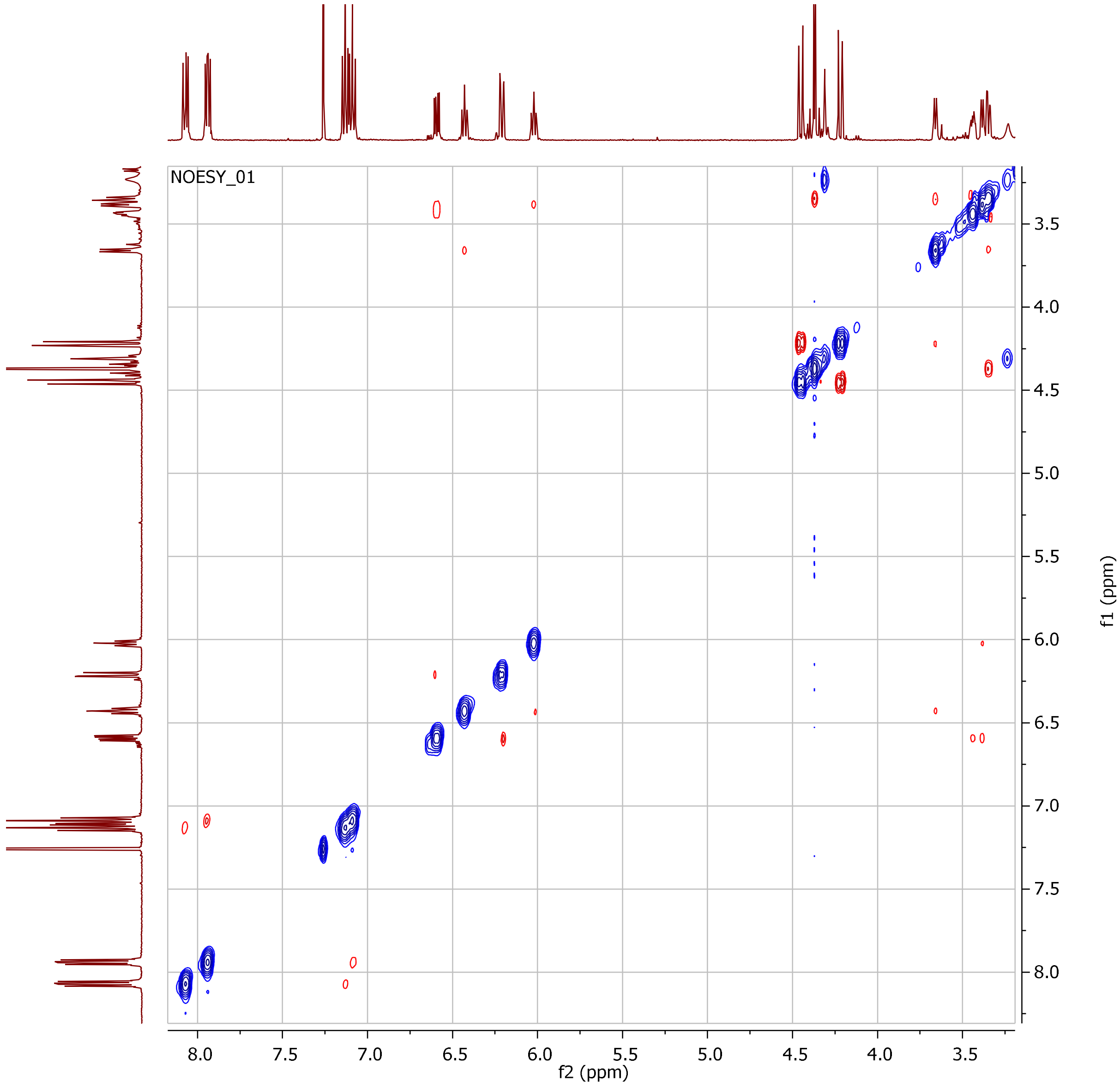

NOESY_01
f1 (ppm)
f2 (ppm)
8.0
7.5
7.0
6.5
6.0
5.5
5.0
4.5
4.0
3.5

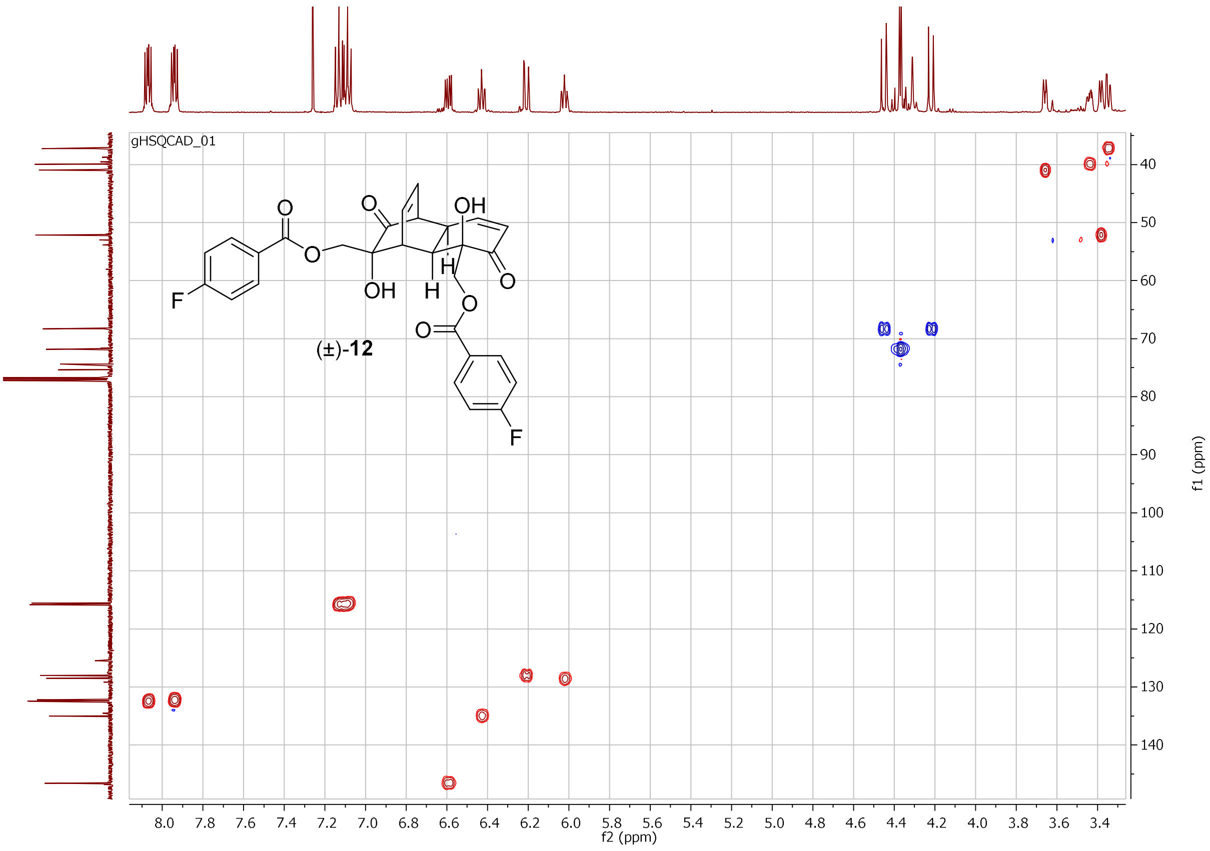
gHSQCAD_01
(±)-12
f2 (ppm)
f1 (ppm)

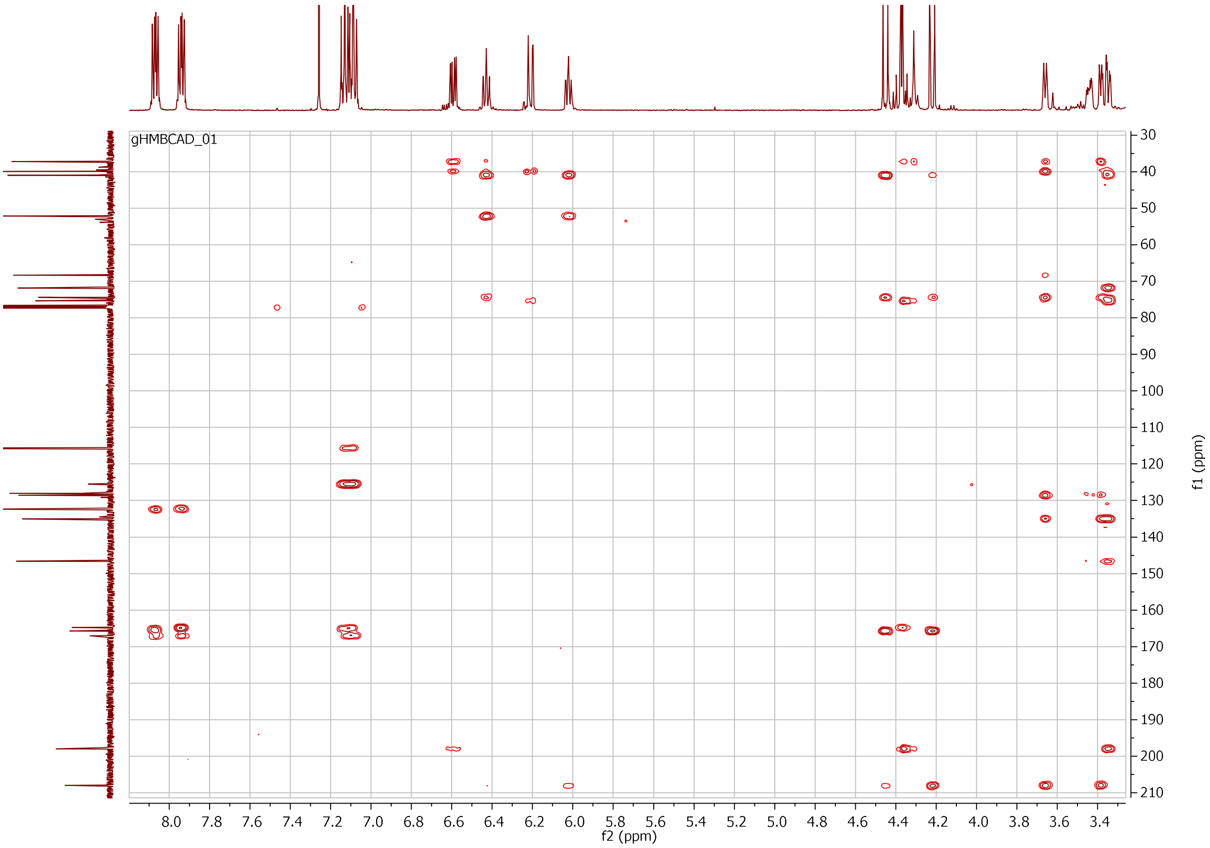
gHMBCAD_01
f2 (ppm)
f1 (ppm)

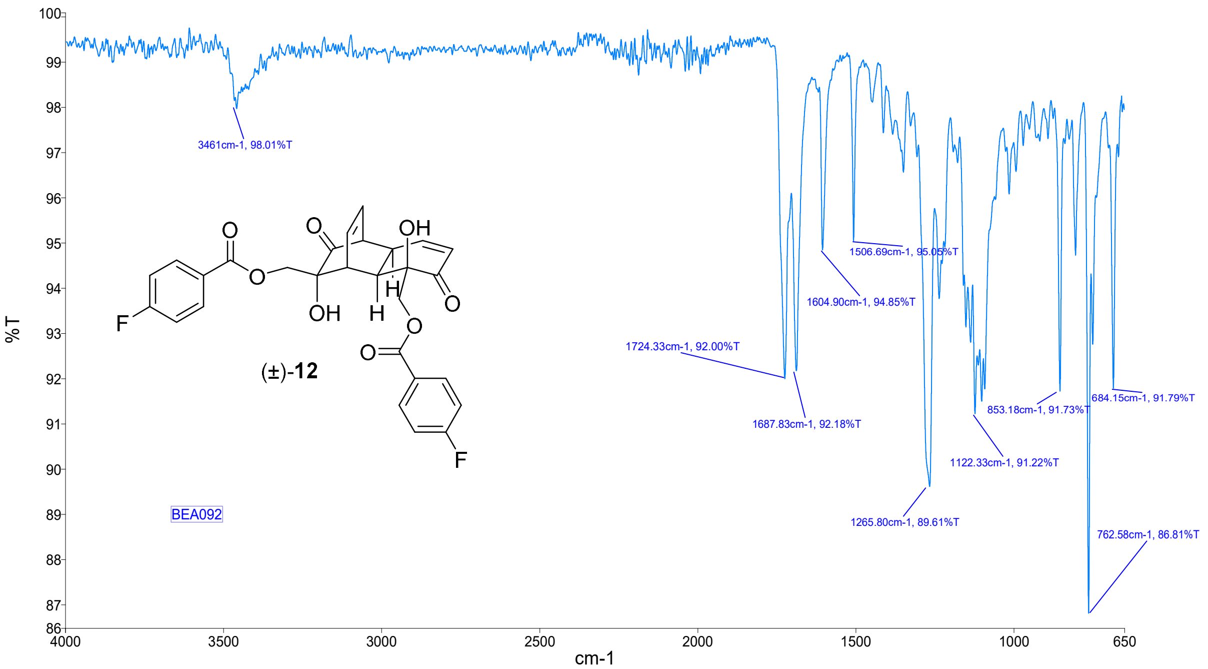

Name	Description
iruser1 6564	Sample 6564 By iruser1 Date Tuesday, February 06 2018

Confirmation of Expected Formula

Sample-ID	ba_sel_I-BEA092	Submitter	bea23 Ben Alexander
Analysis Name	ba_sel_I-BEA092_347654_27_01_52427.d	Supervisor	sl288 Simon Lewis
Method used	Confirm Formula Positive 50to1500 loop inj.m	Acquisition Date	10/05/2016 16:34:21
Ionisation Mode	positive electrospray (ESI)		

+MS, 1.0-1.3min #(24-30), -Spectral Bkgrnd

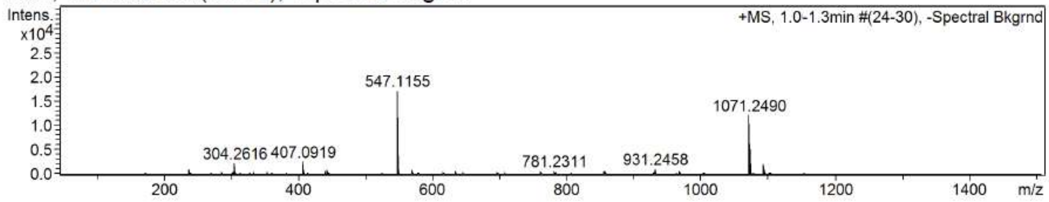

#	m/z	I	I %	Area	S/N
1	304.2616	2263	13.2	147	2710.9
2	407.0919	2556	14.9	213	2420.7
3	547.1155	17195	100.0	1950	2282.7
4	548.1201	4632	26.9	555	607.1
5	931.2458	1109	6.5	116	157.9
6	1071.2490	12062	70.1	2561	1806.2
7	1072.2489	6270	36.5	999	947.6
8	1073.2541	1815	10.6	368	276.8
9	1093.2356	2116	12.3	341	396.3
10	1094.2422	1086	6.3	192	205.7

Generate Molecular Formula Parameters

Charge	Tolerance	SearchRadius	H/C Ratio min.	H/C Ratio max.	Electron Conf.	Nitrogen Rule	sigma limit
positive	10 ppm	0.05 m/z	0	3	both	true	0.05

Expected Formula C28 H22 F2 O8 **Adduct(s):** H, Na

#	meas. m/z	theo. m/z	Err[ppm]	Sigma	Formula
1	547.1155	547.118044	3.60	0.0174	C 28 H 22 F 2 Na 1 O 8

Note: Sigma fits < 0.05 indicates high probability of correct MF, and mass accuracy of 5ppm or better is generally acceptable for publication

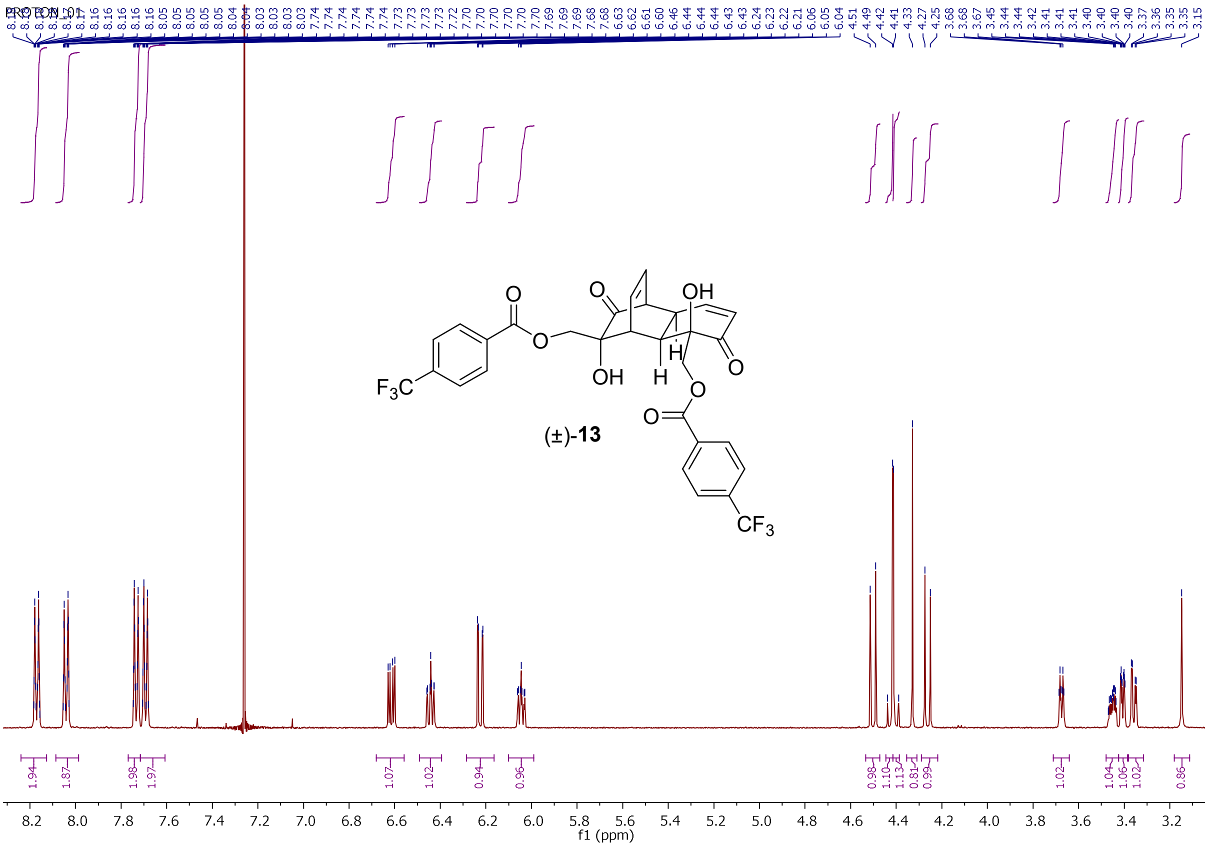
PROTON_01
(±)-13
f1 (ppm)

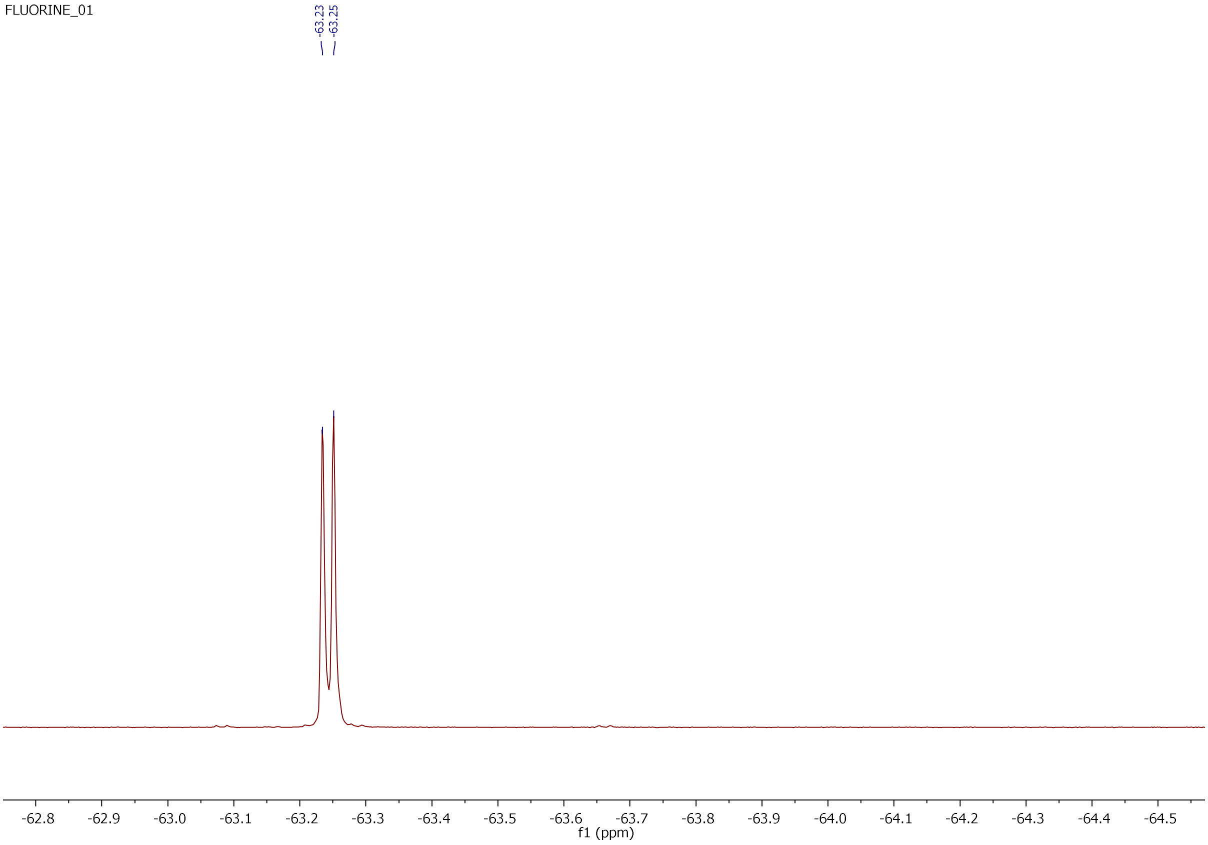
FLUORINE_01
-63.23
-63.25
f1 (ppm)

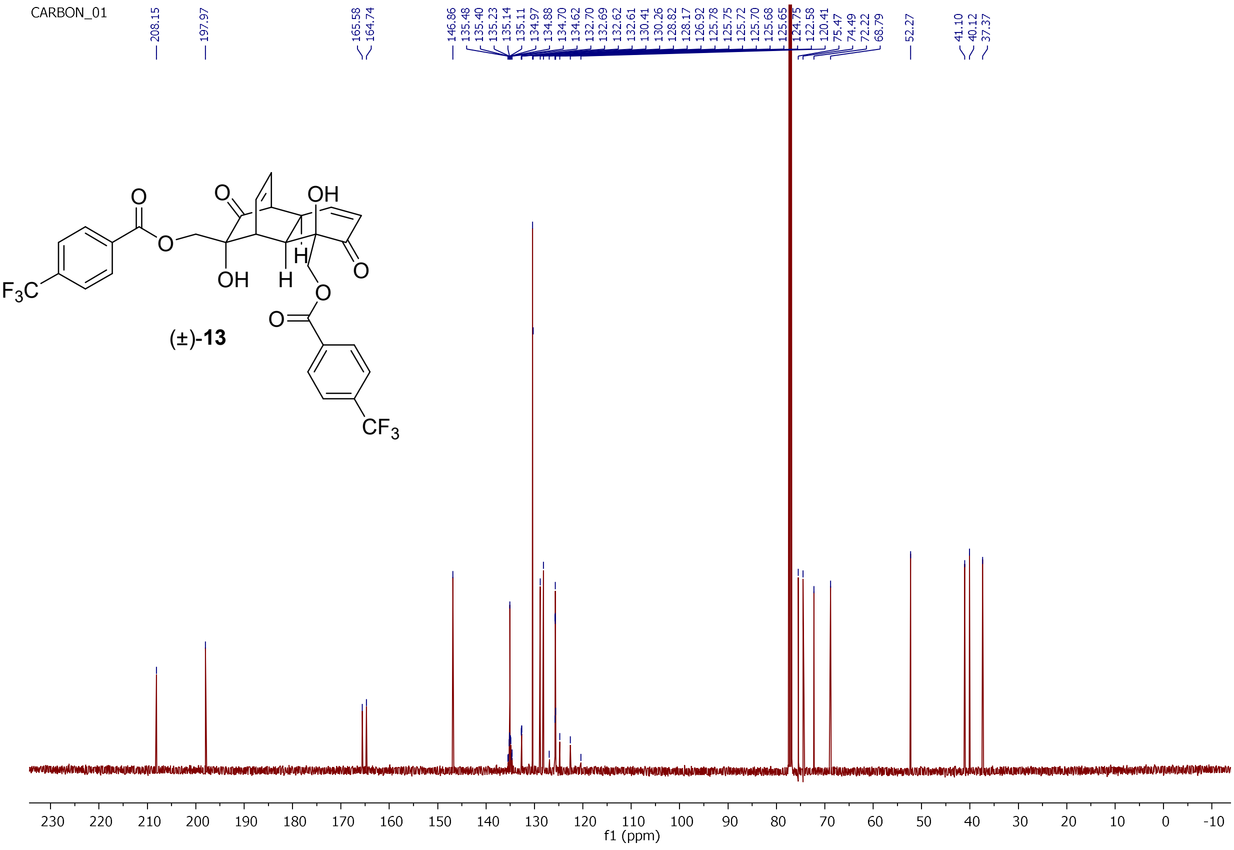
CARBON_01
208.15
197.97
165.58
164.74
146.86
135.48
135.40
135.23
135.14
135.11
134.97
134.88
134.70
134.62
132.70
132.69
132.62
132.61
130.41
130.26
128.82
128.17
126.92
125.78
125.75
125.72
125.70
125.68
125.65
124.75
122.58
120.41
75.47
74.49
72.22
68.79
52.27
41.10
40.12
37.37
f1 (ppm)

(±)-**13**

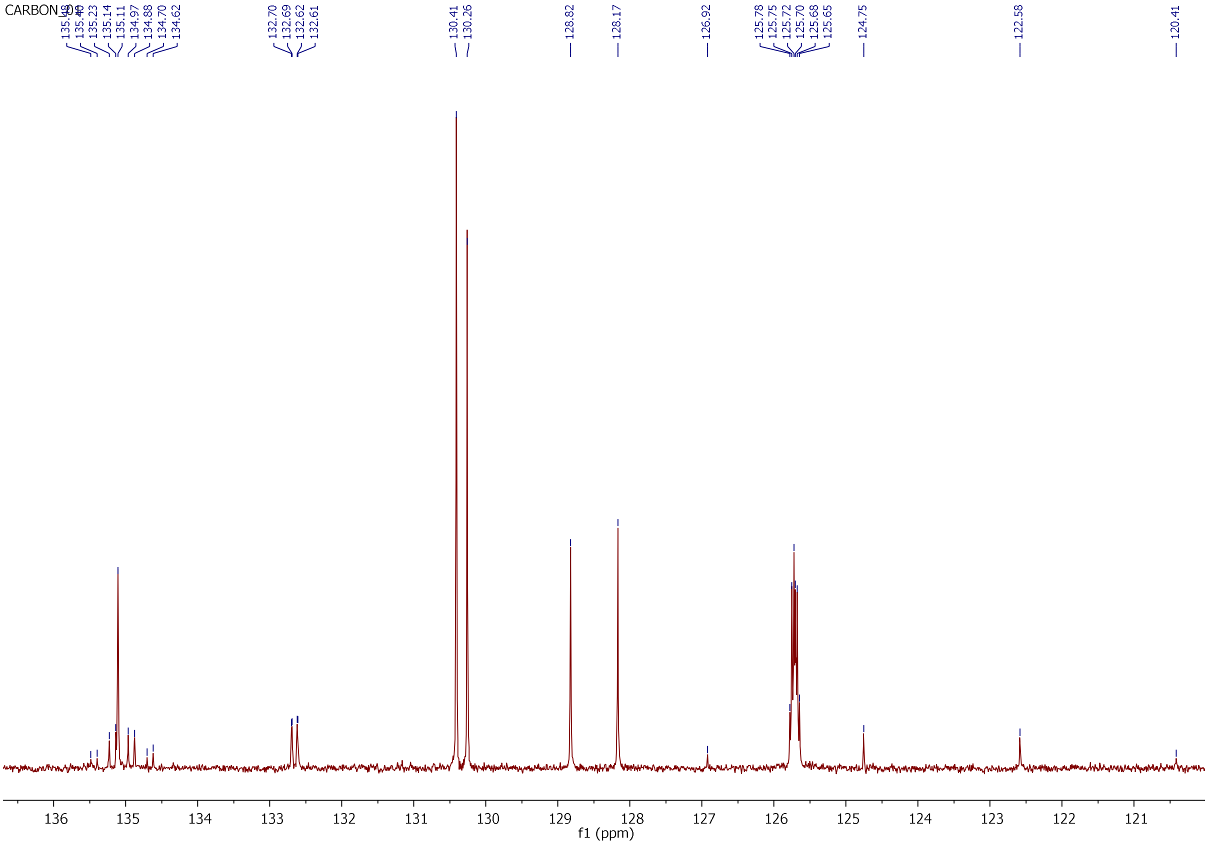
CARBON_01
135.48
135.40
135.23
135.14
135.11
134.97
134.88
134.70
134.62
132.70
132.69
132.62
132.61
130.41
130.26
128.82
128.17
126.92
125.78
125.75
125.72
125.70
125.68
125.65
124.75
122.58
120.41
f1 (ppm)

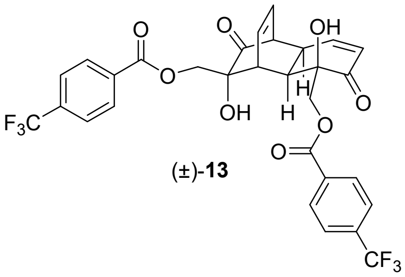

(±)-**13**

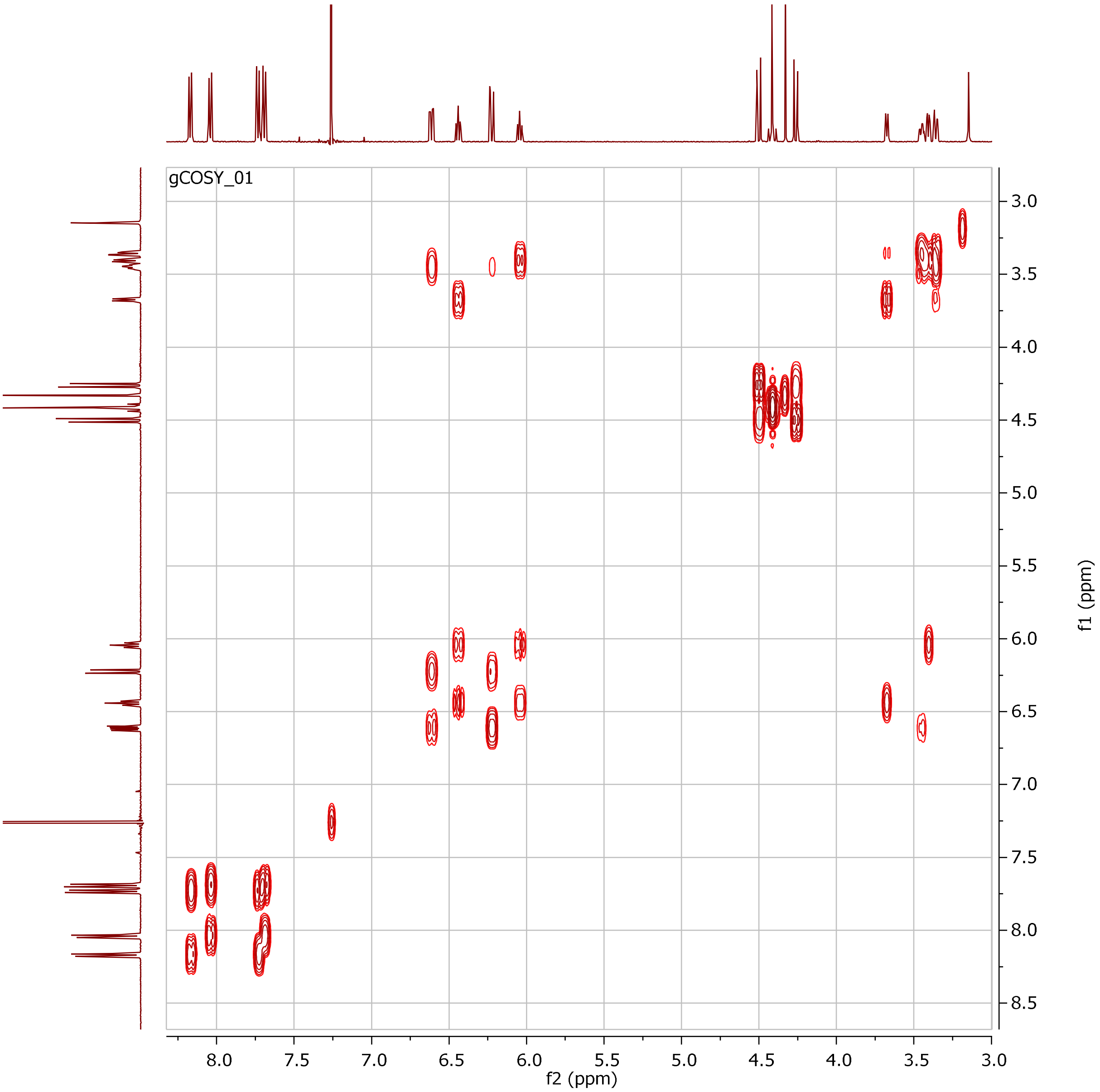

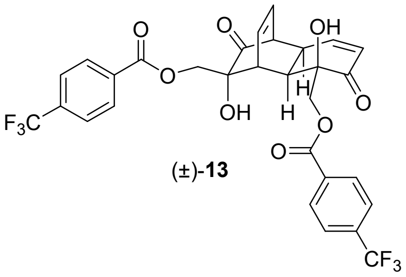
O
O
OH
O
O
F3C
OH
H
H
O
O
CF3

(±)-**13**

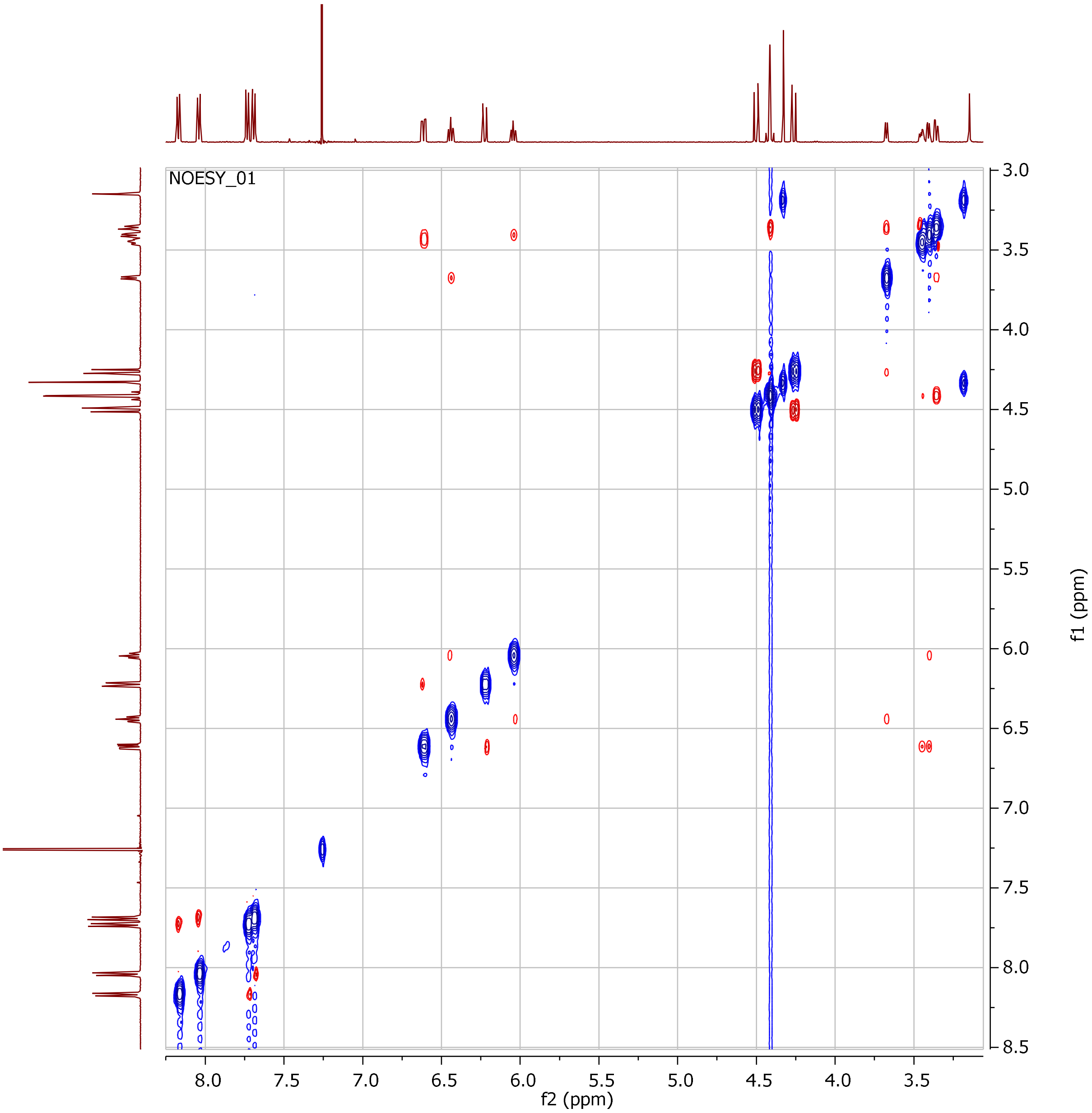
NOESY_01
f2 (ppm)
f1 (ppm)
8.0
7.5
7.0
6.5
6.0
5.5
5.0
4.5
4.0
3.5
3.0
8.5

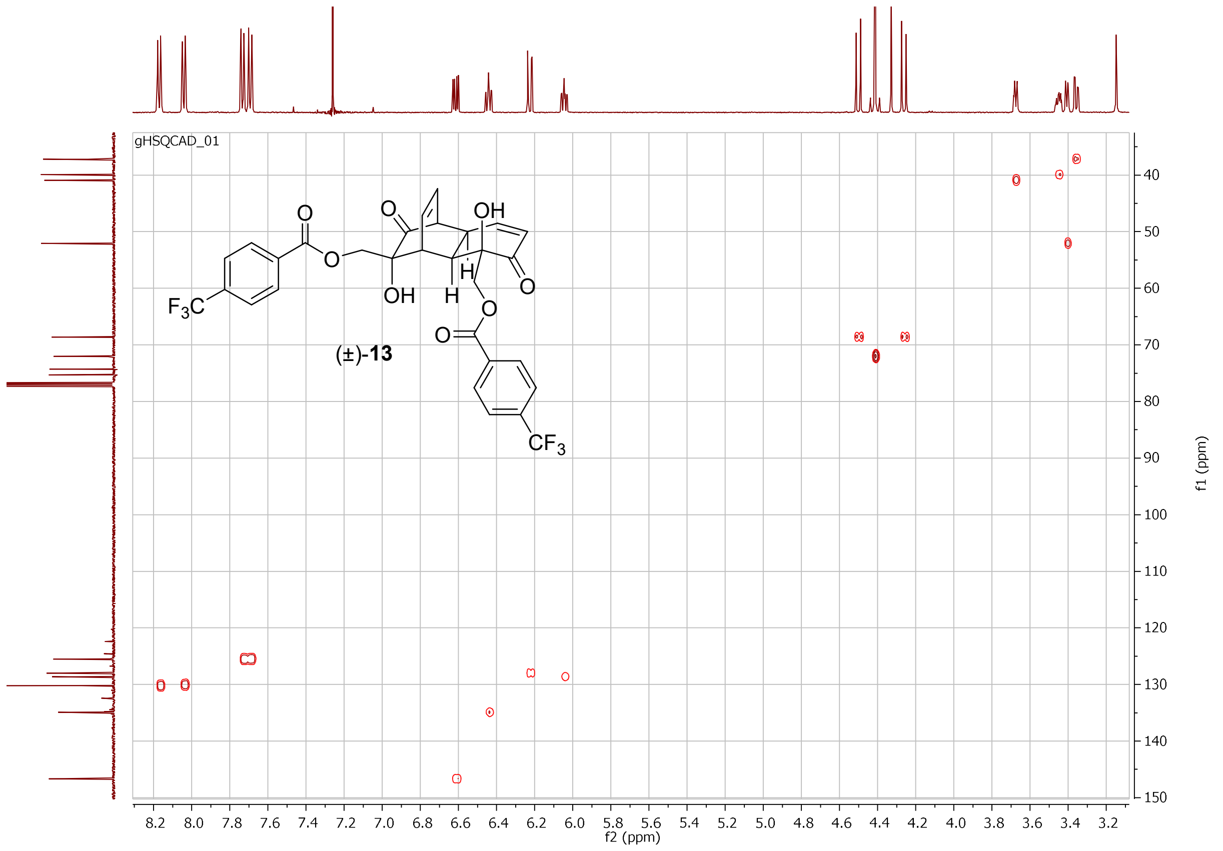
gHSQCAD_01
(±)-13
f2 (ppm)
f1 (ppm)

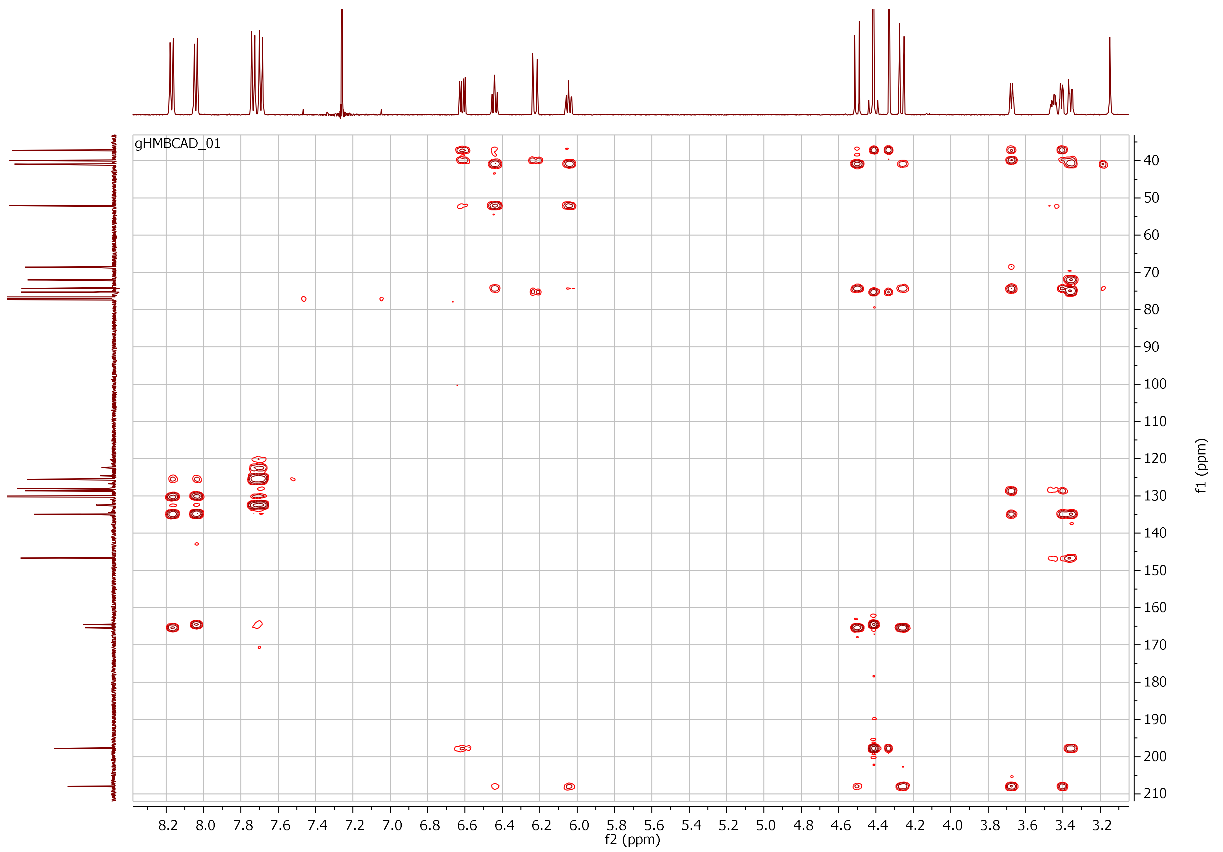
gHMBCAD_01
f2 (ppm)
f1 (ppm)

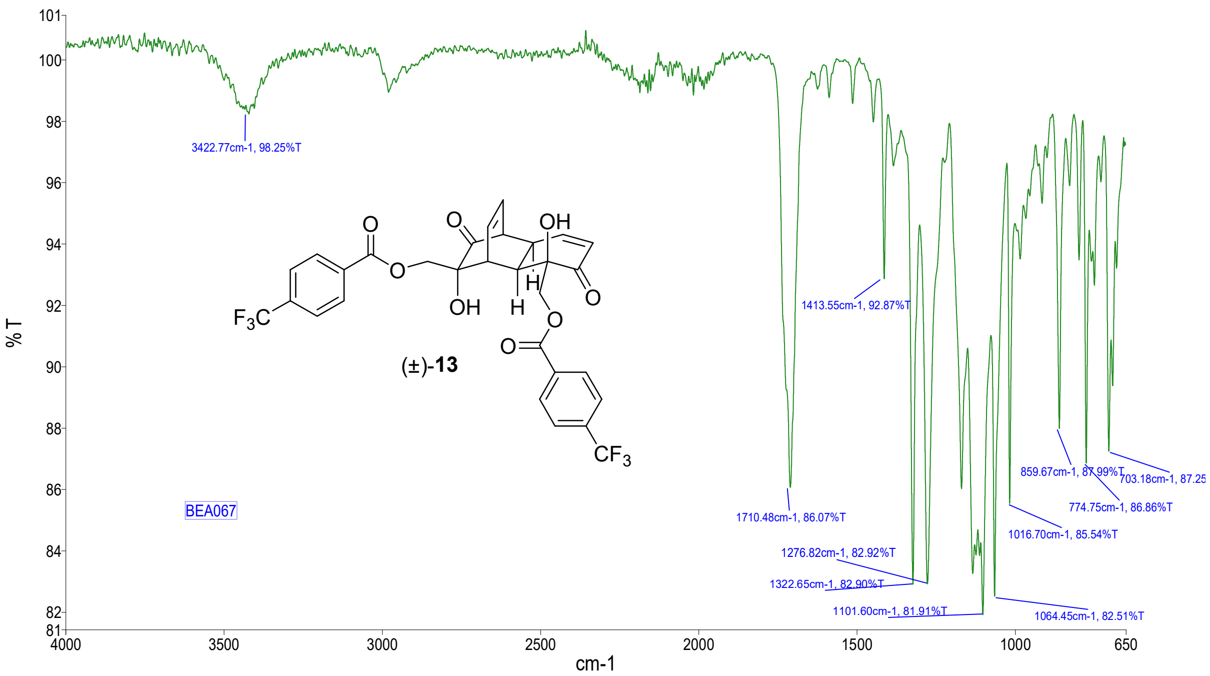

Confirmation of Expected Formula

Sample-ID	ba_sel_I-BEA067	Submitter	Ben Alexander
Analysis Name	ba_sel_I-BEA067_345391_8_01_49940.d	Supervisor	Simon Lewis
Method used	Confirm Formula Positive 50to1500 loop inj.m	Acquisition Date	02/11/2015 15:23:48
Ionisation Mode	positive electrospray (ESI)		

+MS, 1.0-1.3min #(47-60), -Spectral Bkgrnd

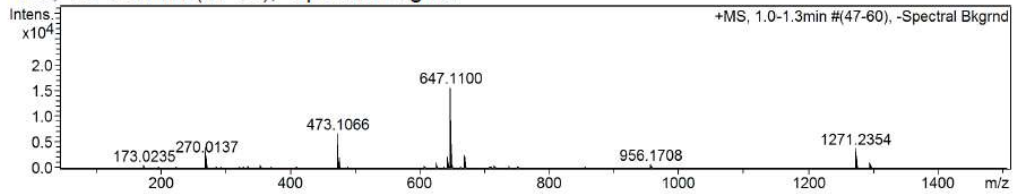

#	m/z	I	I %	Area	S/N
1	270.0137	2332	15.0	25	2460.1
2	473.1066	6690	42.9	176	8096.2
3	475.1073	2094	13.4	79	2509.9
4	625.1301	1196	7.7	167	481.9
5	642.1548	2286	14.7	343	698.9
6	647.1100	15581	100.0	2304	4451.3
7	648.1157	4750	30.5	736	1339.2
8	669.0920	2258	14.5	382	499.8
9	1271.2354	3814	24.5	846	2992.7
10	1272.2464	2396	15.4	548	1900.1

Generate Molecular Formula Parameters

Charge	Tolerance	SearchRadius	H/C Ratio min.	H/C Ratio max.	Electron Conf.	Nitrogen Rule	sigma limit
positive	10 ppm	0.05 m/z	0	3	both	true	0.05

Expected Formula C30 H22 F6 O8 **Adduct(s):** H, Na

#	meas. m/z	theo. m/z	Err[ppm]	Sigma	Formula
1	625.1301	625.129712	-1.50	0.0395	C 30 H 23 F 6 O 8
1	647.1100	647.111657	1.70	0.0163	C 30 H 22 F 6 Na 1 O 8

Note: Sigma fits < 0.05 indicates high probability of correct MF, and mass accuracy of 5ppm or better is generally acceptable for publication

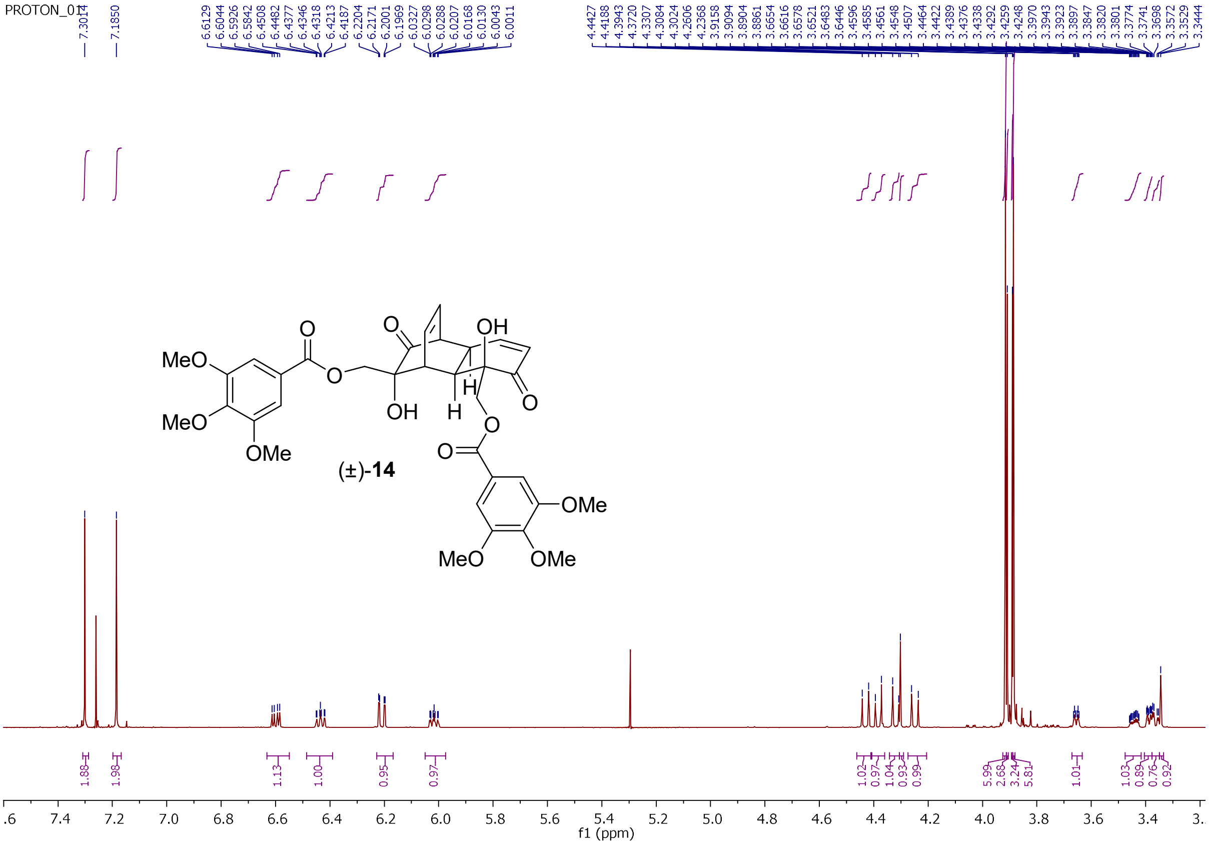
PROTON_01
MeO
MeO
OMe
O
O
OH
OH
H
H
O
O
O
O
OMe
MeO
OMe
(±)-14
f1 (ppm)

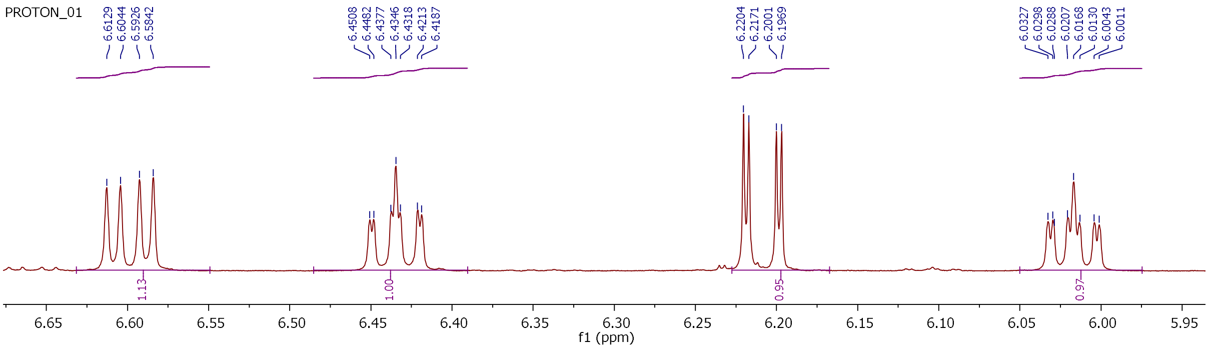
PROTON_01
f1 (ppm)

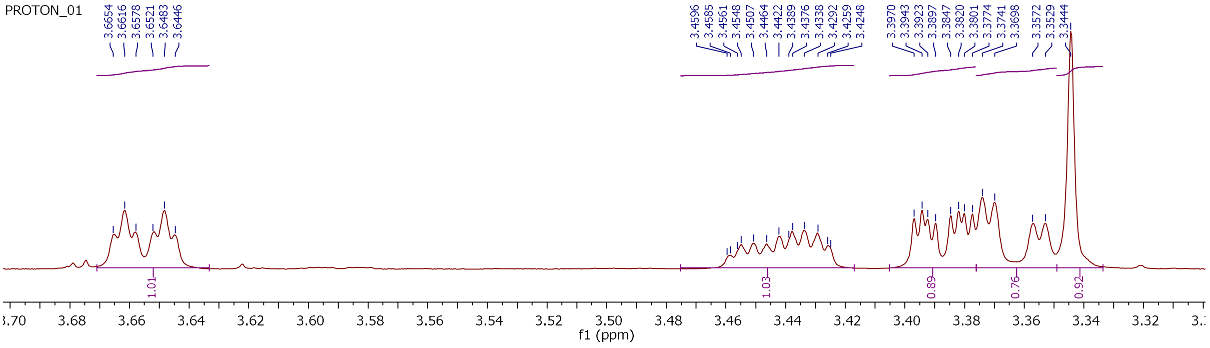
PROTON_01
f1 (ppm)

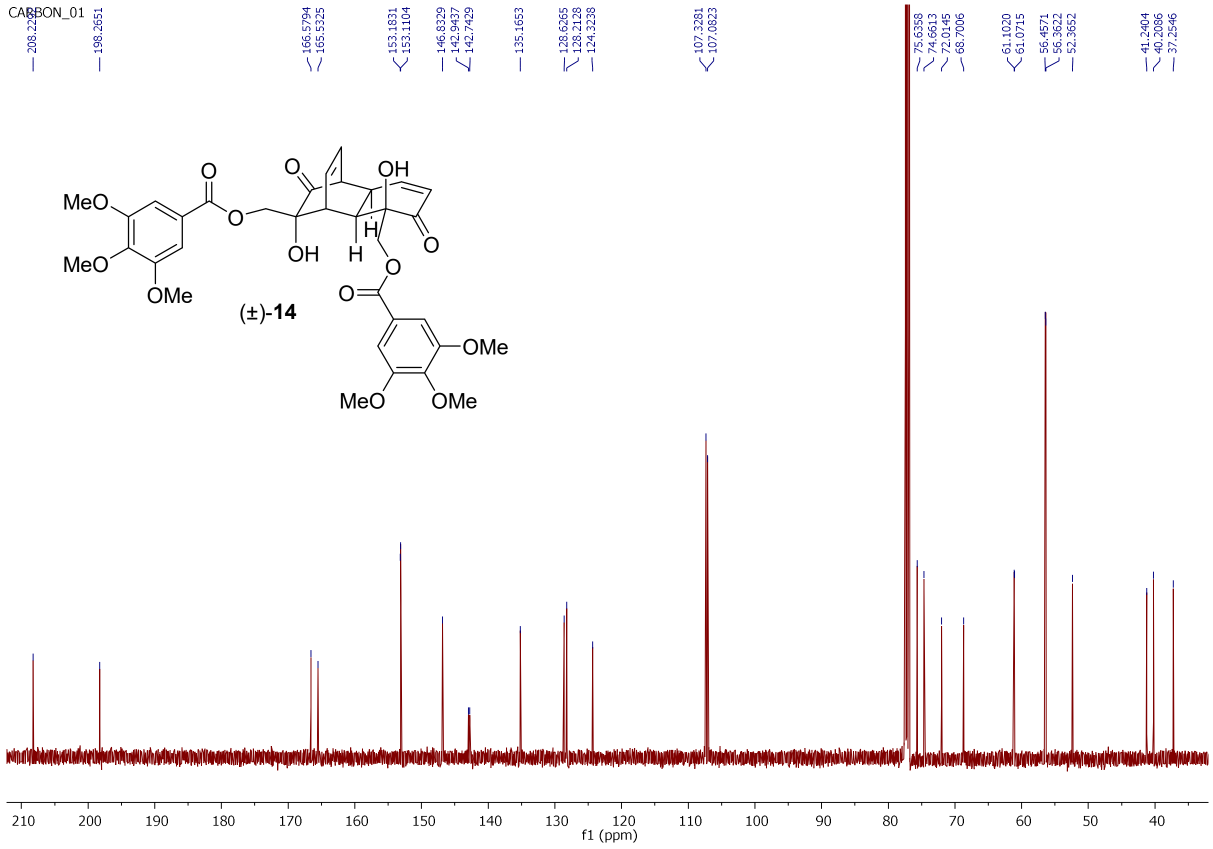
CARBON_01
208.2283
198.2651
166.5794
165.5325
153.1831
153.1104
146.8329
142.9437
142.7429
135.1653
128.6265
128.2128
124.3238
107.3281
107.0823
75.6358
74.6613
72.0145
68.7006
61.1020
61.0715
56.4571
56.3622
52.3652
41.2404
40.2086
37.2546
MeO
MeO
OMe
OH
OH
H
H
O
OMe
MeO
OMe
(±)-14
f1 (ppm)

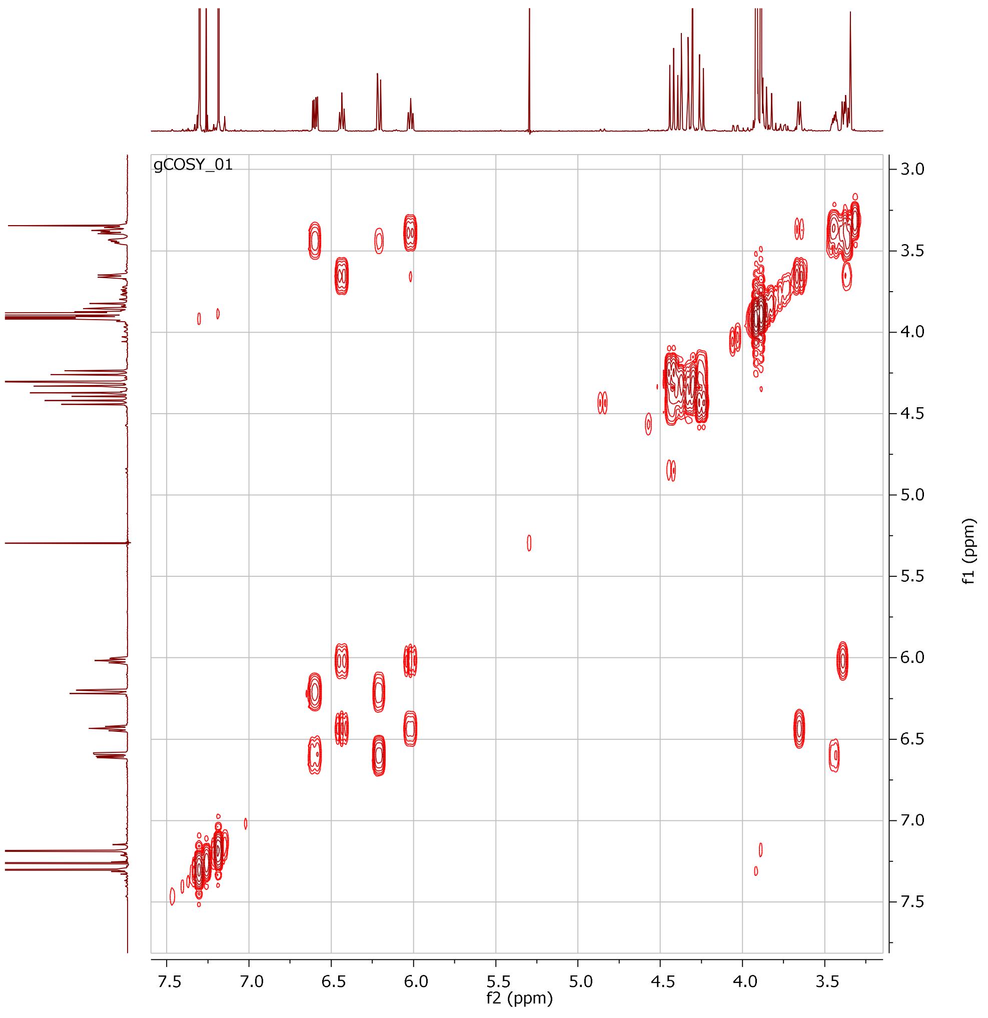
gCOSY_01
f2 (ppm)
f1 (ppm)

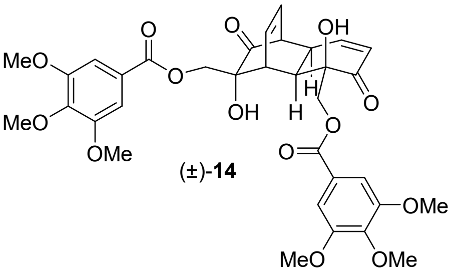
MeO
MeO
OMe
O
O
OH
O
OH
H
H
O
O
OMe
MeO
OMe
O
(±)-**14**

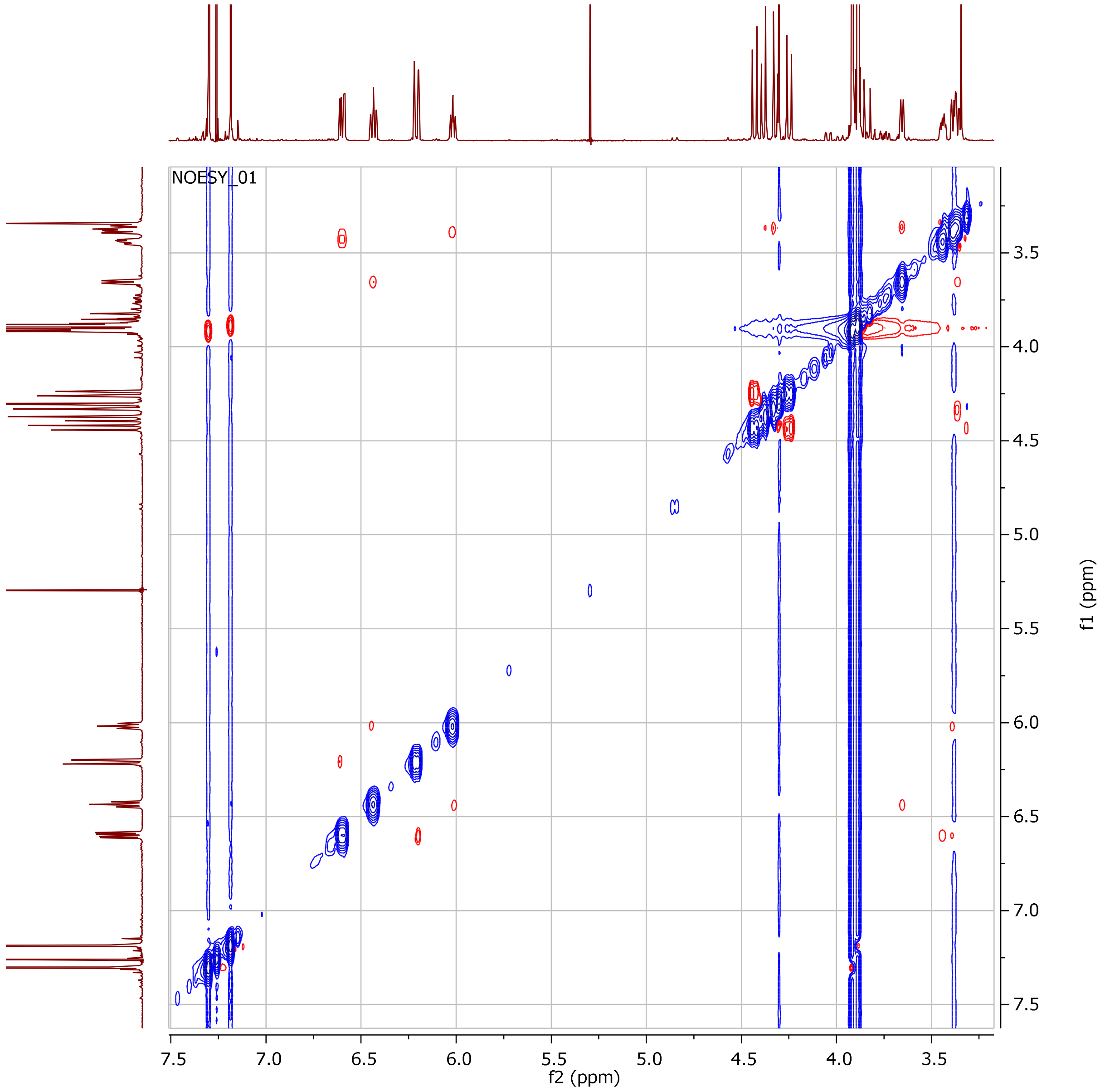
NOESY_01
f2 (ppm)
f1 (ppm)
7.5
7.0
6.5
6.0
5.5
5.0
4.5
4.0
3.5

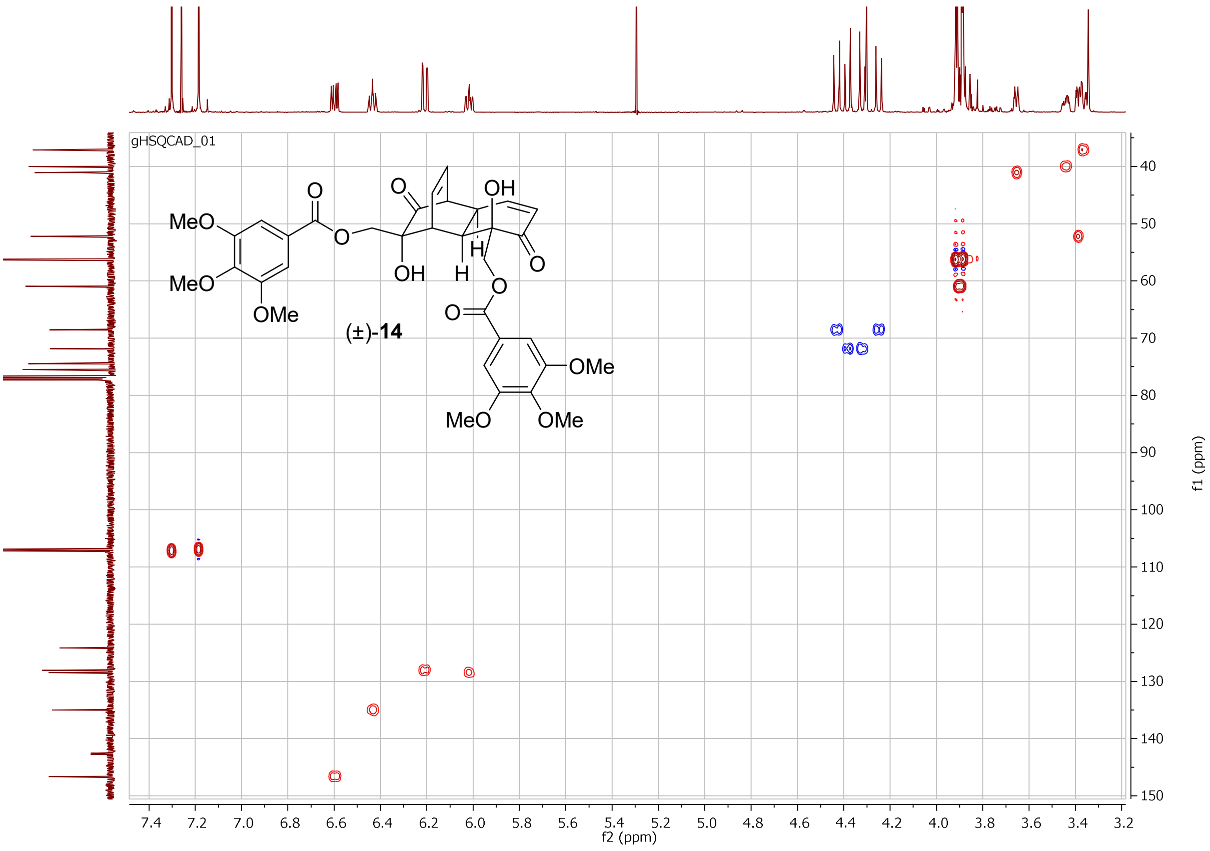
gHSQCAD_01
MeO
MeO
OMe
O
O
OH
OH
H
H
O
O
O
O
OMe
MeO
OMe
(±)-14
f2 (ppm)
f1 (ppm)

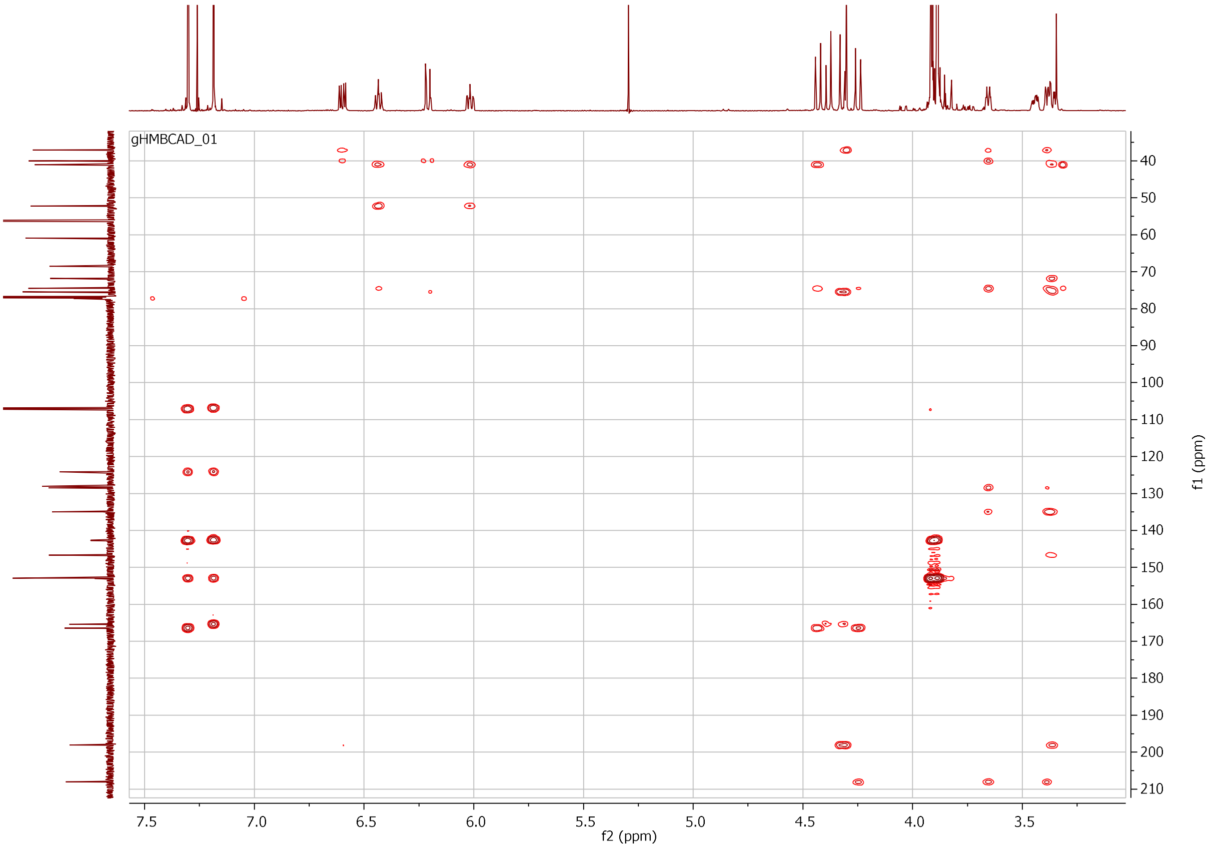
gHMBCAD_01
f2 (ppm)
f1 (ppm)

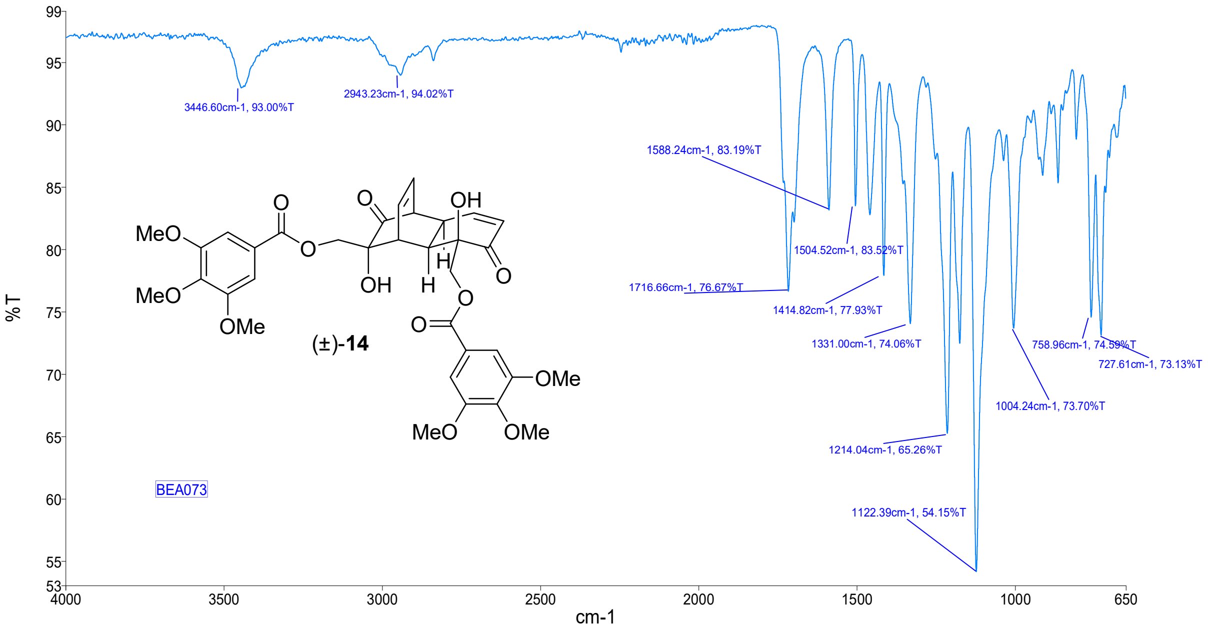

Confirmation of Expected Formula

Sample-ID	ba_sel_I-BEA073	Submitter	bea23 Ben Alexander
Analysis Name	ba_sel_I-BEA073_347653_26_01_52426.d	Supervisor	sl288 Simon Lewis
Method used	Confirm Formula Positive 50to1500 loop inj.m	Acquisition Date	10/05/2016 16:30:56
Ionisation Mode	positive electrospray (ESI)		

+MS, 1.0-1.3min #(24-30), -Spectral Bkgrnd

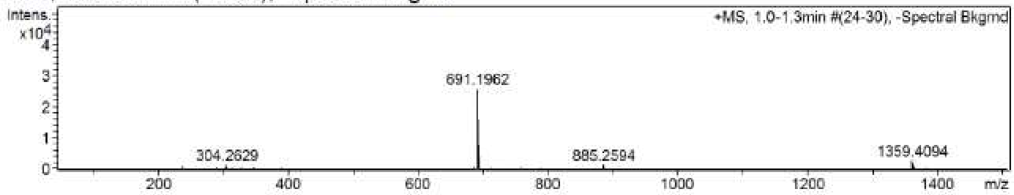

#	m/z	I	I %	Area	S/N
1	236.1107	958	3.7	10	2346.9
2	304.2629	1579	6.1	109	2029.8
3	691.1962	25983	100.0	2736	3203.9
4	692.2067	7858	30.2	881	976.1
5	693.2067	1491	5.7	168	186.5
6	759.1886	750	2.9	85	180.6
7	885.2594	1471	5.7	211	627.7
8	1359.4094	2817	10.8	627	987.1
9	1360.4137	2057	7.9	433	727.8
10	1361.4134	734	2.8	171	262.2

Generate Molecular Formula Parameters

Charge	Tolerance	SearchRadius	H/C Ratio min.	H/C Ratio max.	Electron Conf.	Nitrogen Rule	sigma limit
positive	10 ppm	0.05 m/z	0	3	both	true	0.05

Expected Formula C34 H36 O14 **Adduct(s):** H, Na

#	meas. m/z	theo. m/z	Err[ppm]	Sigma	Formula
1	691.1962	691.200276	5.10	0.0429	C 34 H 36 Na 1 O 14

Note: Sigma fits < 0.05 indicates high probability of correct MF, and mass accuracy of 5ppm or better is generally acceptable for publication

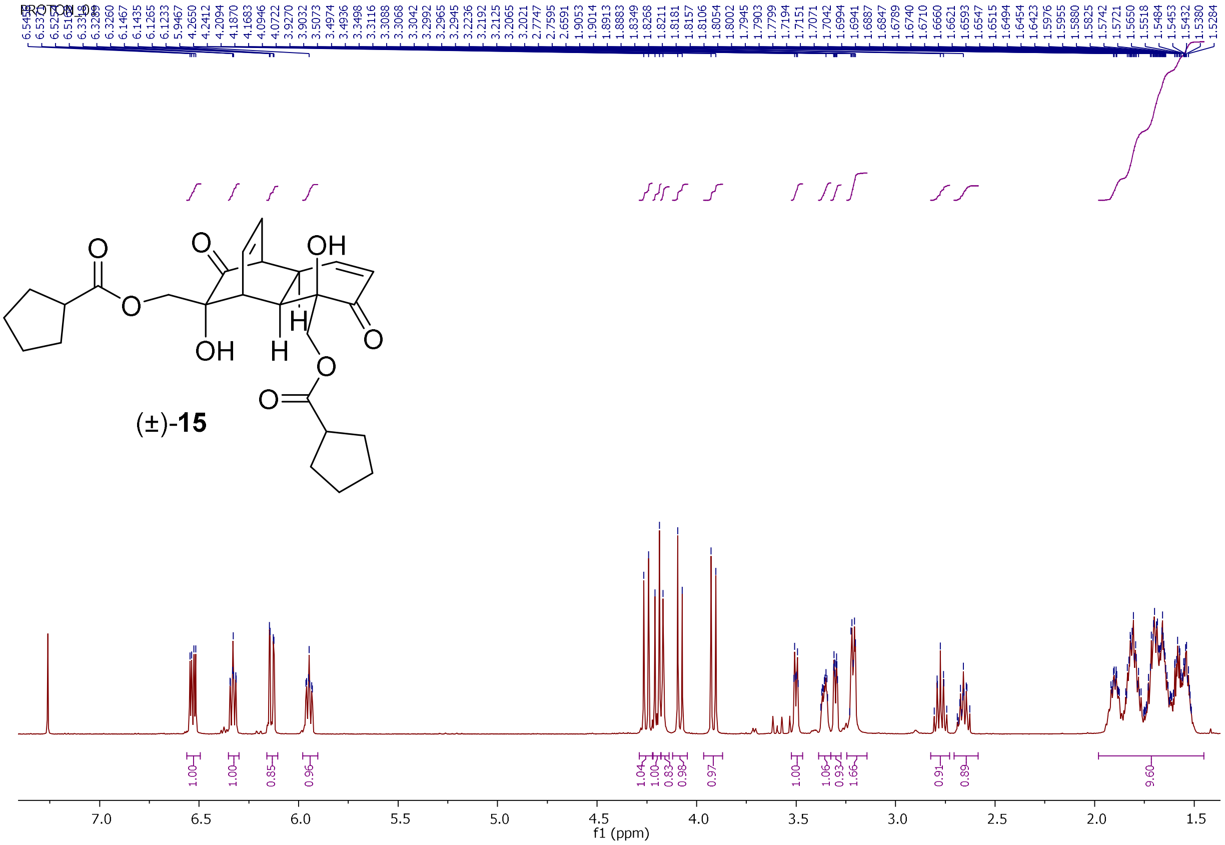
(±)-15
PROTON_01
6.5459
6.5370
6.5250
6.5162
6.3318
6.3289
6.3260
6.1467
6.1435
6.1265
6.1233
5.9467
4.2650
4.2412
4.2094
4.1870
4.1683
4.0946
4.0722
3.9270
3.9032
3.5073
3.4974
3.4936
3.3498
3.3116
3.3088
3.3068
3.3042
3.2992
3.2965
3.2945
3.2236
3.2192
3.2125
3.2065
3.2021
2.7747
2.7595
2.6591
1.9053
1.9014
1.8913
1.8883
1.8349
1.8268
1.8211
1.8181
1.8157
1.8106
1.8054
1.8002
1.7945
1.7903
1.7799
1.7194
1.7151
1.7071
1.7042
1.6994
1.6941
1.6887
1.6847
1.6789
1.6740
1.6710
1.6660
1.6621
1.6593
1.6547
1.6515
1.6494
1.6454
1.6423
1.5976
1.5955
1.5880
1.5825
1.5742
1.5721
1.5650
1.5518
1.5484
1.5453
1.5432
1.5380
1.5284
1.00
1.00
0.85
0.96
1.04
1.00
0.83
0.98
0.97
1.00
1.06
0.93
1.66
0.91
0.89
9.60
7.0
6.5
6.0
5.5
5.0
4.5
4.0
3.5
3.0
2.5
2.0
1.5
f1 (ppm)

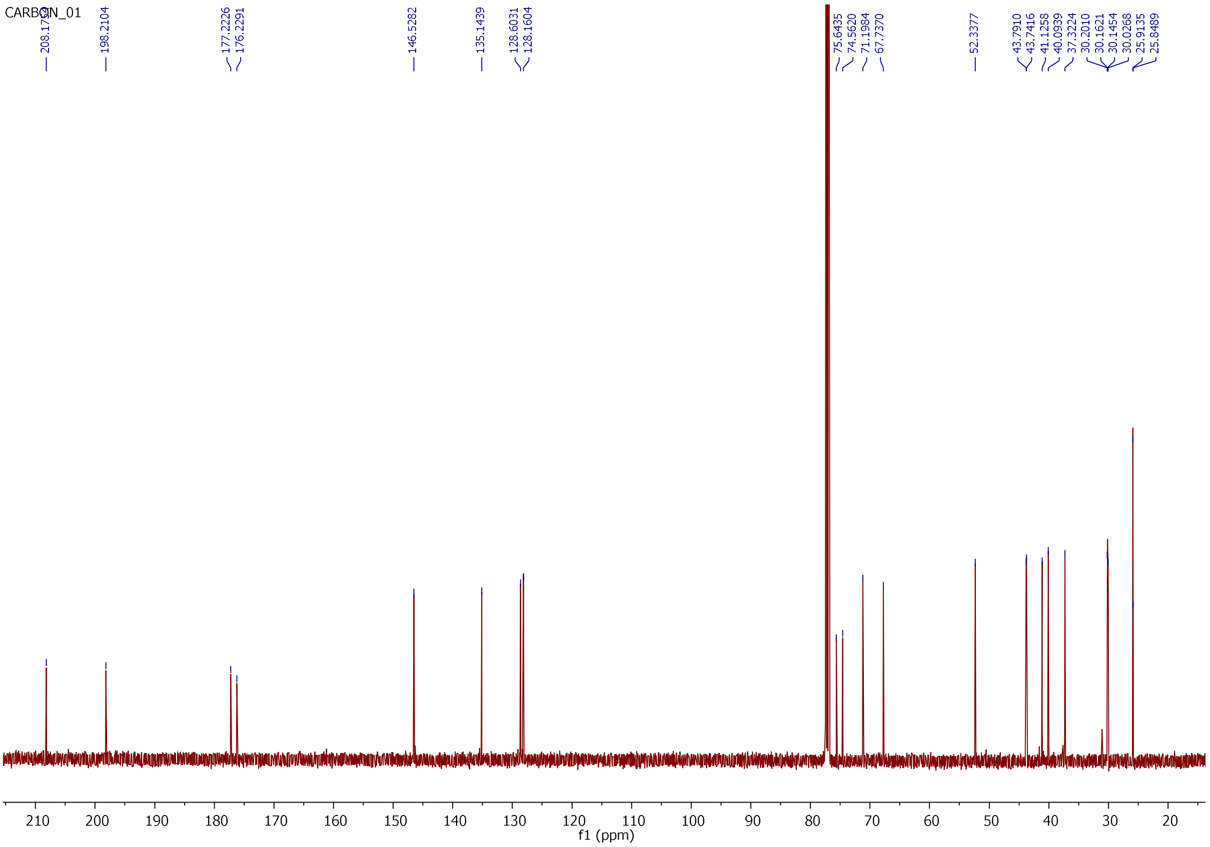
CARBON_01
208.1747
198.2104
177.2226
176.2291
146.5282
135.1439
128.6031
128.1604
75.6435
74.5620
71.1984
67.7370
52.3377
43.7910
43.7416
41.1258
40.0939
37.3224
30.2010
30.1621
30.1454
30.0268
25.9135
25.8469
210
200
190
180
170
160
150
140
130
120
110
100
90
80
70
60
50
40
30
20
f1 (ppm)

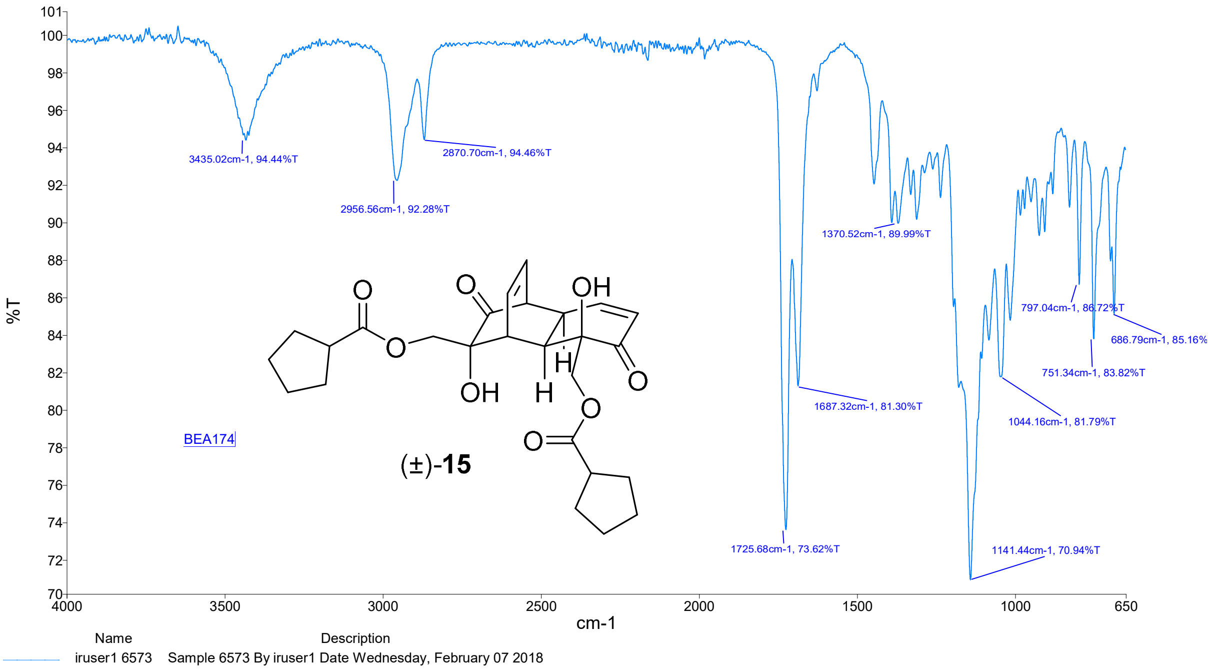

Confirmation of Expected Formula

Sample-ID	ba_sel_BEA174 D	Submitter	bea23 Ben Alexander
Analysis Name	ba_sel_BEA174 D_356386_73_01_62459.d	Supervisor	sl288 Simon Lewis
Method used	Confirm Formula Positive 50to500 loop inj.m	Acquisition Date	22/03/2018 15:21:58
Ionisation Mode	positive electrospray (ESI)		

+MS, 1.0-1.3min #(119-154), -Spectral Bkgrnd

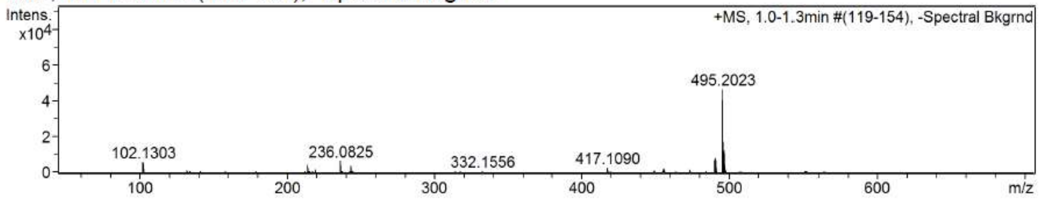

#	m/z	I	I %	Area	S/N
1	102.1303	6004	13.0	122	8348.2
2	214.0936	4297	9.3	112	755.8
3	236.0825	6578	14.2	73	964.2
4	243.1375	3851	8.3	155	536.6
5	417.1090	2708	5.9	181	563.0
6	455.2114	2317	5.0	167	334.7
7	490.2460	8326	18.0	771	633.9
8	495.2023	46206	100.0	4387	3257.0
9	496.2054	12554	27.2	1185	871.8
10	497.2085	2179	4.7	190	149.1

Generate Molecular Formula Parameters

Charge	Tolerance	SearchRadius	H/C Ratio min.	H/C Ratio max.	Electron Conf.	Nitrogen Rule	sigma limit
positive	25 ppm	0.05 m/z	0	3	both	true	0.05

Expected Formula C26 H32 O8 **Adduct(s):** H, Na

#	meas. m/z	theo. m/z	Err[ppm]	Sigma	Formula
1	495.2023	495.1989	6.80	0.0078	C 26 H 32 Na 1 O 8

Note: Sigma fits < 0.05 indicates high probability of correct MF.

For formula confirmation the mass error / accuracy at 200 Da should be better than 25 ppm, for 500 Da better than 10 ppm and for 1000 Da better than 5 ppm

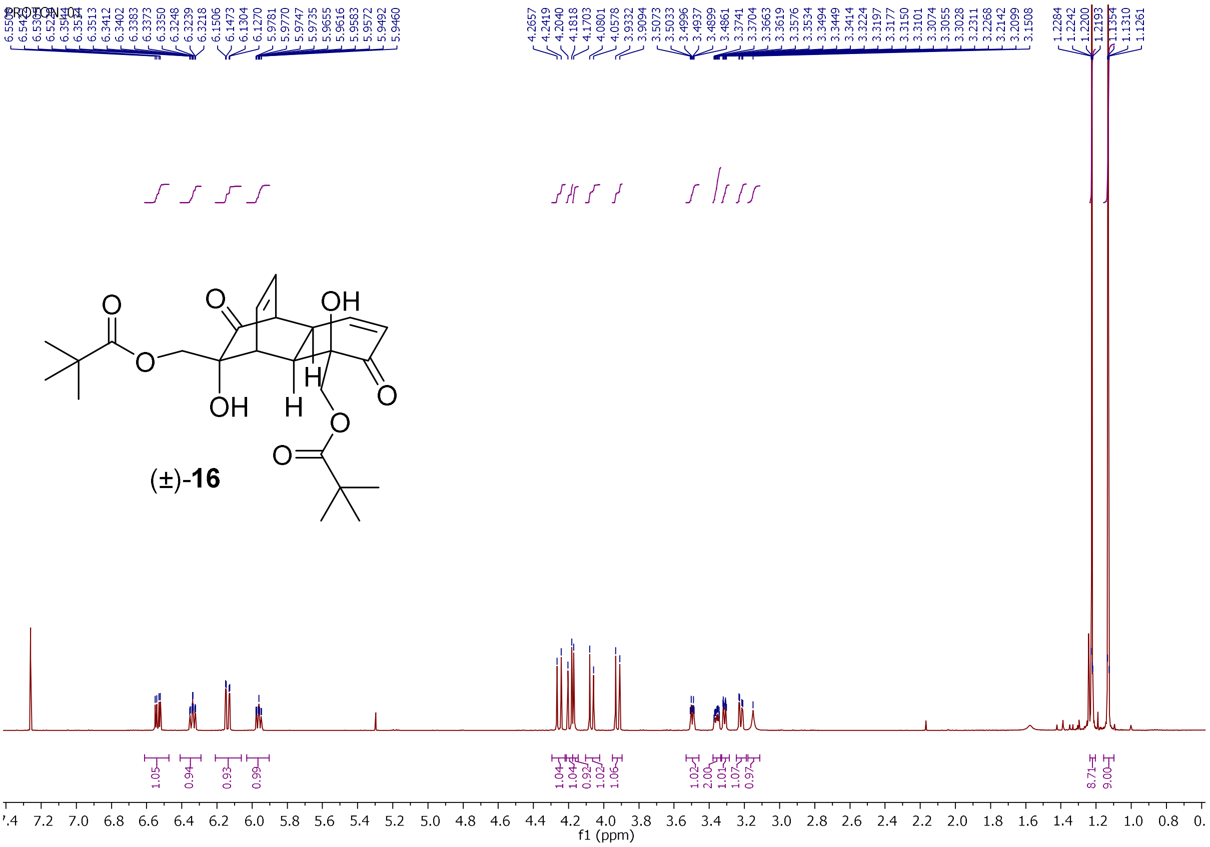
PROTON_01
6.5504
6.5423
6.5305
6.5225
6.3544
6.3514
6.3513
6.3412
6.3402
6.3383
6.3373
6.3350
6.3248
6.3239
6.3218
6.1506
6.1473
6.1304
6.1270
5.9781
5.9770
5.9747
5.9735
5.9655
5.9616
5.9583
5.9572
5.9492
5.9460
4.2657
4.2419
4.2040
4.1818
4.1703
4.0801
4.0578
3.9332
3.9094
3.5073
3.5033
3.4996
3.4937
3.4899
3.4861
3.3741
3.3704
3.3663
3.3619
3.3576
3.3534
3.3494
3.3449
3.3414
3.3224
3.3197
3.3177
3.3150
3.3101
3.3074
3.3055
3.3028
3.2311
3.2268
3.2142
3.2099
3.1508
1.2284
1.2242
1.2200
1.2193
1.1354
1.1310
1.1261
(±)-16
1.05
0.94
0.93
0.99
1.04
1.04
0.92
1.02
1.06
1.02
2.00
1.01
1.07
0.97
8.71
9.00
f1 (ppm)

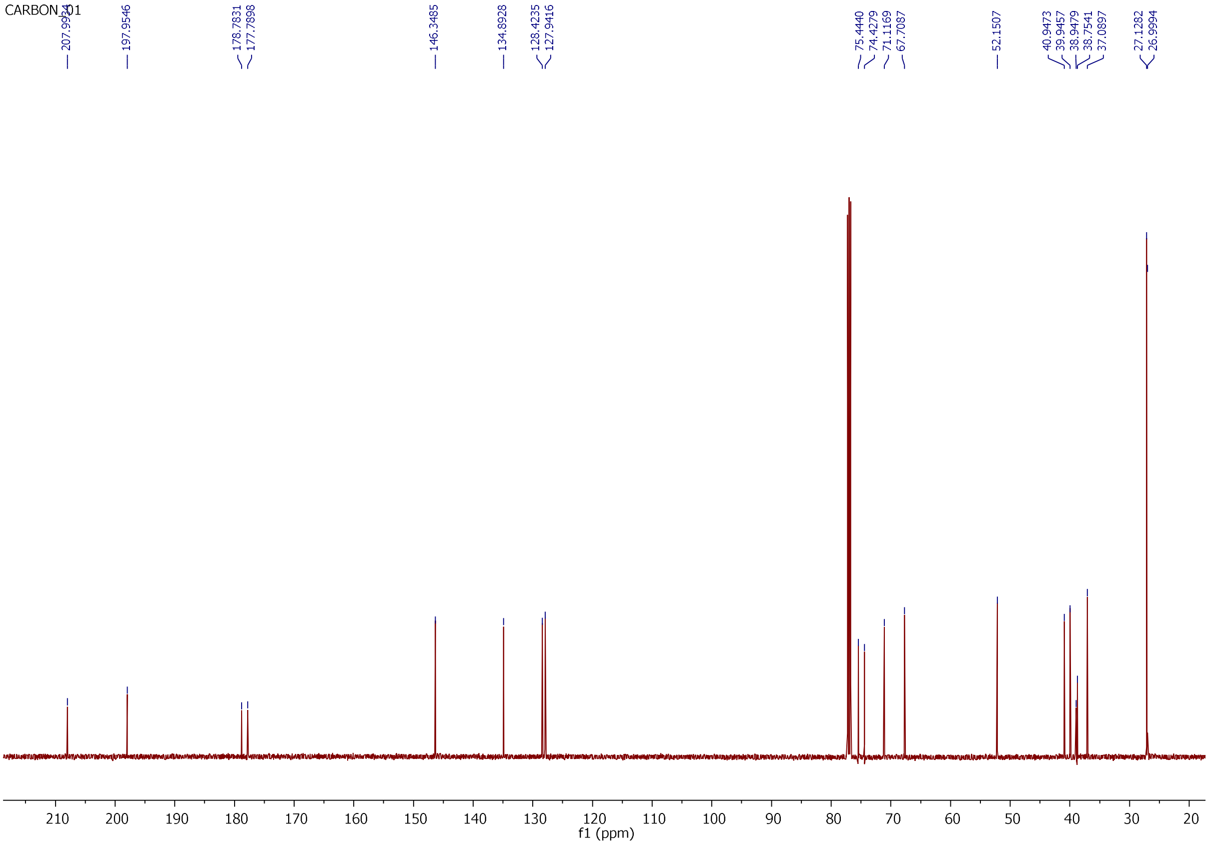
CARBON_01
207.9924
197.9546
178.7831
177.7898
146.3485
134.8928
128.4235
127.9416
75.4440
74.4279
71.1169
67.7087
52.1507
40.9473
39.9457
38.9479
38.7541
37.0897
27.1282
26.9994
f1 (ppm)

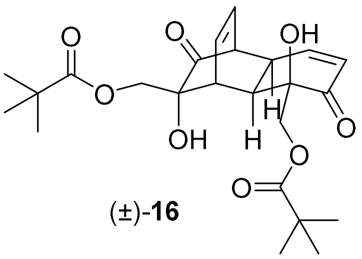

(±)-**16**

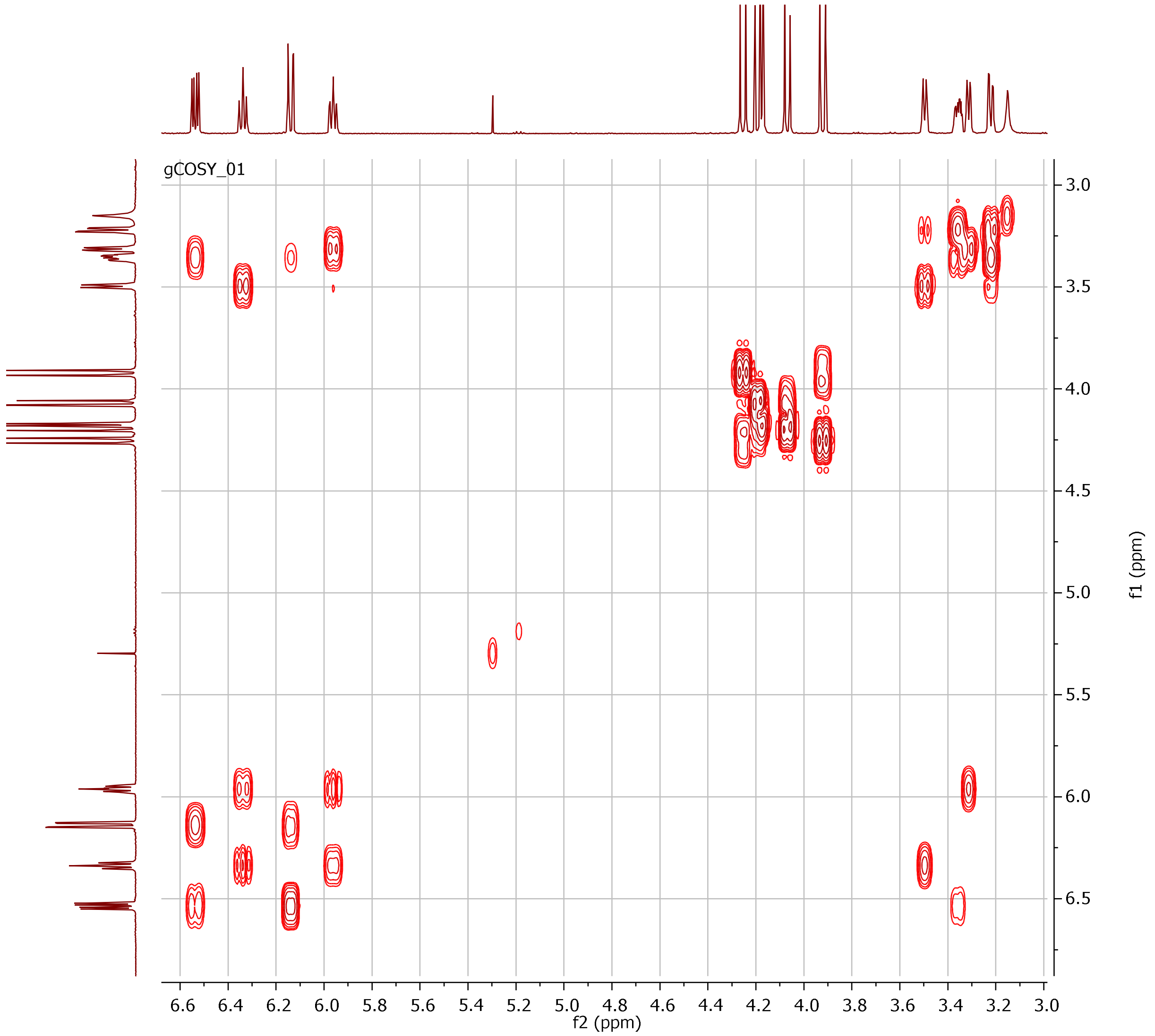
gCOSY_01
6.6
6.4
6.2
6.0
5.8
5.6
5.4
5.2
5.0
4.8
4.6
4.4
4.2
4.0
3.8
3.6
3.4
3.2
3.0
f2 (ppm)
3.0
3.5
4.0
4.5
5.0
5.5
6.0
6.5
f1 (ppm)

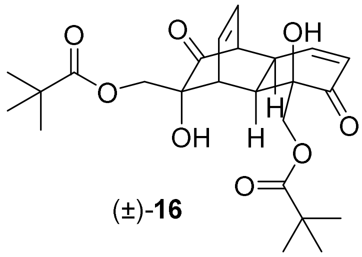

(±)-**16**

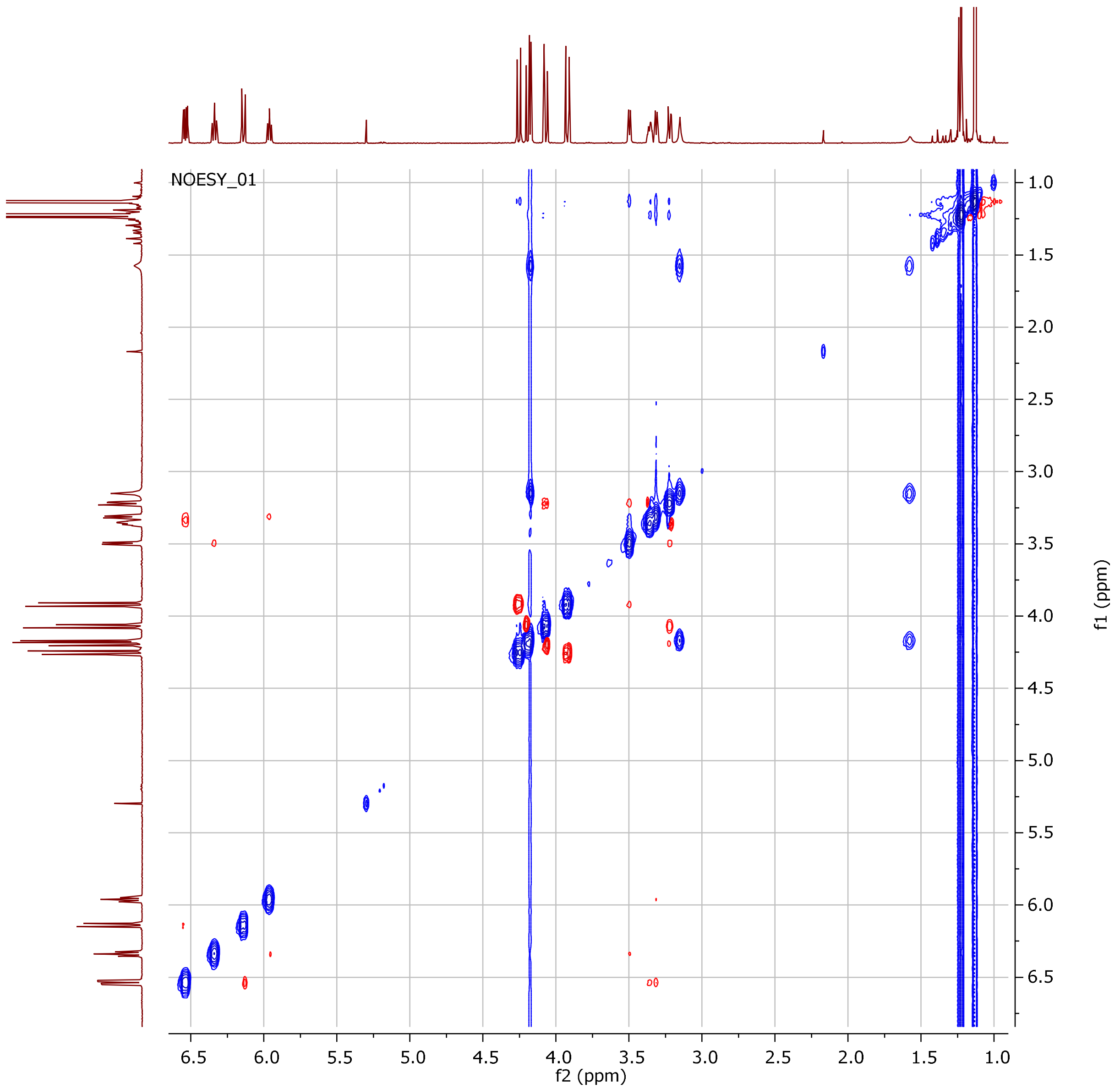

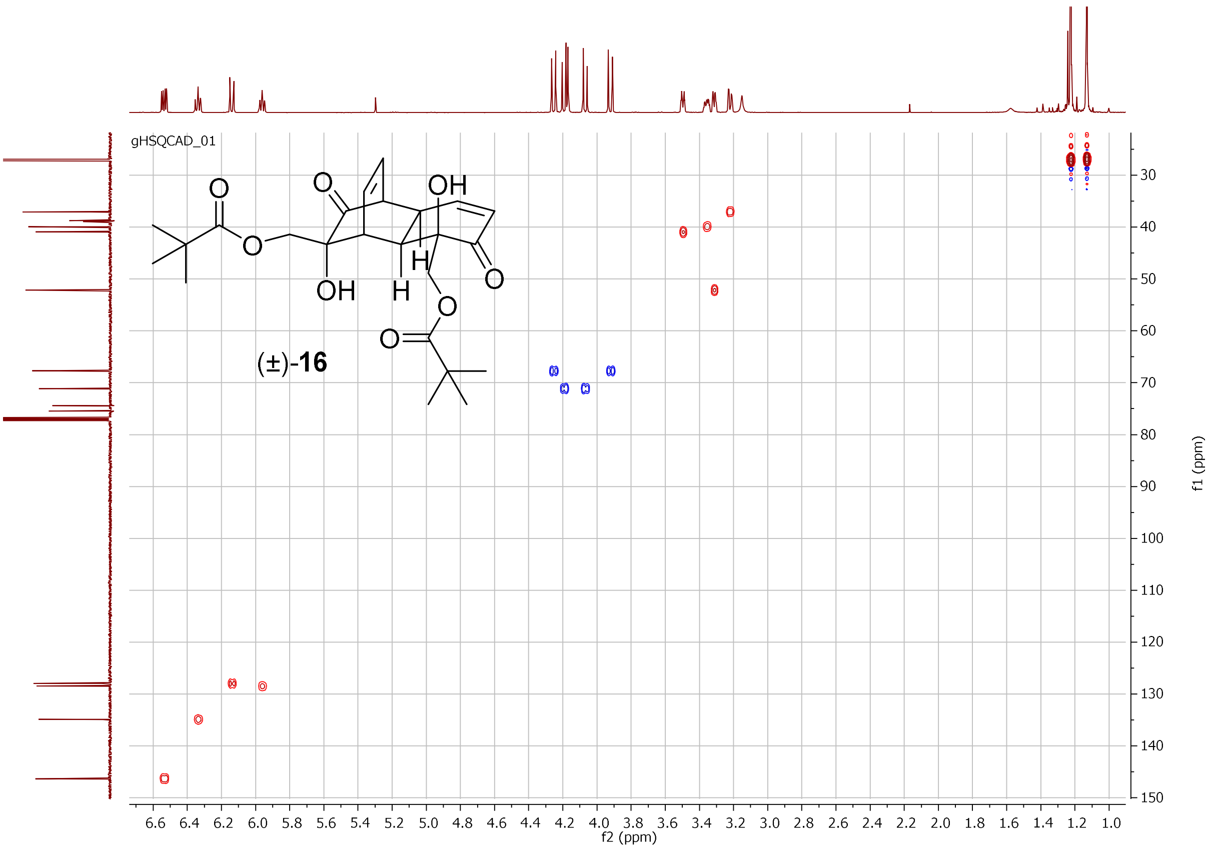
gHSQCAD_01
(±)-16
f2 (ppm)
f1 (ppm)

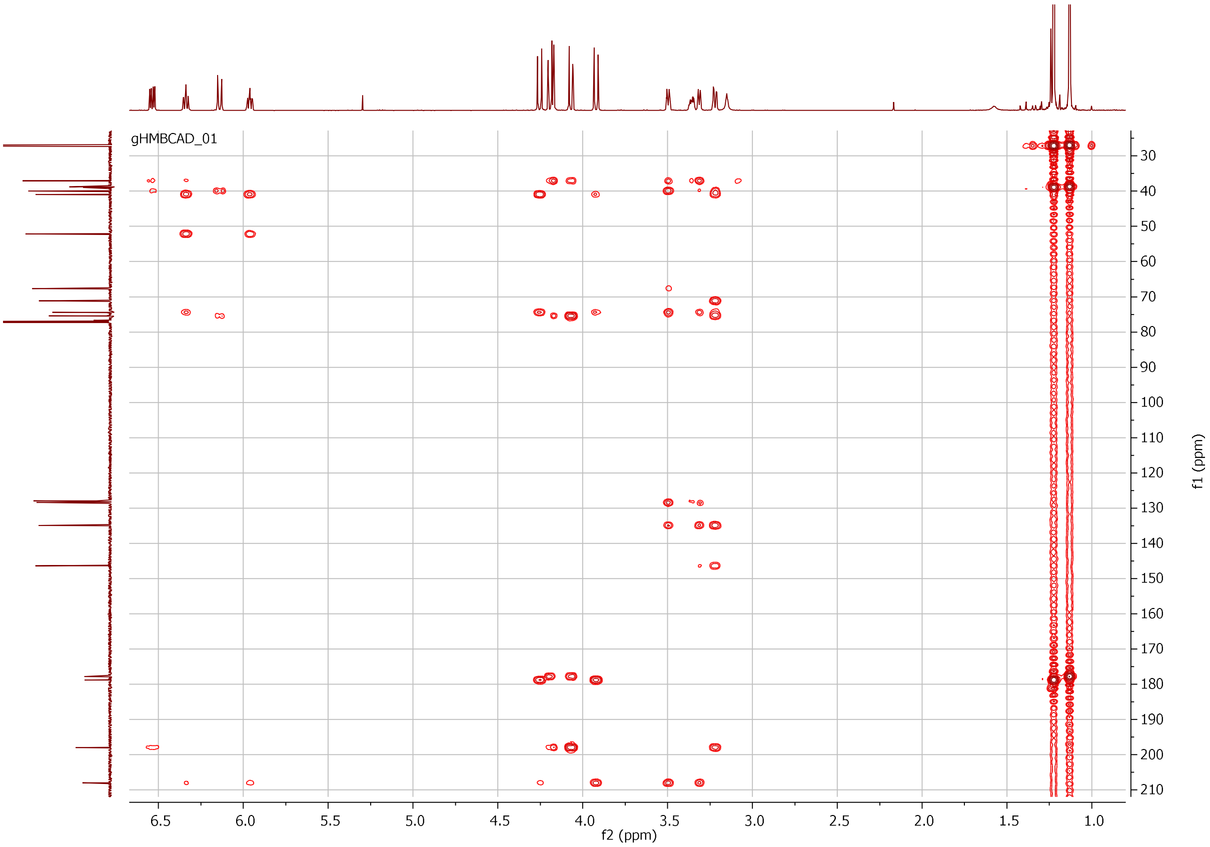
gHMBCAD_01
f2 (ppm)
f1 (ppm)

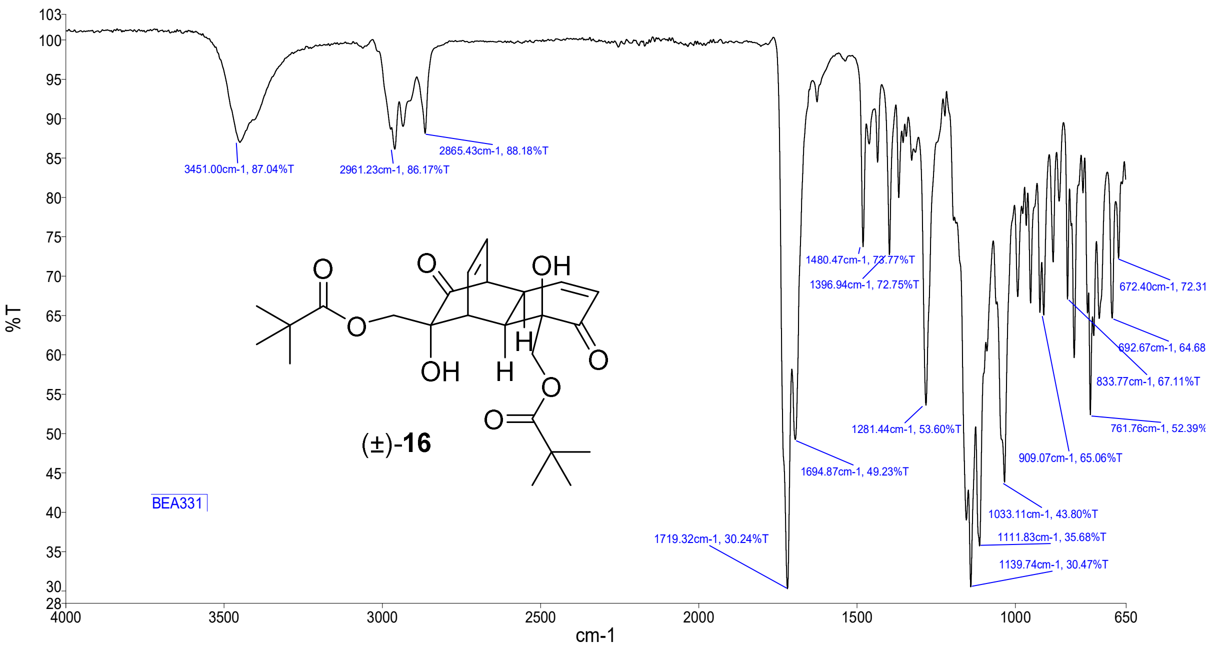

Confirmation of Expected Formula

Sample-ID	ba_sel_BEA331 F	Submitter	bea23 Ben Alexander
Analysis Name	ba_sel_BEA331 F_356048_42_01_62049.d	Supervisor	sl288 Simon Lewis
Method used	Confirm Formula Negative 50to500 loop inj.m	Acquisition Date	27/02/2018 12:35:25
Ionisation Mode	negative electrospray (ESI)		

-MS, 1.0-1.3min #(40-51), -Spectral Bkgrnd

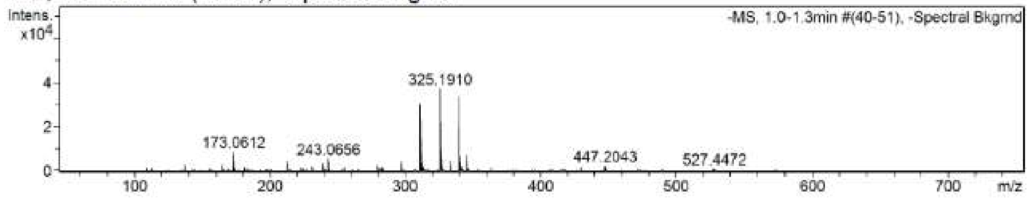

#	m/z	I	I %	Area	S/N
1	173.0612	8677	23.5	315	1302.8
2	243.0656	5532	15.0	261	814.8
3	297.1576	4622	12.5	172	231.5
4	311.1761	30225	81.8	1152	1403.8
5	325.1910	36966	100.0	1534	1940.4
6	326.1937	9957	26.9	323	527.6
7	333.1345	4846	13.1	277	274.6
8	339.2061	33805	91.5	1338	2040.2
9	340.2100	7048	19.1	286	430.0
10	345.1341	7565	20.5	466	487.5

Generate Molecular Formula Parameters

Charge	Tolerance	SearchRadius	H/C Ratio min.	H/C Ratio max.	Electron Conf.	Nitrogen Rule	sigma limit
negative	10 ppm	0.05 m/z	0	3	both	true	0.05

Expected Formula C24 H32 O8 **Adduct(s):** H, Na

#	meas. m/z	theo. m/z	Err[ppm]	Sigma	Formula
1	447.2043	447.2024	4.10	0.0349	C 24 H 31 O 8
1	471.2045	471.2000	9.40	0.0251	C 24 H 32 Na 1 O 8

Note: Sigma fits < 0.05 indicates high probability of correct MF.

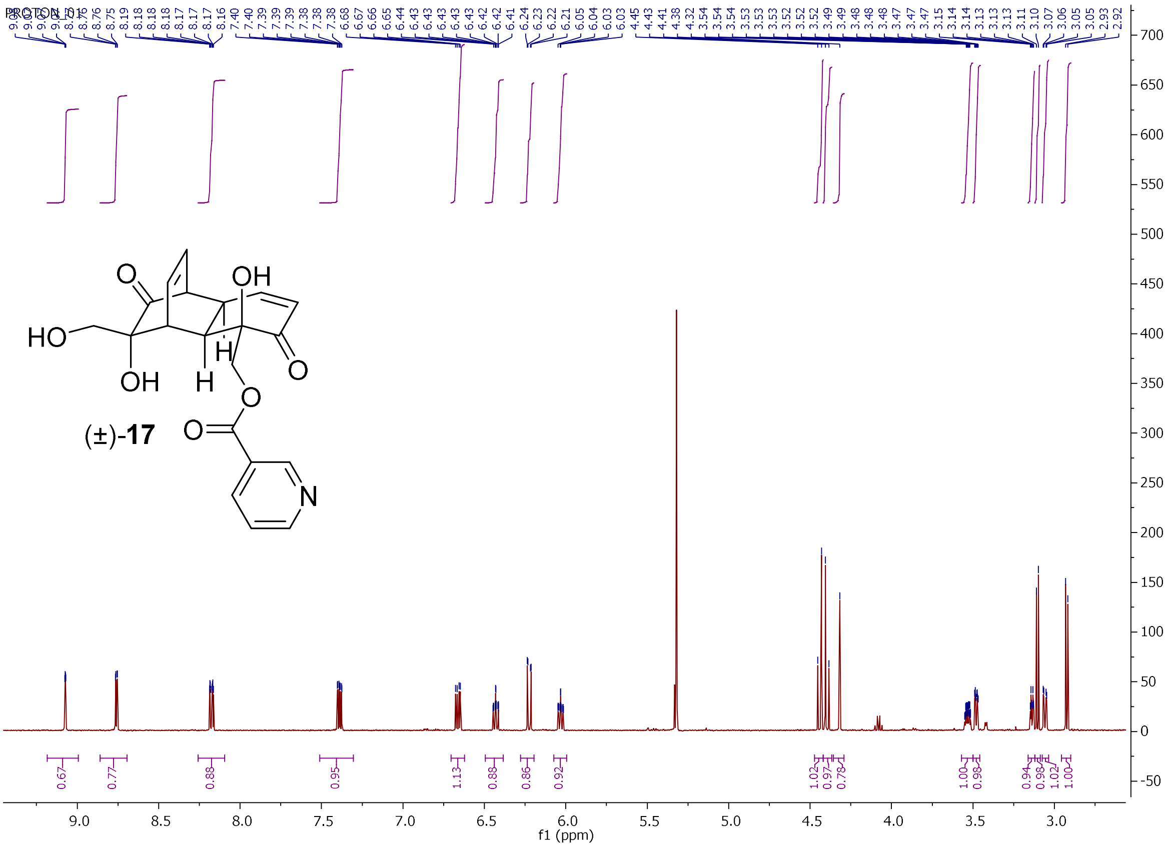
PROTON_01
HO
OH
O
H
(±)-17
N
f1 (ppm)

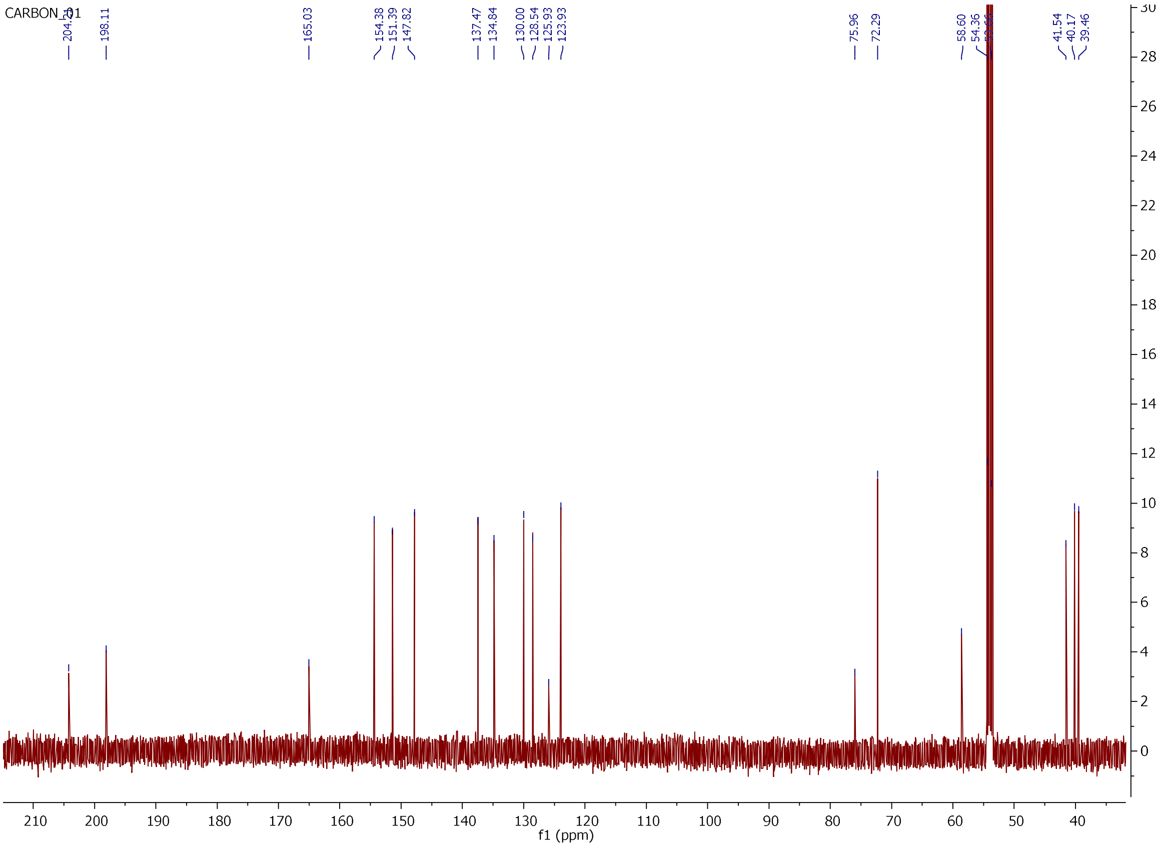
CARBON_01
198.11
165.03
154.38
151.39
147.82
137.47
134.84
130.00
128.54
125.93
123.93
75.96
72.29
58.60
54.36
41.54
40.17
39.46
f1 (ppm)

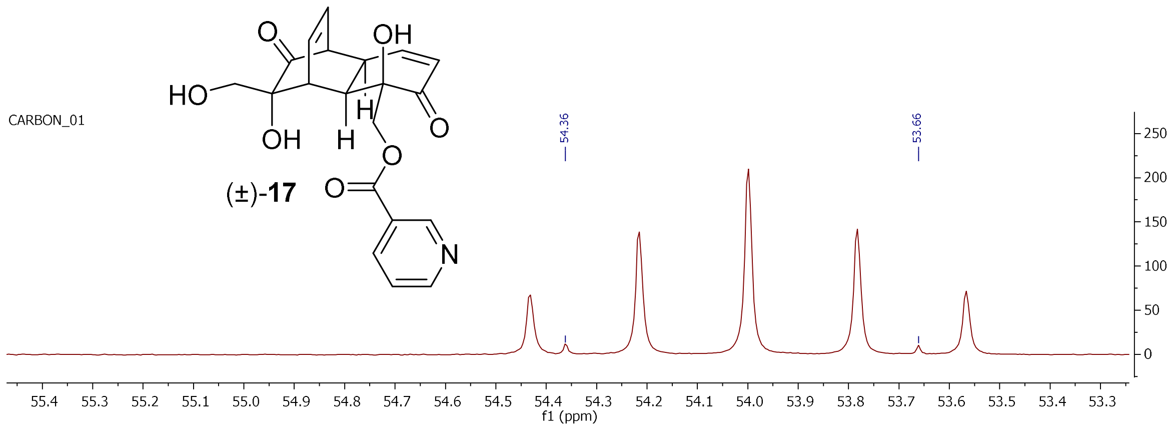

(±)-**17**

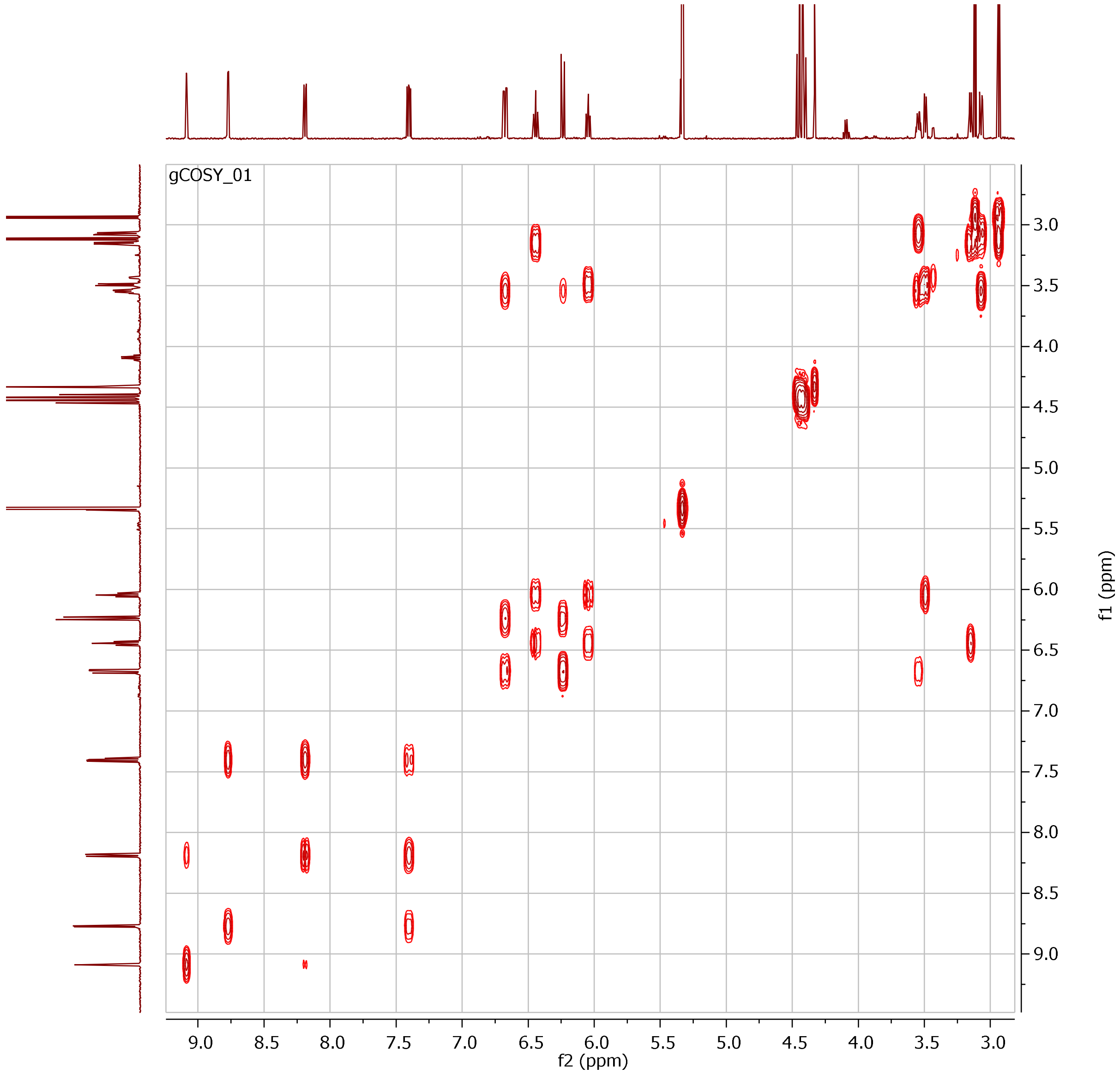

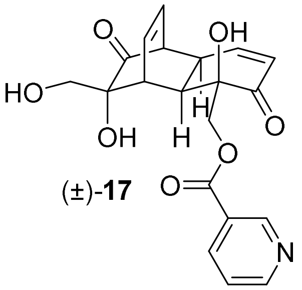
HO
OH
O
OH
H
H
O
O
O
N

(±)-**17**

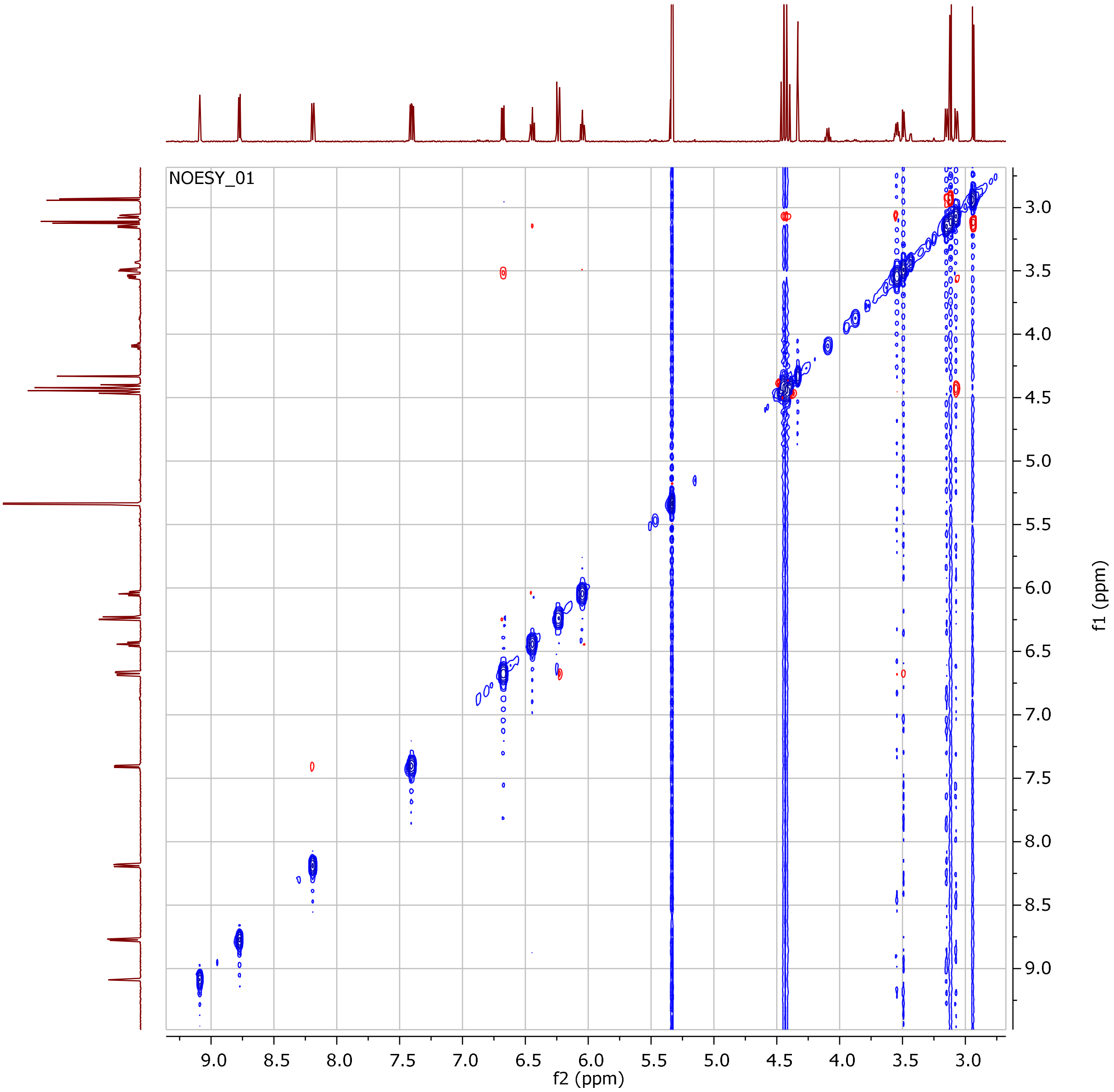
NOESY_01
f2 (ppm)
f1 (ppm)
9.0
8.5
8.0
7.5
7.0
6.5
6.0
5.5
5.0
4.5
4.0
3.5
3.0

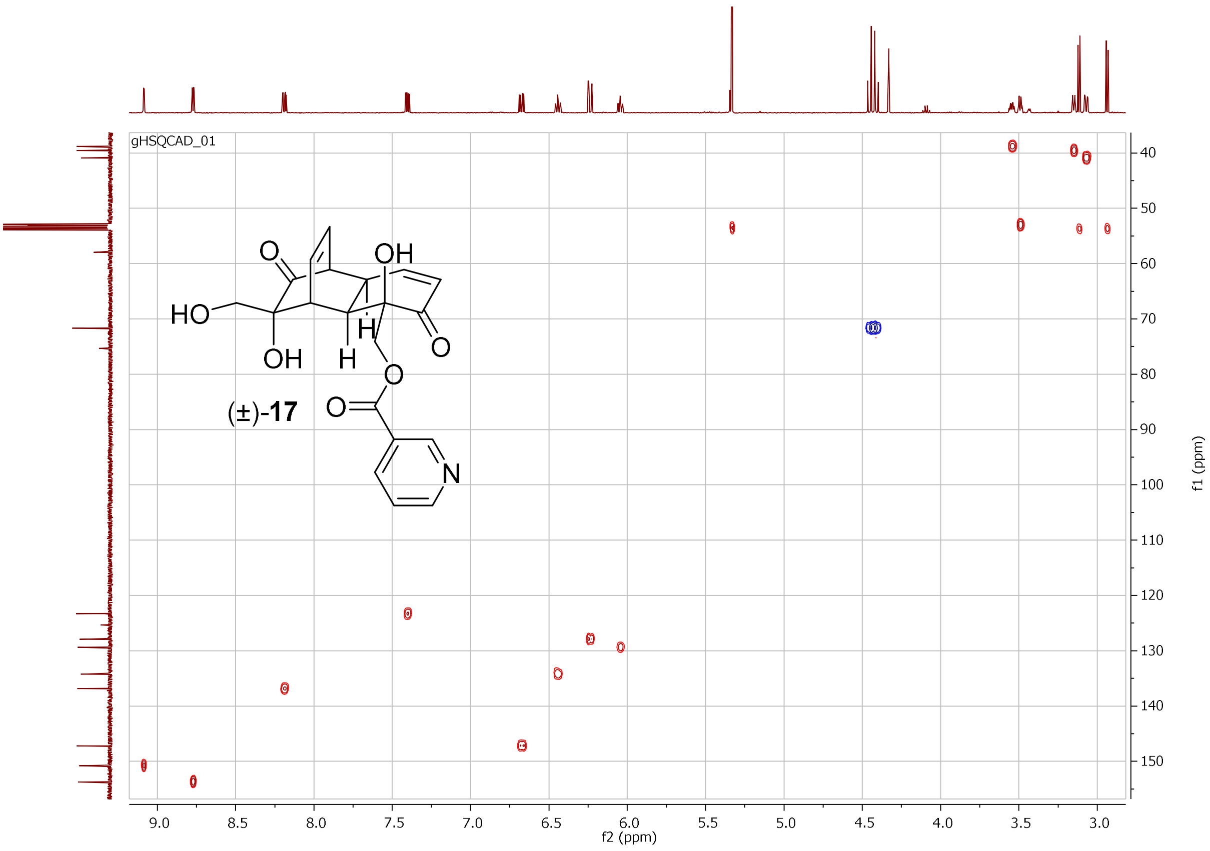
gHSQCAD_01
HO
OH
O
OH
H
H
O
O
O
N
(±)-17
f2 (ppm)
f1 (ppm)

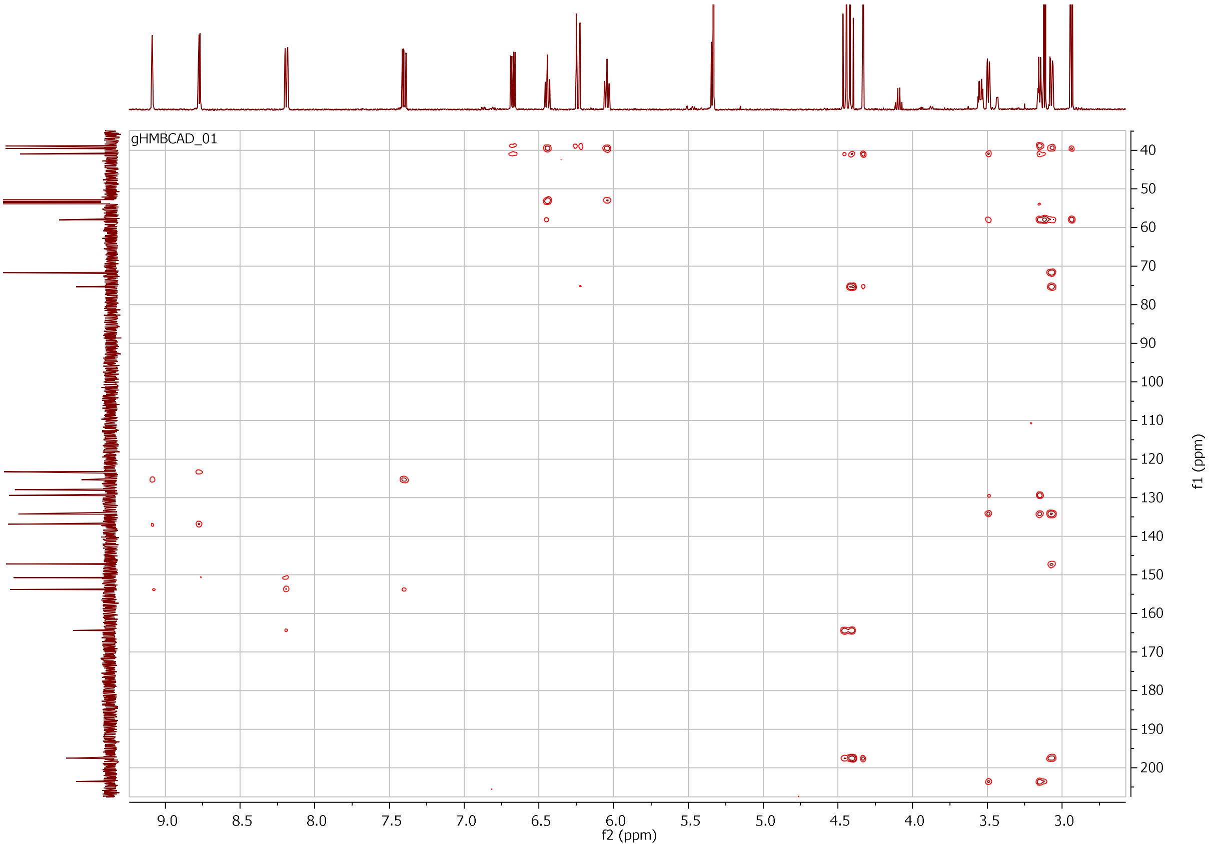
gHMBCAD_01
f2 (ppm)
f1 (ppm)

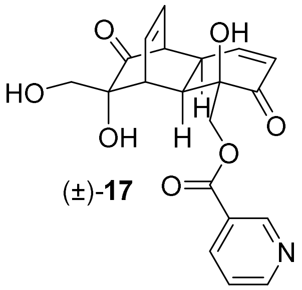

(±)-**17**

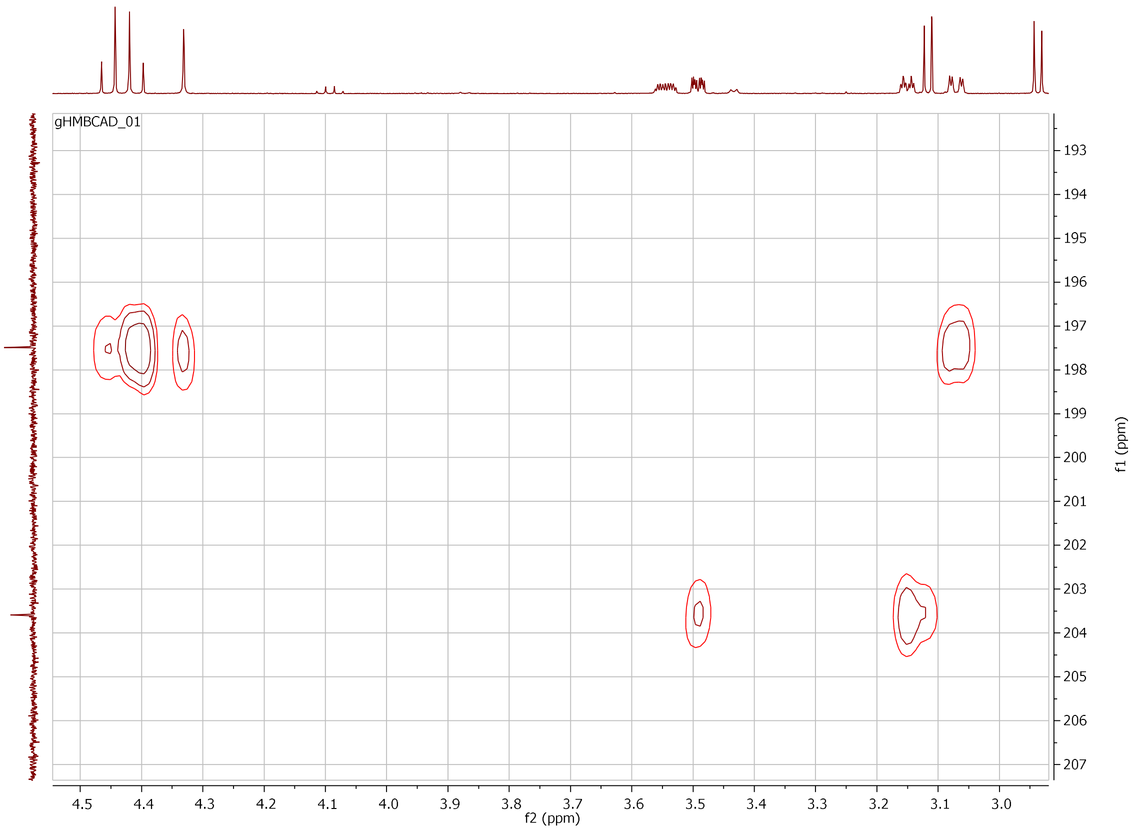

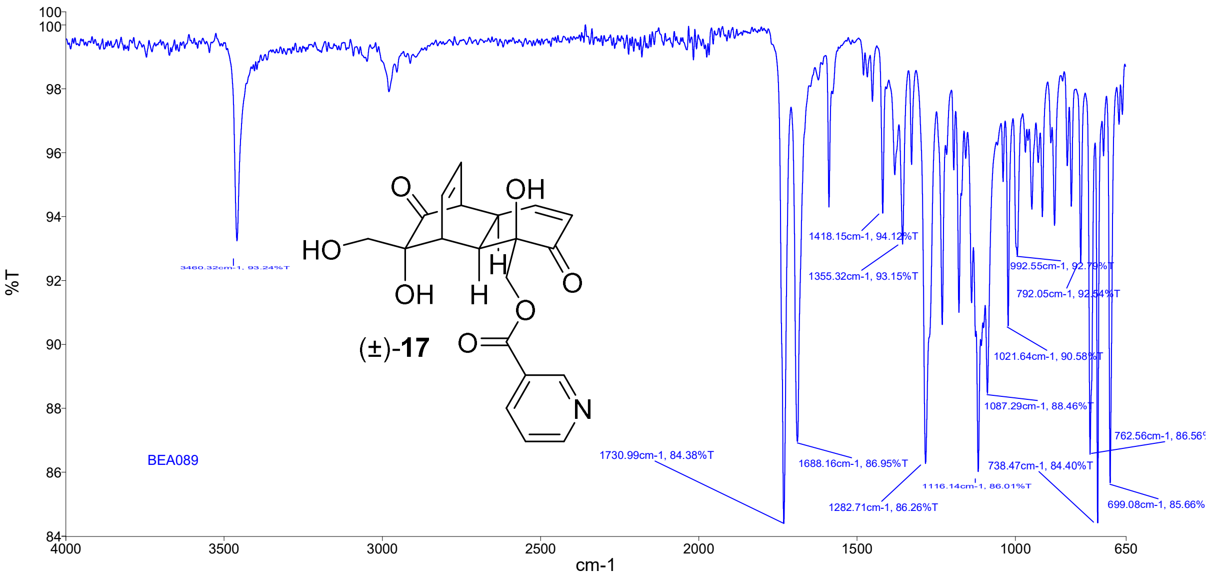

Mass Spectrum SmartFormula Report

Analysis Info

Analysis Name	Z:\ba_sel_BEA089_358361_42_01_64739.d	Acquisition Date	8/14/2018 11:22:07 AM
Method	Confirm Formula Positive 50to500 loop inj.m	Operator	admin
Sample Name	ba_sel_BEA089_358361	Instrument / Ser#	micrOTOF 161
Comment			

Acquisition Parameter

Source Type	ESI	Ion Polarity	Positive	Set Nebulizer	2.2 Bar
Focus	Not active			Set Dry Heater	220 °C
Scan Begin	50 m/z	Set Capillary	4500 V	Set Dry Gas	10.2 l/min
Scan End	700 m/z	Set End Plate Offset	-500 V	Set Divert Valve	Source

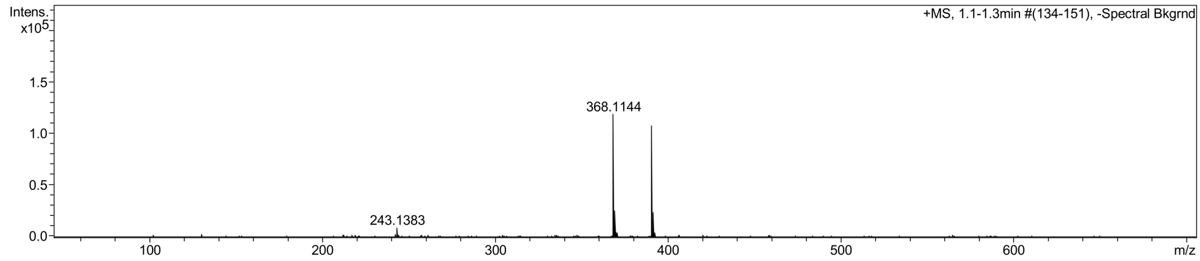

Meas. m/z	#	Formula	Score	m/z	err [mDa]	err [ppm]	mSigma	rdb	e⁻ Conf	N-Rule
368.1144	1	C 20 H 18 N O 6	63.60	368.1129	-1.5	-4.1	5.1	12.5	even	ok
	2	C 17 H 10 N 11	20.35	368.1115	-2.9	-7.8	8.5	18.5	even	ok
	3	C 21 H 14 N 5 O 2	100.00	368.1142	-0.2	-0.5	19.3	17.5	even	ok
	4	C 26 H 14 N 3	2.92	368.1182	3.9	10.5	46.6	21.5	even	ok

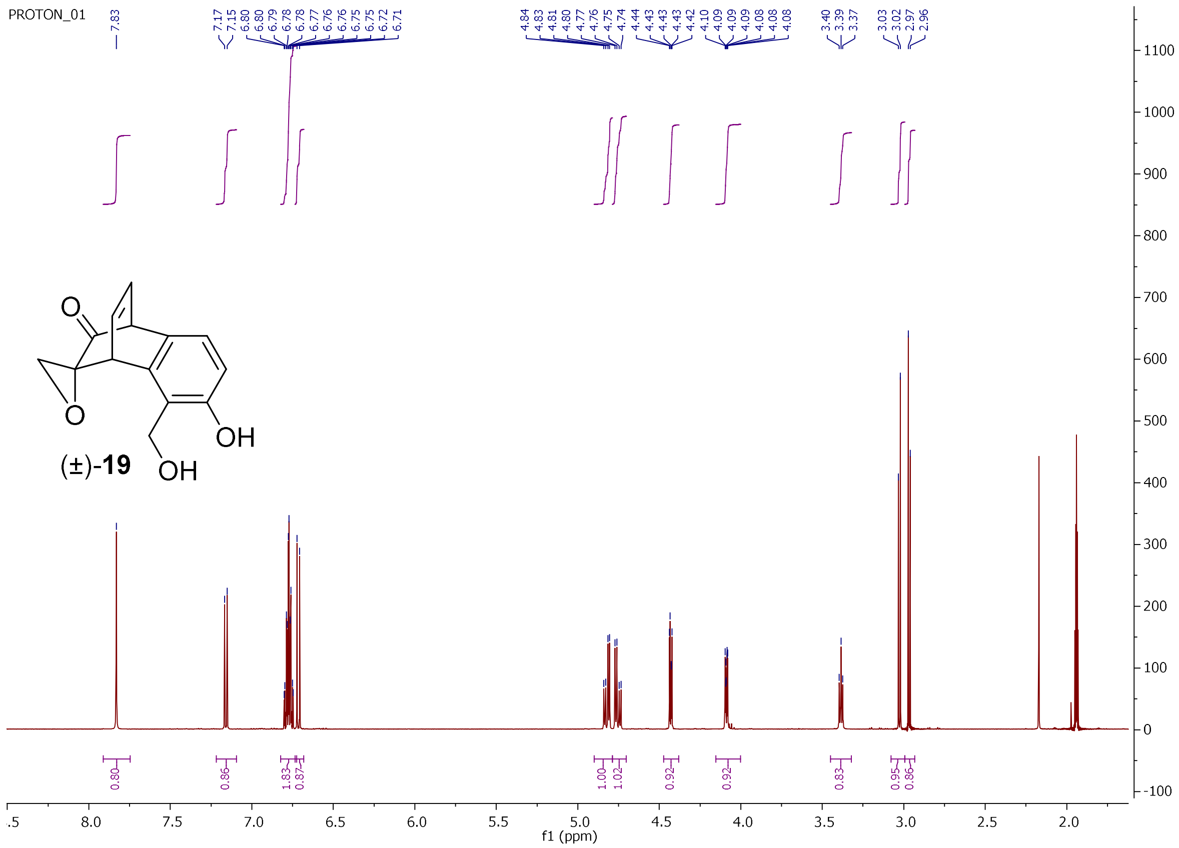
PROTON_01
7.83
7.17
7.15
6.80
6.79
6.78
6.78
6.77
6.76
6.76
6.75
6.75
6.72
6.71
4.84
4.83
4.81
4.80
4.77
4.76
4.75
4.74
4.44
4.43
4.43
4.43
4.43
4.42
4.10
4.09
4.09
4.09
4.09
4.08
4.08
4.08
3.40
3.39
3.37
3.03
3.02
2.97
2.96
O
O
OH
OH
(±)-19
0.80
0.86
1.83
0.87
1.00
1.02
0.92
0.92
0.83
0.95
0.86
f1 (ppm)

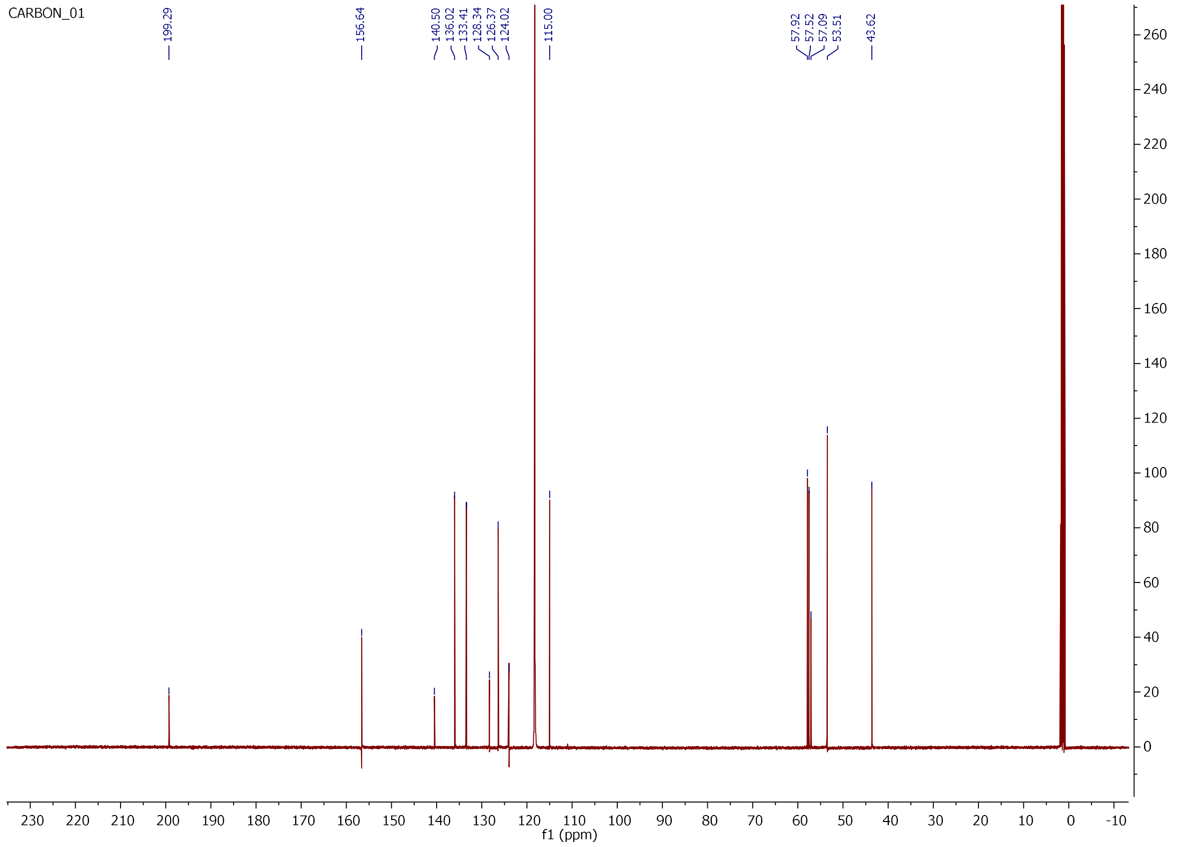
CARBON_01
199.29
156.64
140.50
136.02
133.41
128.34
126.37
124.02
115.00
57.92
57.52
57.09
53.51
43.62
f1 (ppm)

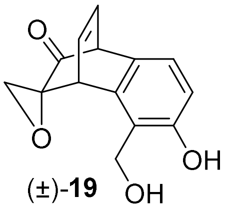

(±)-**19**

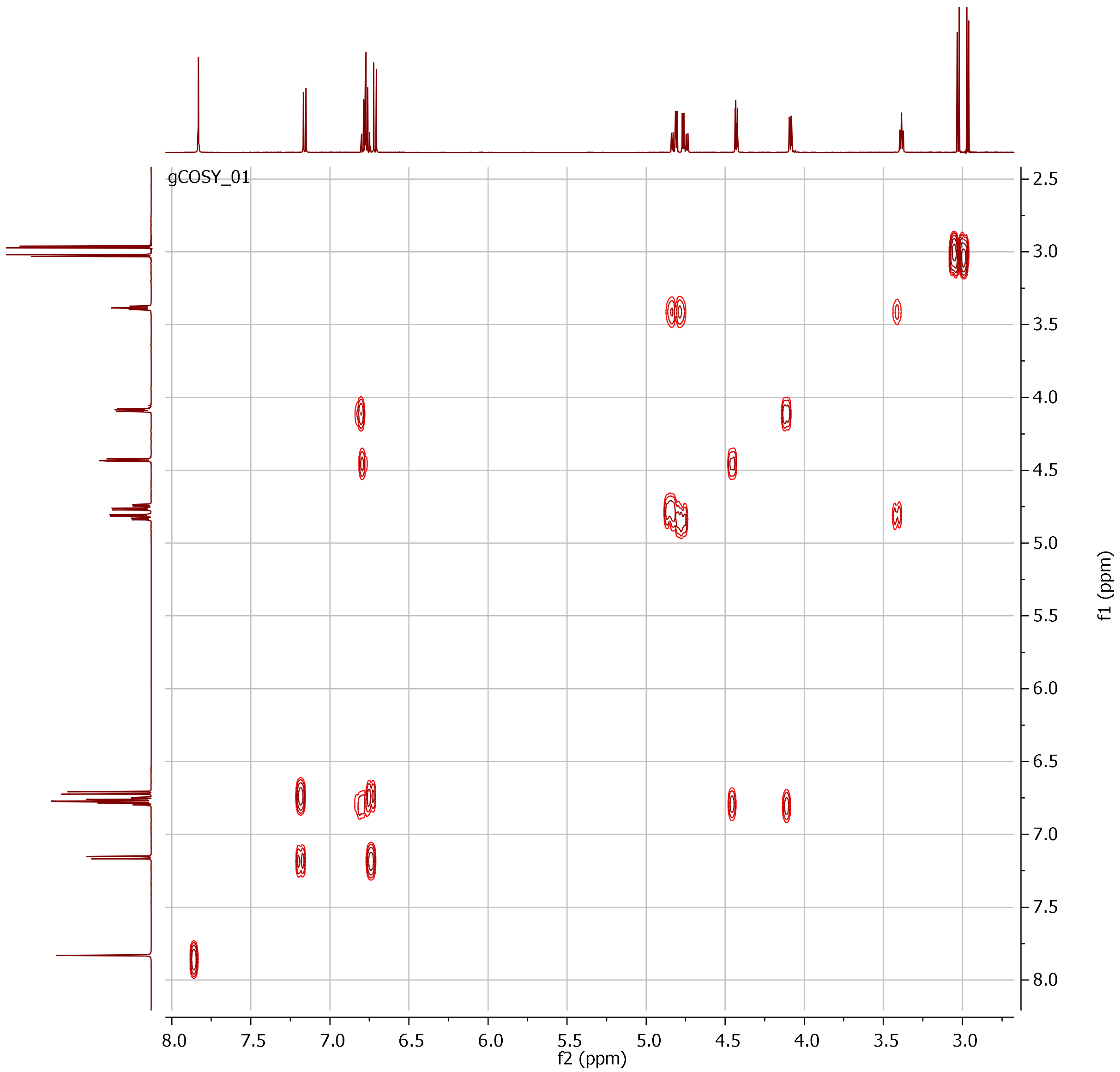

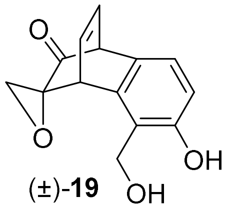

(±)-**19**

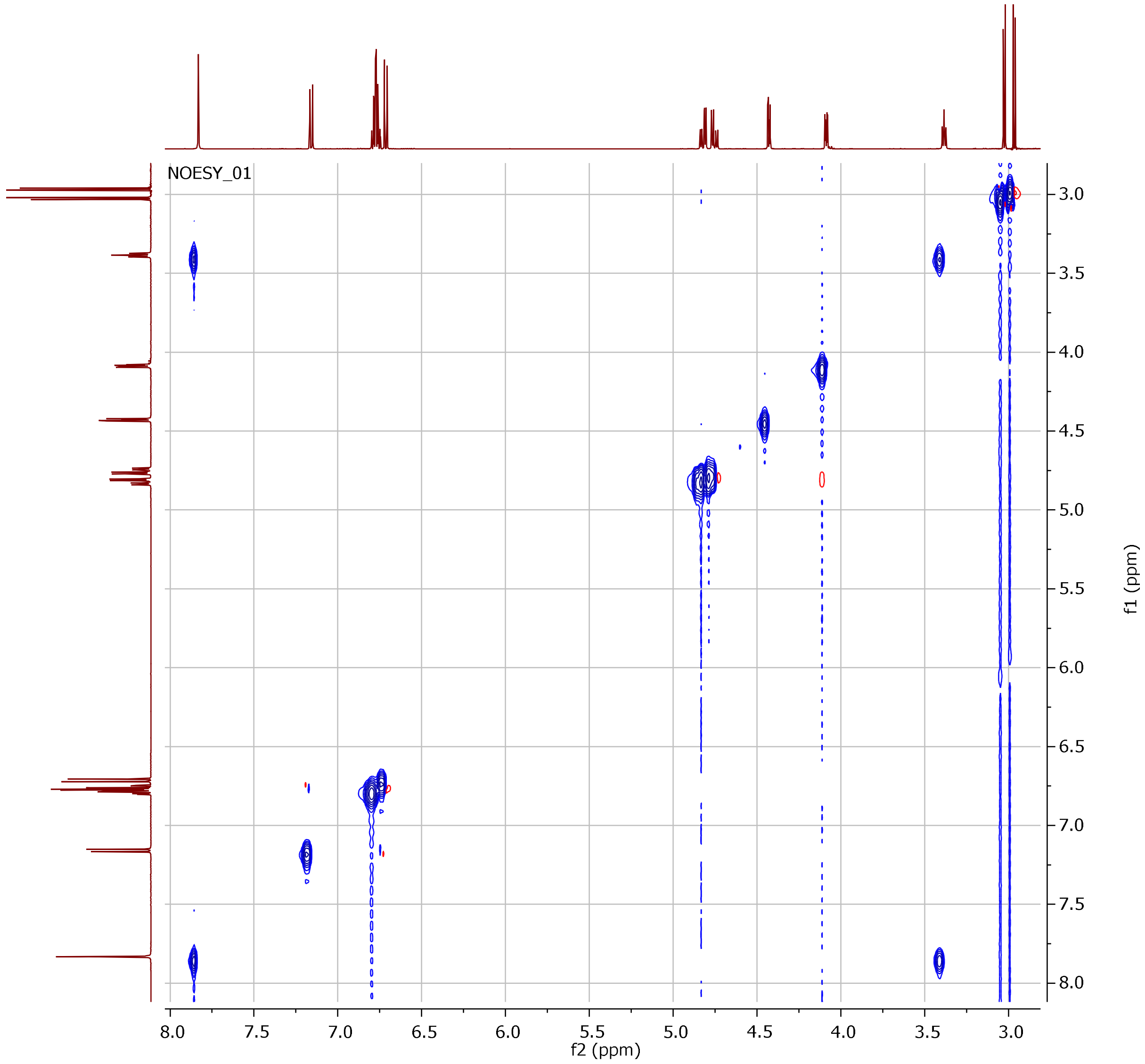

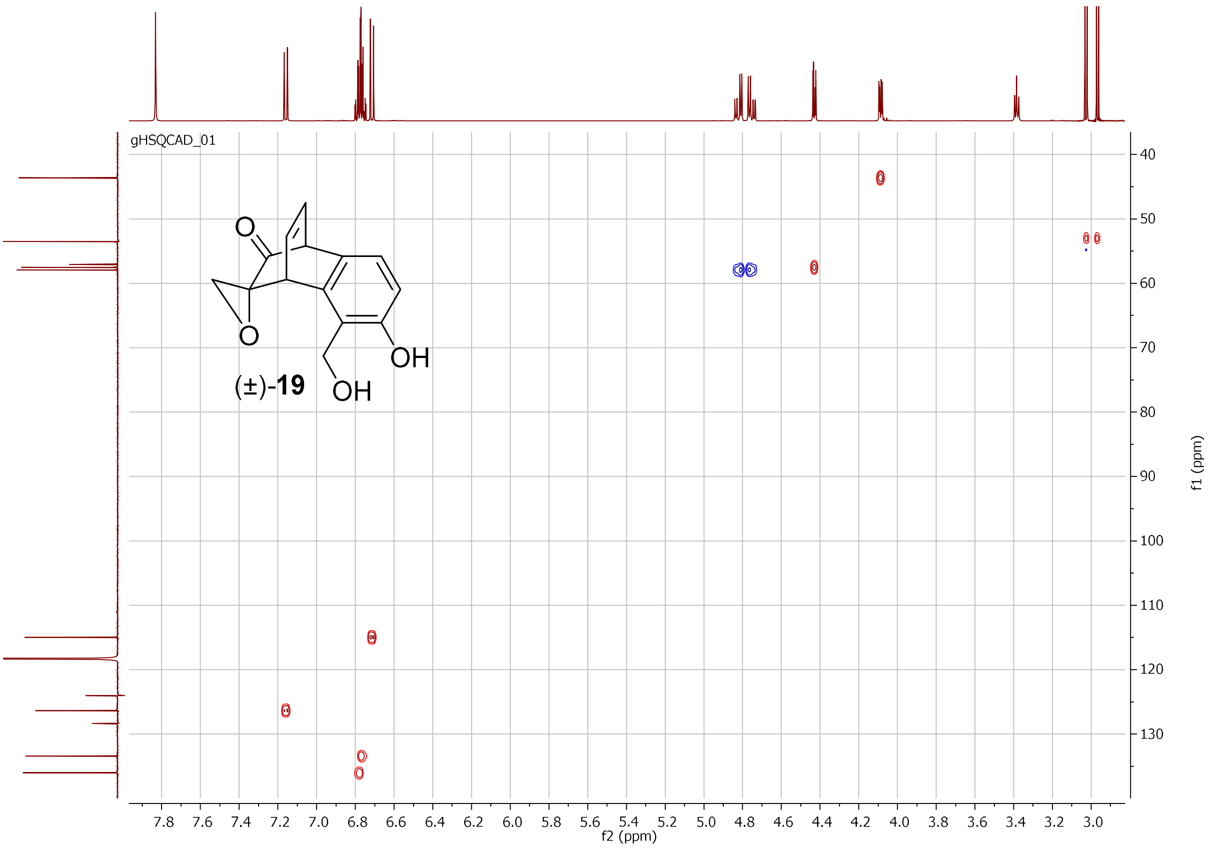
gHSQCAD_01
O
O
OH
OH
(±)-19
f2 (ppm)
f1 (ppm)

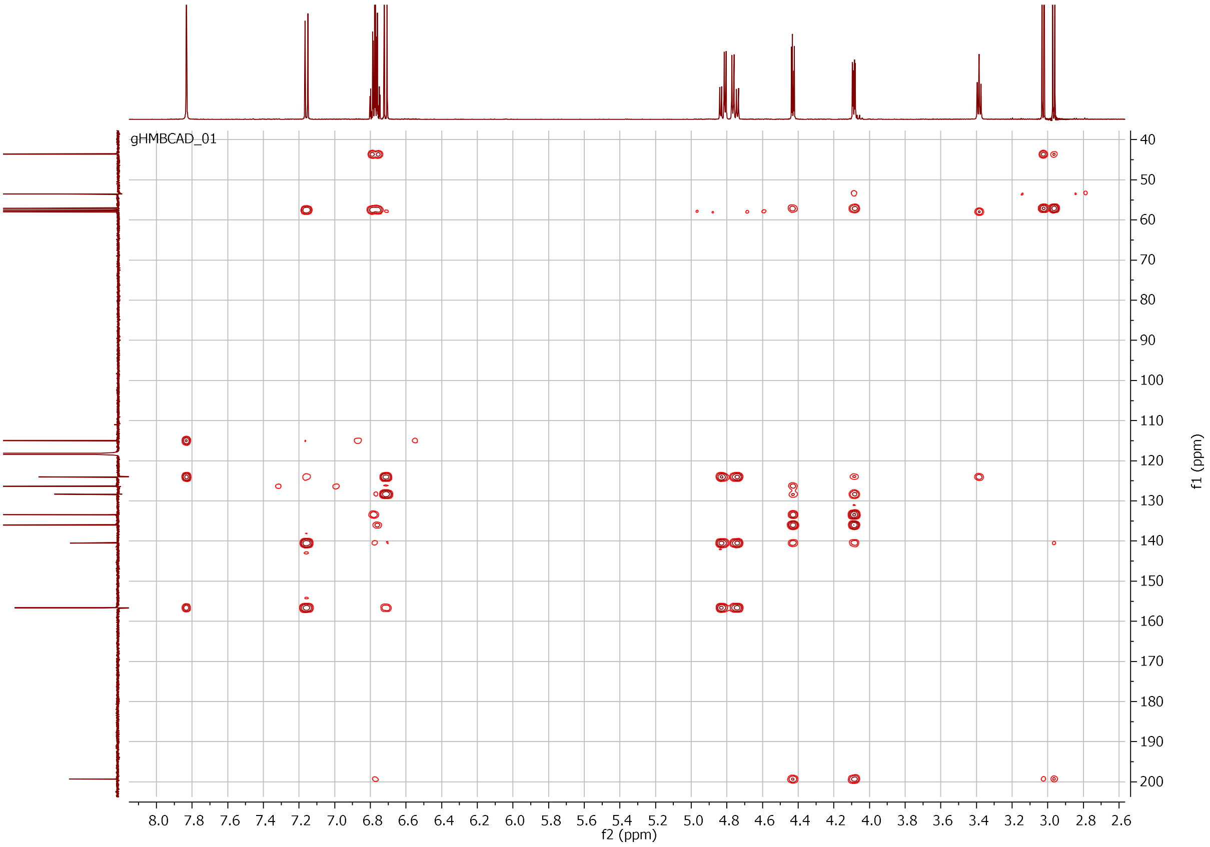
gHMBCAD_01
f2 (ppm)
f1 (ppm)

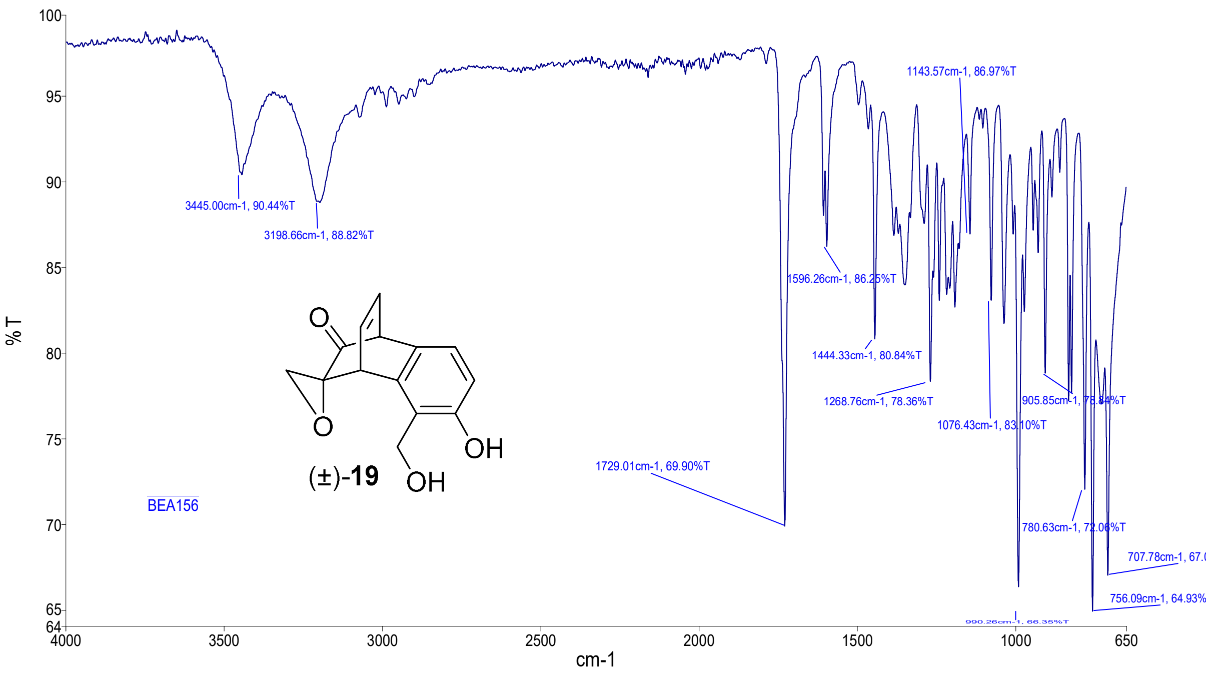

Confirmation of Expected Formula

Sample-ID	ba_sel_BEA156 C	Submitter	bea23 Ben Alexander
Analysis Name	ba_sel_BEA156 C_349496_72_01_54605.d	Supervisor	sl288 Simon Lewis
Method used	Confirm Formula Positive 50to500 loop inj.m	Acquisition Date	28/10/2016 10:16:57
Ionisation Mode	positive electrospray (ESI)		

+MS, 1.0-1.3min #(60-77), -Spectral Bkgrnd

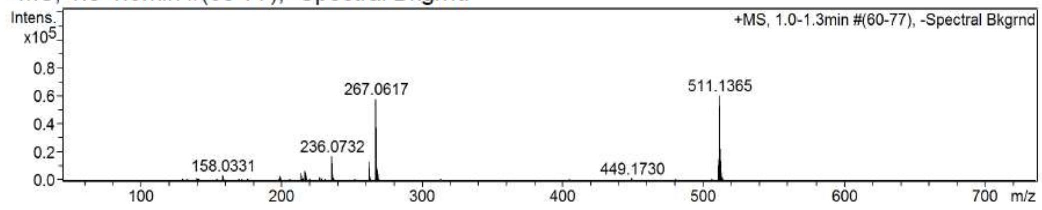

#	m/z	I	I %	Area	S/N
1	158.0331	3424	5.7	50	1926.9
2	199.0745	3197	5.3	111	640.2
3	214.1018	5130	8.5	120	773.7
4	217.0845	6870	11.4	277	977.9
5	236.0732	17062	28.3	370	1788.0
6	262.1056	13123	21.8	714	1405.5
7	267.0617	57830	95.9	2837	7041.7
8	268.0649	7594	12.6	404	951.0
9	511.1365	60332	100.0	5259	10493.2
10	512.1383	16337	27.1	1634	2764.4

Generate Molecular Formula Parameters

Charge	Tolerance	SearchRadius	H/C Ratio min.	H/C Ratio max.	Electron Conf.	Nitrogen Rule	sigma limit
positive	10 ppm	0.05 m/z	0	3	both	true	0.05

Expected Formula C14 H12 O4 **Adduct(s):** H, Na

#	meas. m/z	theo. m/z	Err[ppm]	Sigma	Formula
1	267.0617	267.063329	3.90	0.0123	C 14 H 12 Na 1 O 4

Note: Sigma fits < 0.05 indicates high probability of correct MF, and mass accuracy of 5ppm or better is generally acceptable for publication

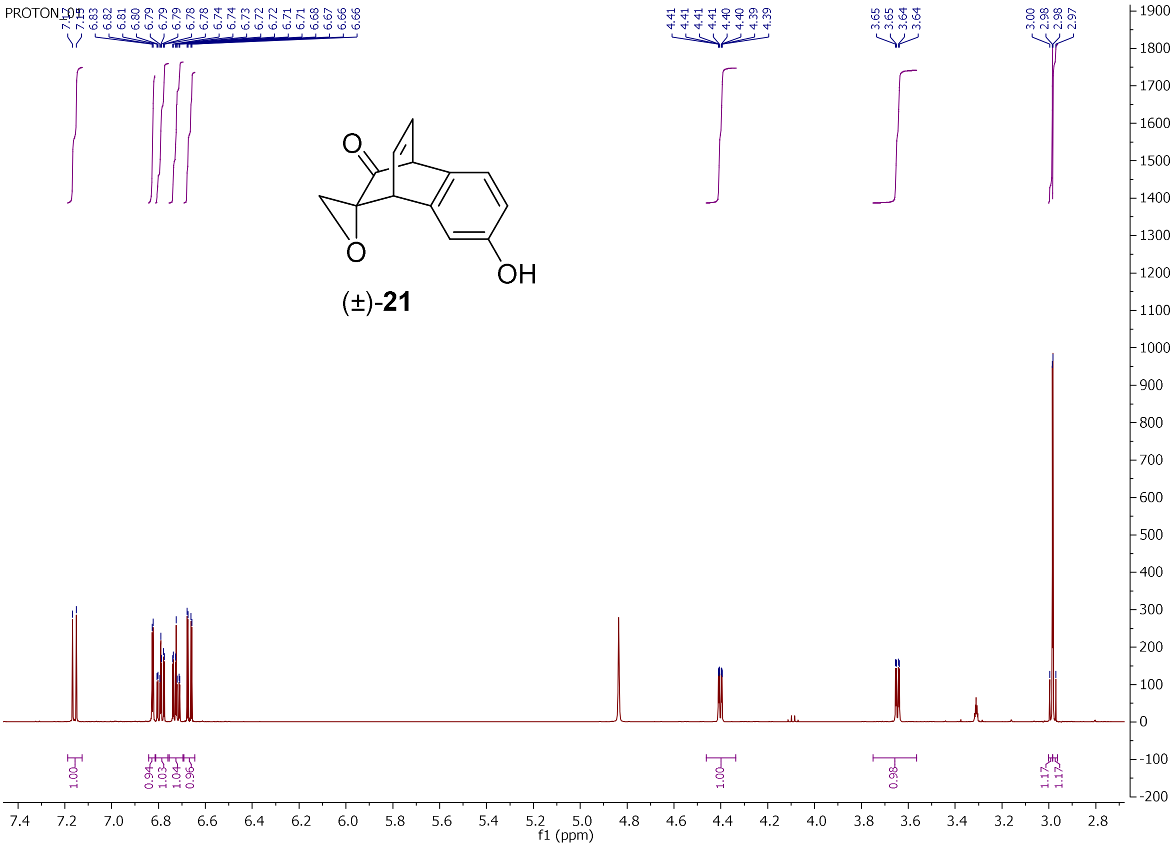
PROTON_01
7.17
7.15
6.83
6.82
6.81
6.80
6.79
6.79
6.79
6.78
6.74
6.74
6.73
6.72
6.72
6.71
6.71
6.68
6.67
6.66
6.66
4.41
4.41
4.41
4.41
4.40
4.40
4.39
4.39
3.65
3.65
3.64
3.64
3.00
2.98
2.98
2.97
O
O
OH
(±)-21
1.00
0.94
1.03
1.04
0.96
1.00
0.98
1.17
1.17
f1 (ppm)

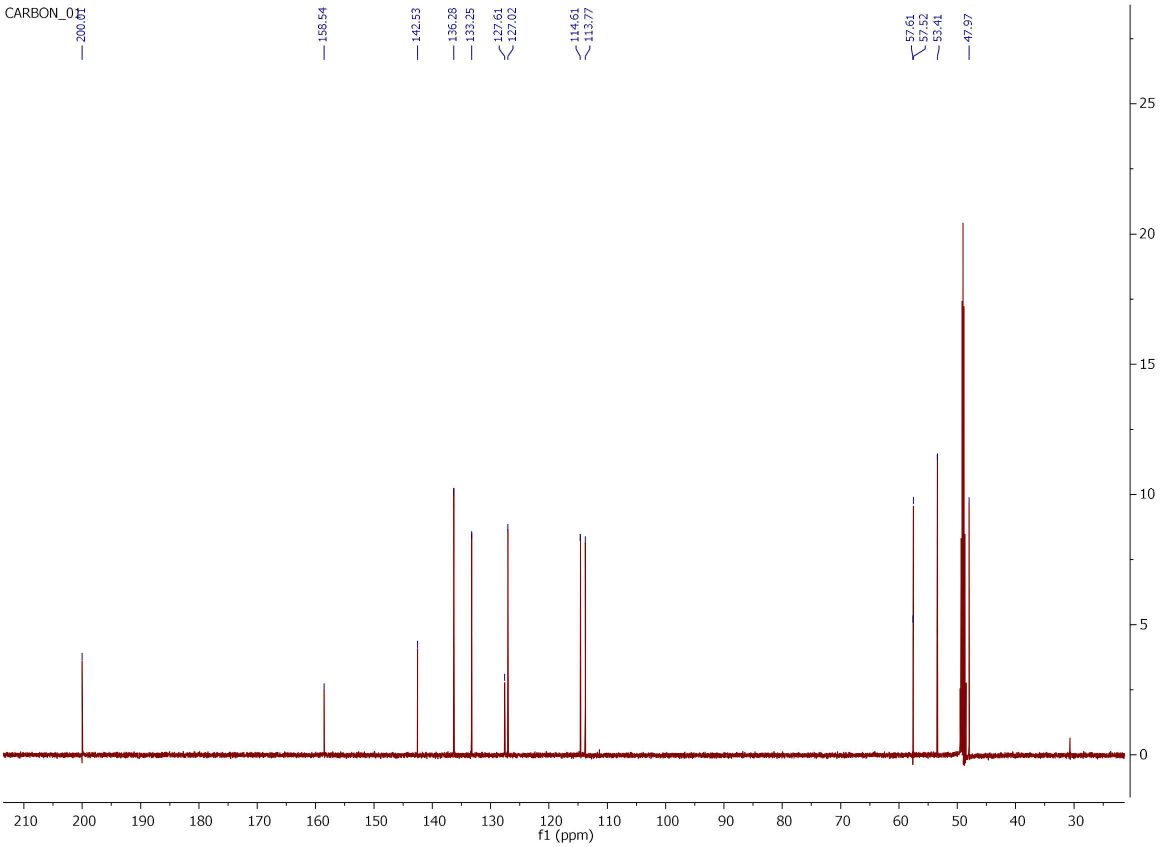
CARBON_01
200.01
158.54
142.53
136.28
133.25
127.61
127.02
114.61
113.77
57.61
57.52
53.41
47.97
f1 (ppm)

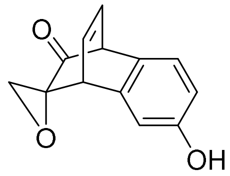
O
O
OH

(±)-**21**

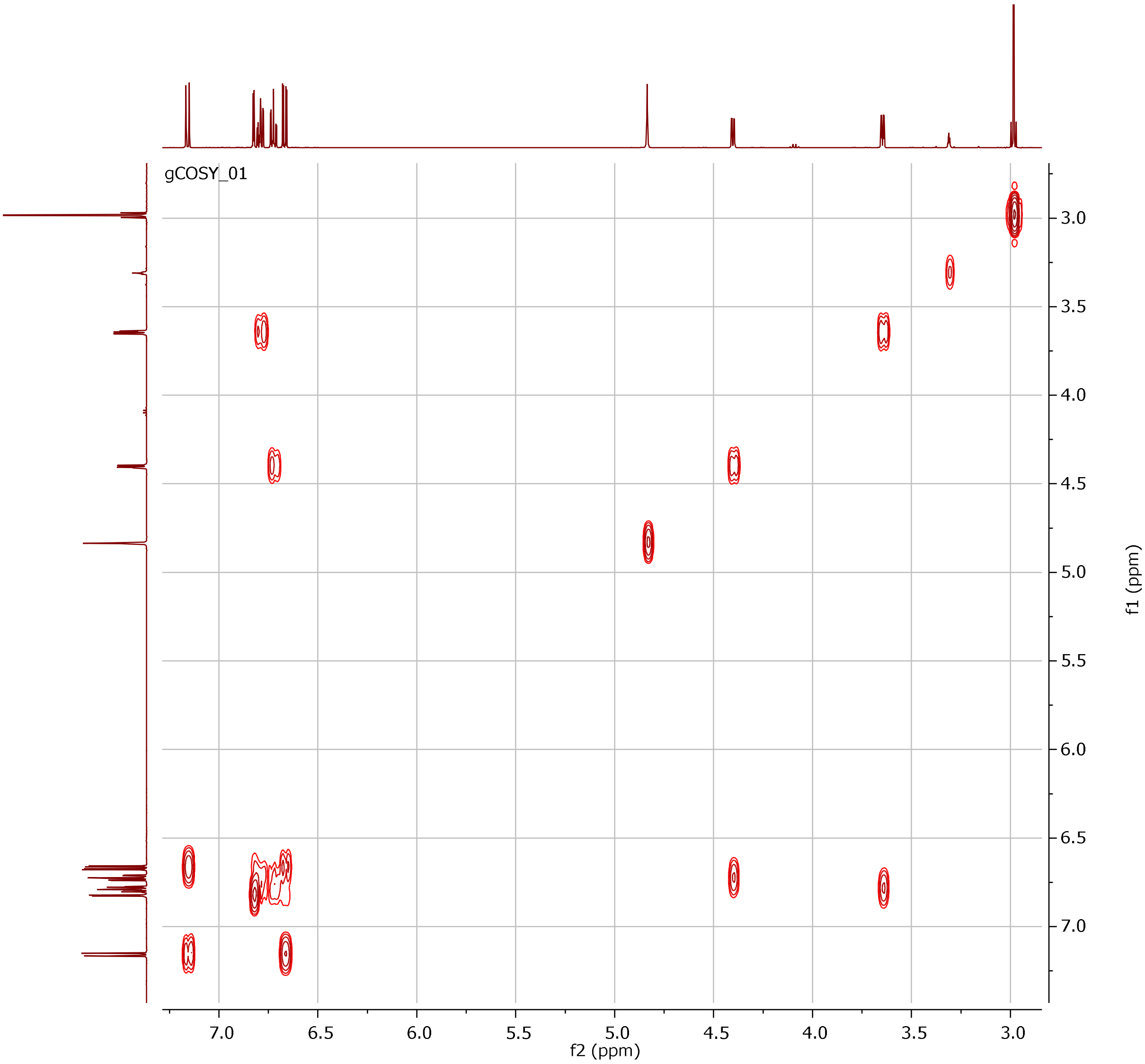
gCOSY_01
3.0
3.5
4.0
4.5
5.0
5.5
6.0
6.5
7.0
f1 (ppm)
7.0
6.5
6.0
5.5
5.0
4.5
4.0
3.5
3.0
f2 (ppm)

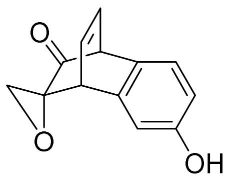

(±)-**21**

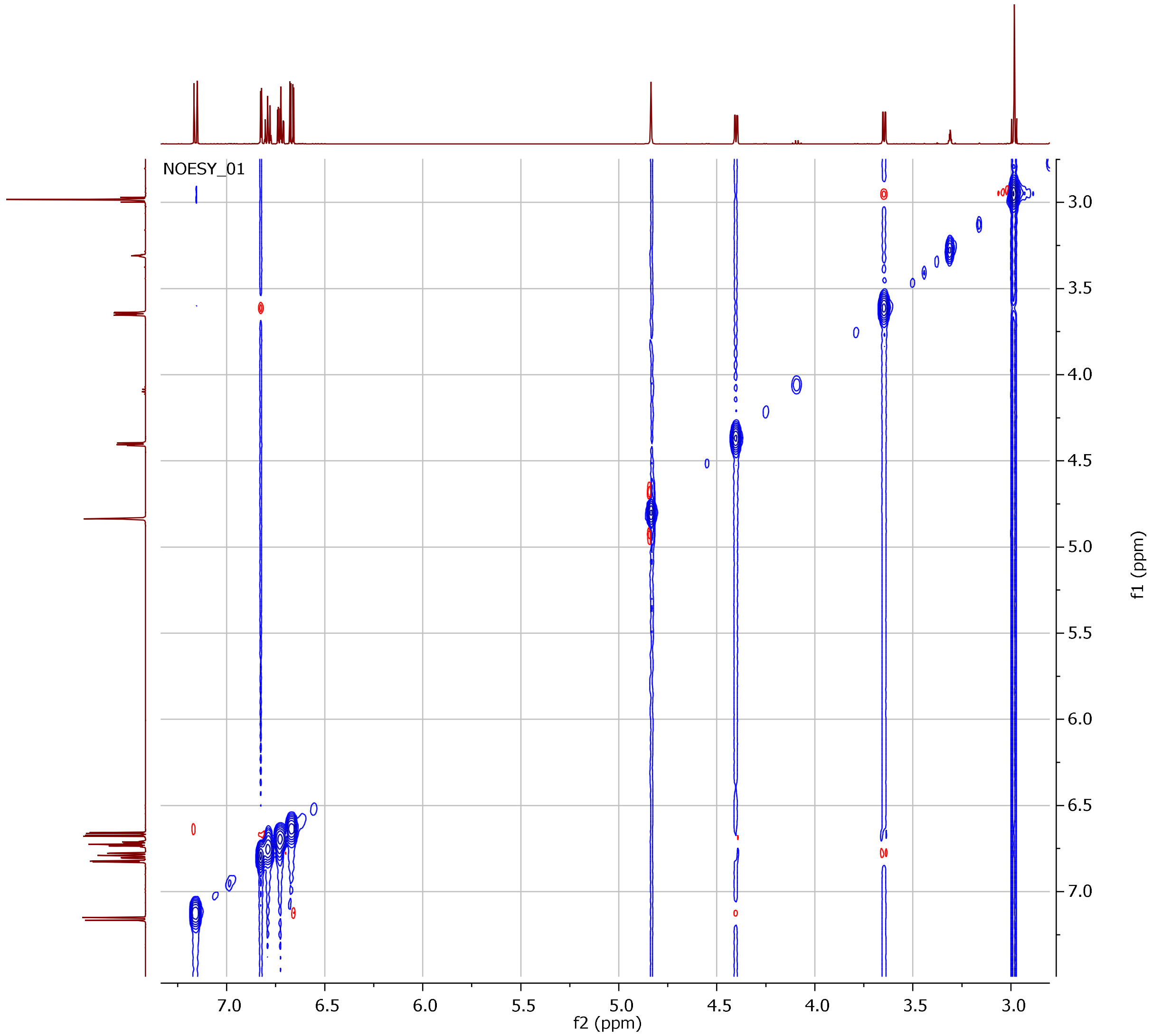

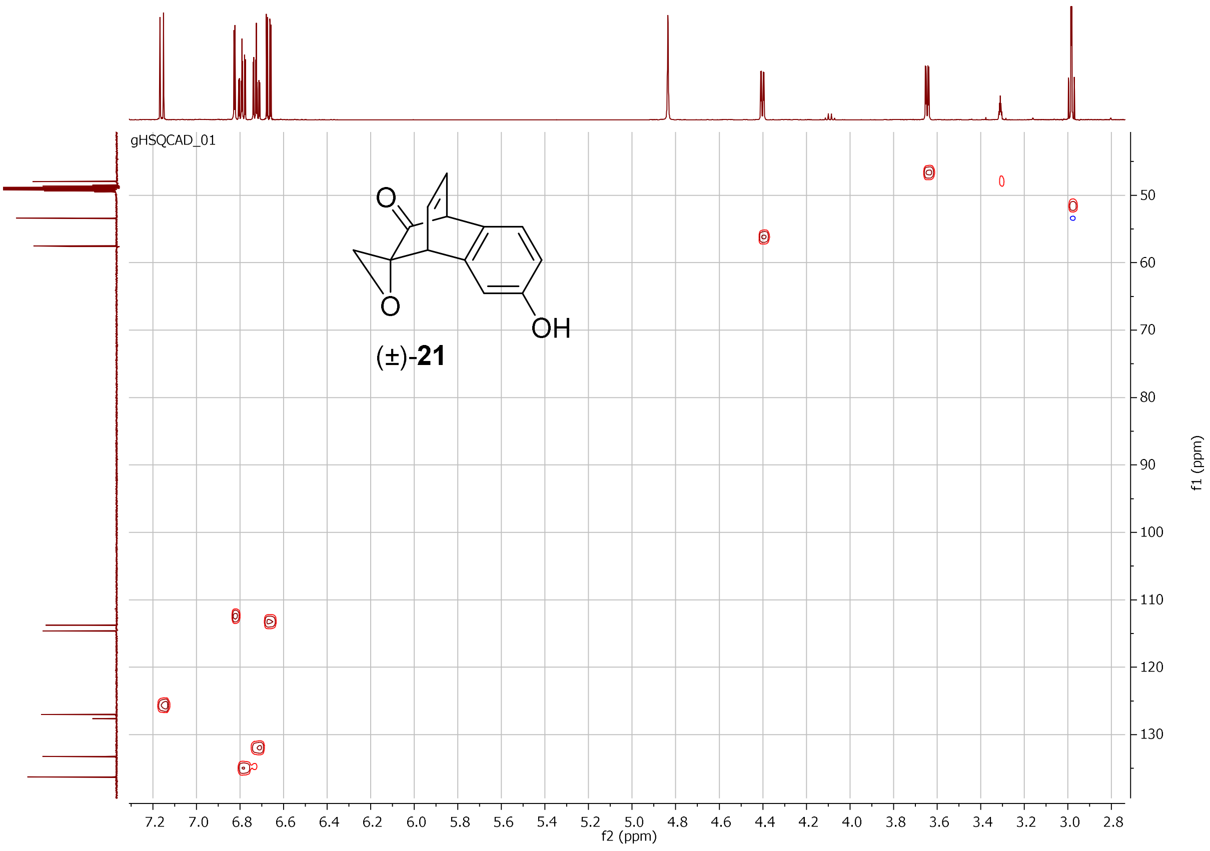
gHSQCAD_01
O
O
OH
(±)-21
f2 (ppm)
f1 (ppm)

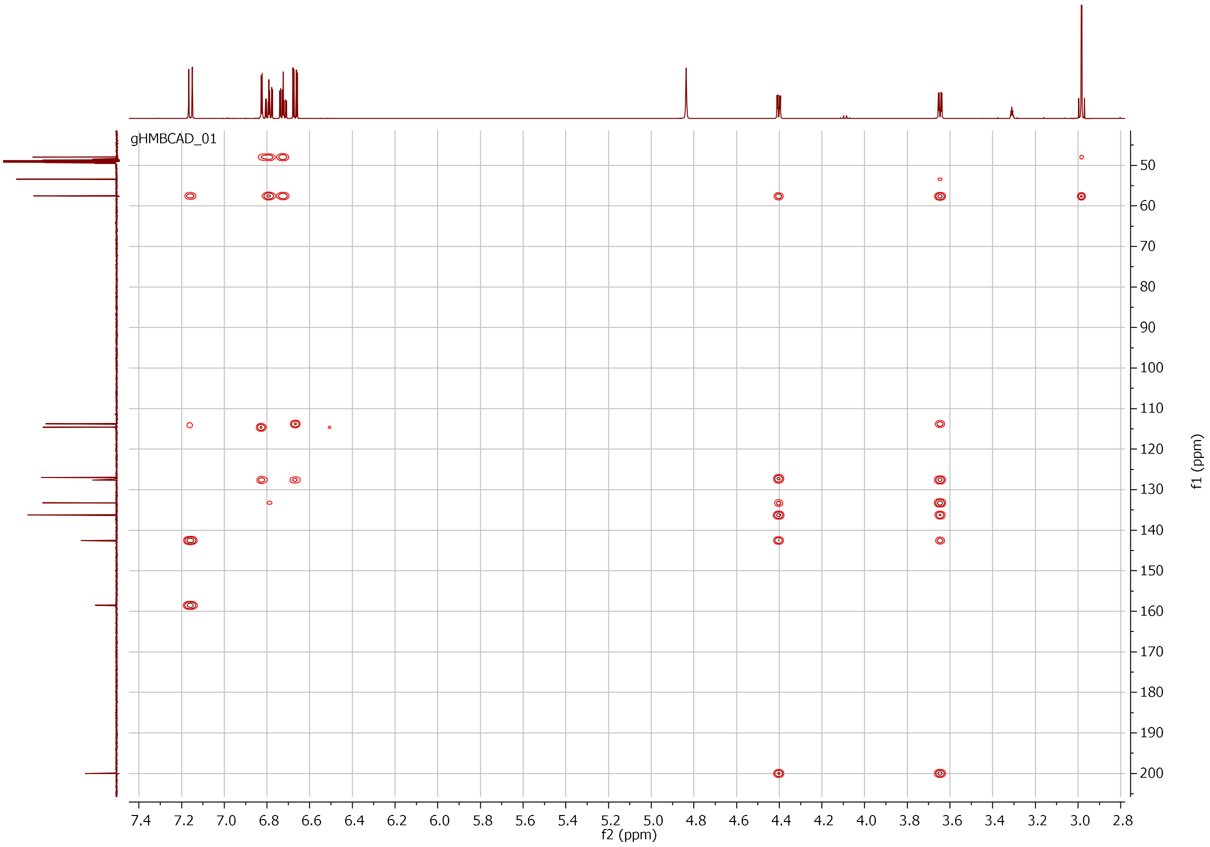
gHMBCAD_01
f2 (ppm)
f1 (ppm)

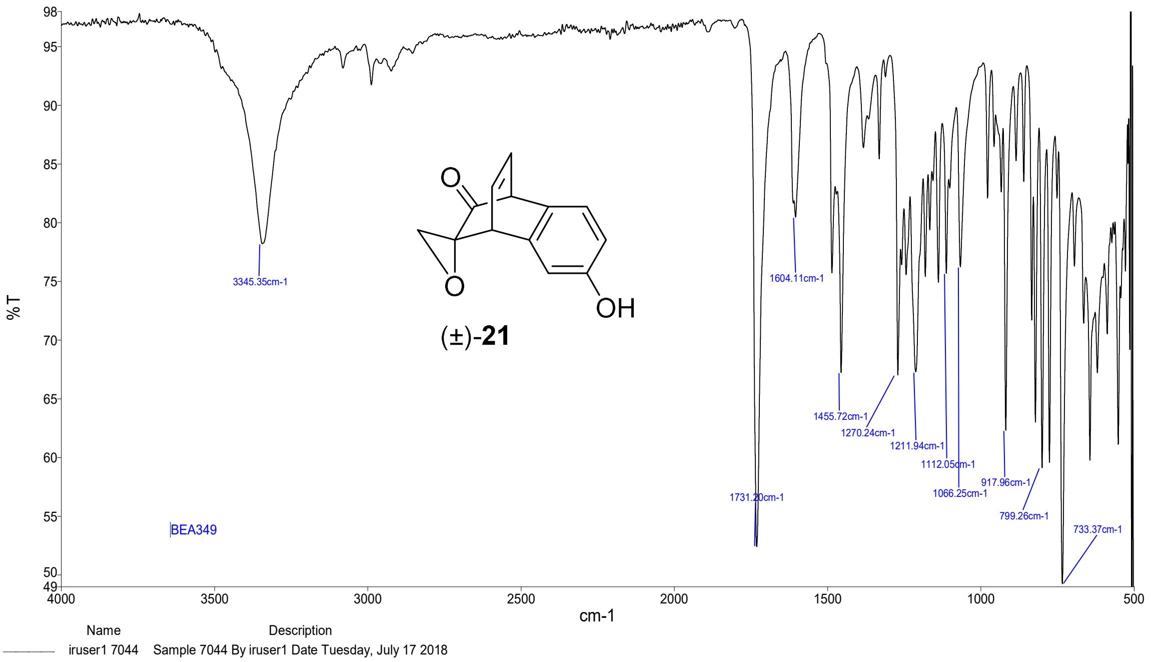

Confirmation of Expected Formula

Sample-ID	ba_sel_BEA349	Submitter	bea23 Ben Alexander
Analysis Name	ba_sel_BEA349_358364_45_01_64743.d	Supervisor	sl288 Simon Lewis
Method used	Confirm Formula Positive 50to500 loop inj.m	Acquisition Date	14/08/2018 11:42:12
Ionisation Mode	positive electrospray (ESI)		

+MS, 1.0-1.3min #(119-154), -Spectral Bkgrnd

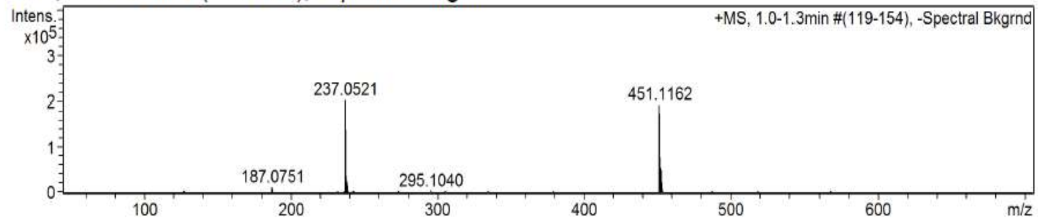

#	m/z	I	I %	Area	S/N
1	127.0881	3397	1.7	68	1457.2
2	187.0751	12673	6.2	414	1798.9
3	236.0763	4001	2.0	90	163.3
4	237.0521	203135	100.0	8609	8153.1
5	238.0564	23460	11.5	931	925.5
6	295.1040	4550	2.2	140	361.4
7	451.1162	192247	94.6	16339	6404.8
8	452.1194	54787	27.0	4380	1788.4
9	453.1225	9726	4.8	781	311.1
10	487.0950	3692	1.8	339	146.8

Generate Molecular Formula Parameters

Charge	Tolerance	SearchRadius	H/C Ratio min.	H/C Ratio max.	Electron Conf.	Nitrogen Rule	sigma limit
positive	25 ppm	0.05 m/z	0	3	both	true	0.05

Expected Formula C13 H10 O3 **Adduct(s):** H, Na

#	meas. m/z	theo. m/z	Err[ppm]	Sigma	Formula
1	237.0521	237.0522	-0.50	0.0154	C 13 H 10 Na 1 O 3

Note: Sigma fits < 0.05 indicates high probability of correct MF.

For formula confirmation the mass error / accuracy at 200 Da should be better than 25 ppm, for 500 Da better than 10 ppm and for 1000 Da better than 5 ppm

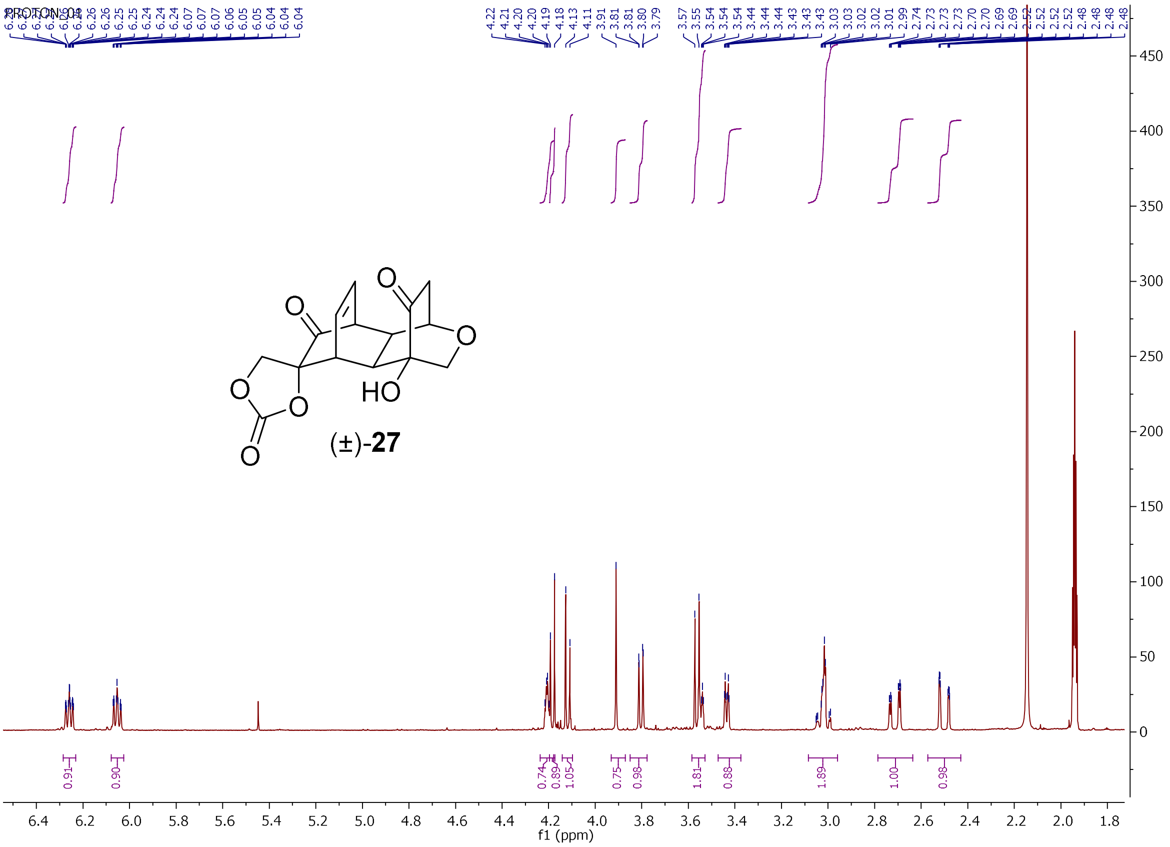
4.22 4.21 4.20 4.19 4.18 4.13 4.11 3.91 3.81 3.81 3.80 3.79
3.57 3.55 3.54 3.54 3.54 3.44 3.44 3.44 3.43 3.43 3.03 3.03 3.02 3.02 3.01 2.99 2.74 2.73 2.73 2.70 2.70 2.69 2.69 2.52 2.52 2.52 2.48 2.48 2.48
0.91
0.90
0.74
0.89
1.05
0.75
0.98
1.81
0.88
1.89
1.00
0.98
HO
(±)-27
f1 (ppm)

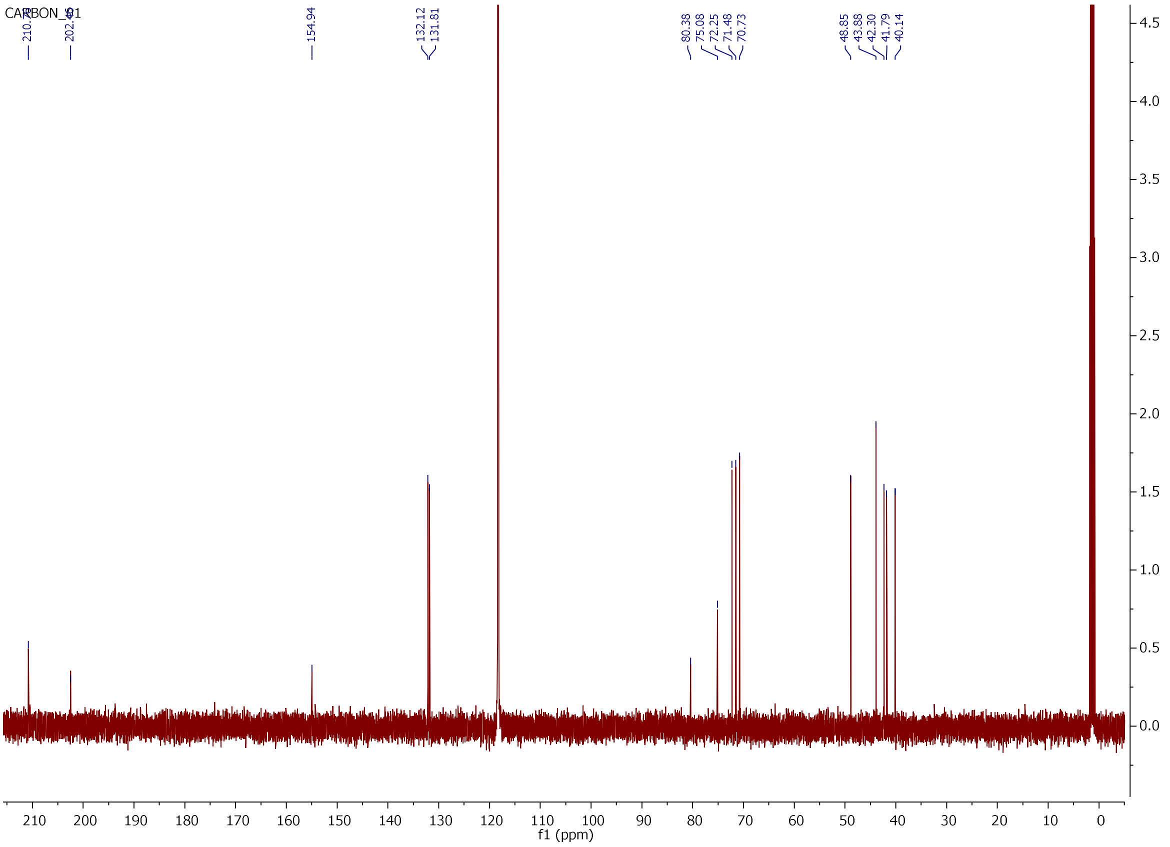
154.94
132.12
131.81
80.38
75.08
72.25
71.48
70.73
48.85
43.88
42.30
41.79
40.14
f1 (ppm)

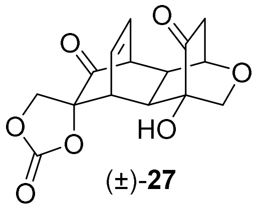

(±)-**27**

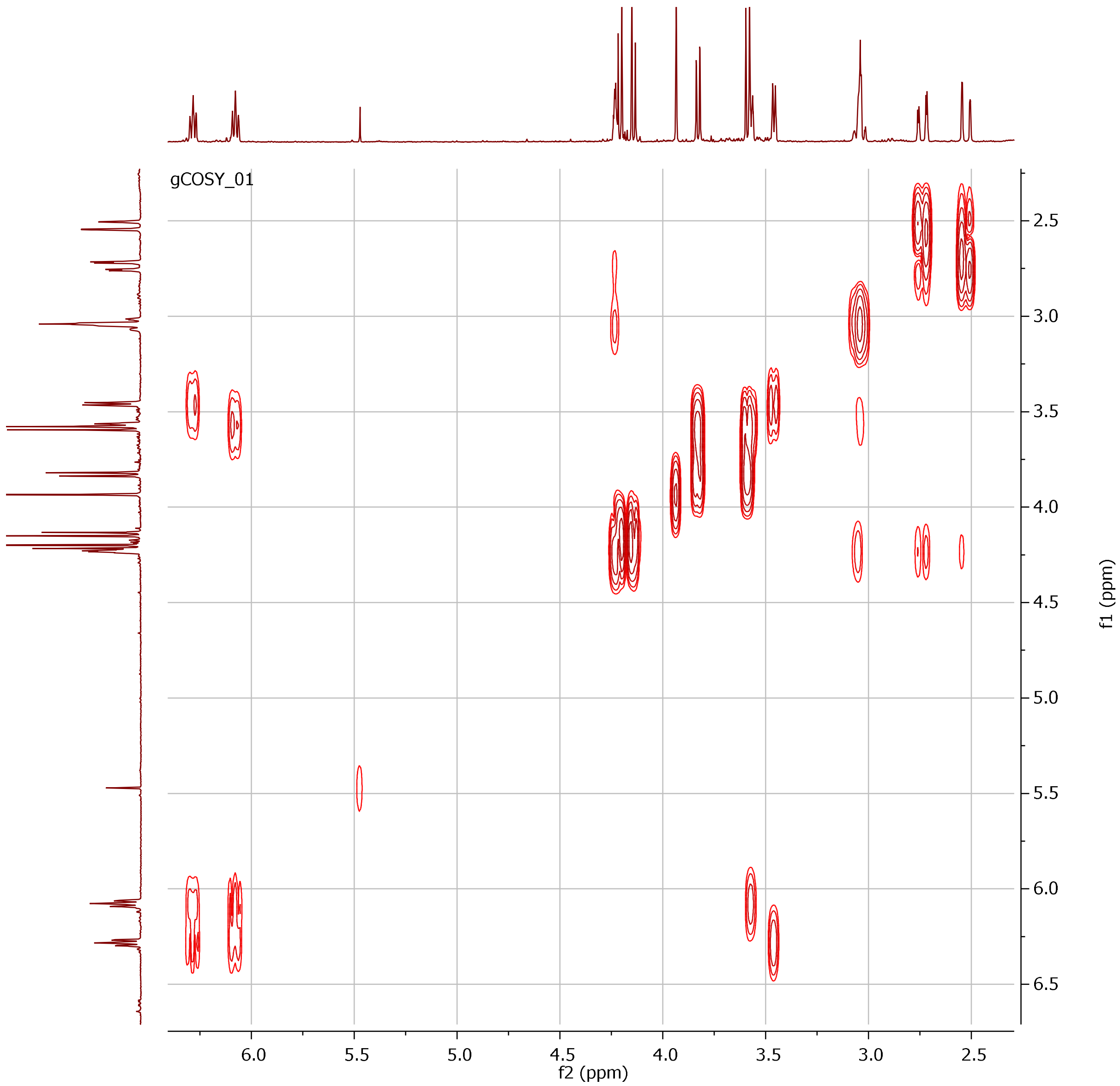

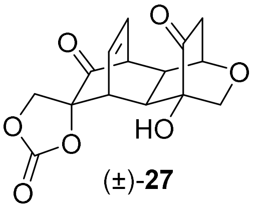

(±)-**27**

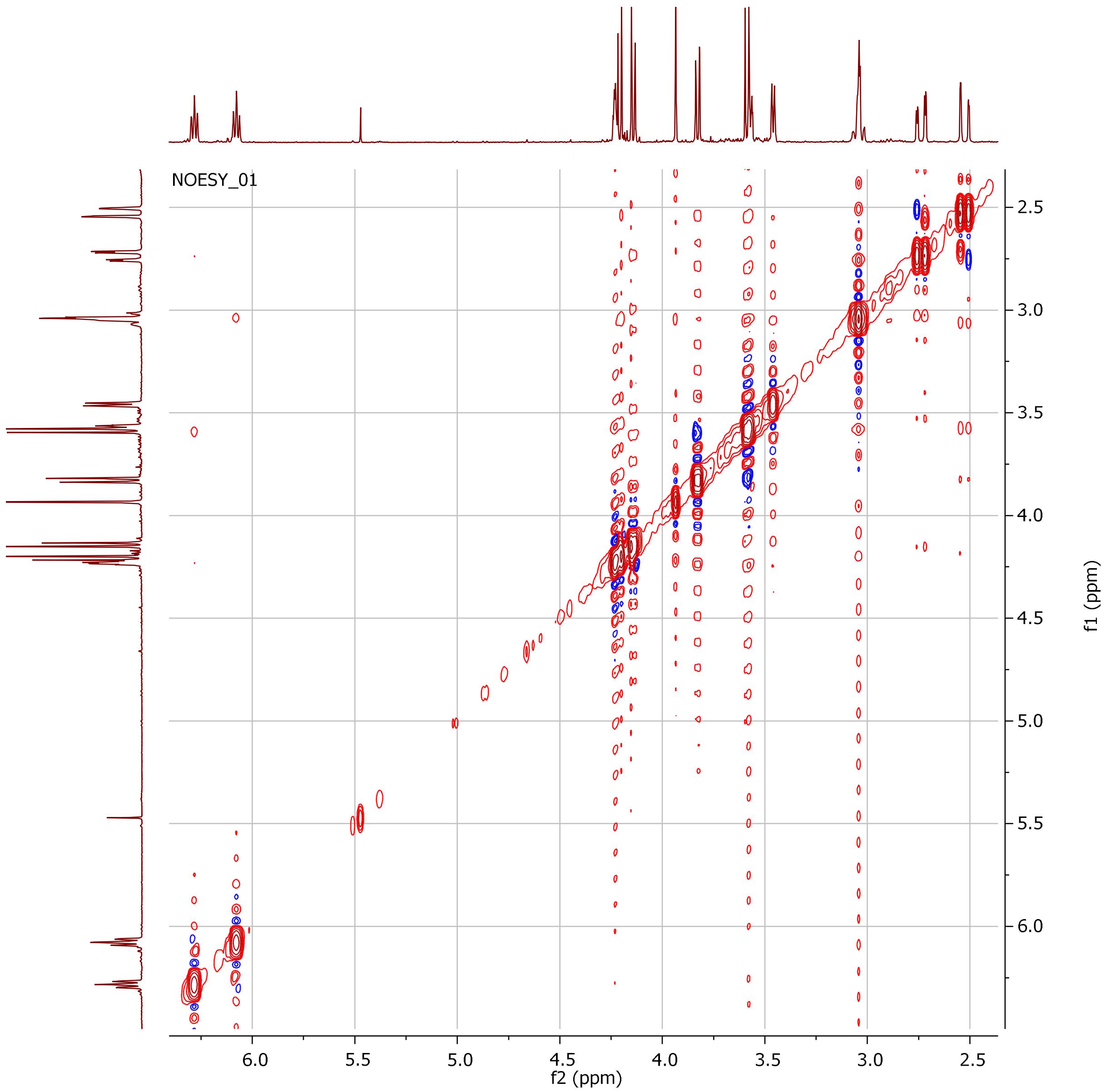

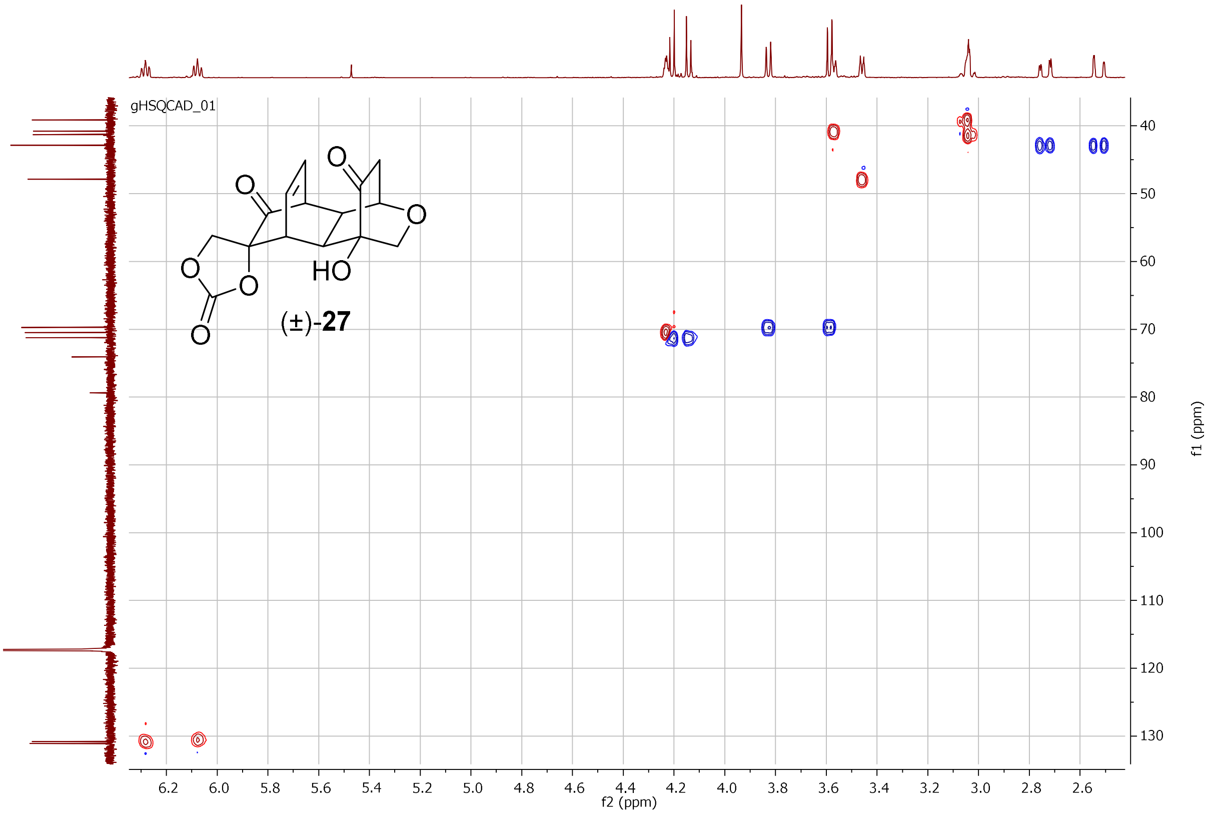
gHSQCAD_01
(±)-27
HO
f2 (ppm)
f1 (ppm)

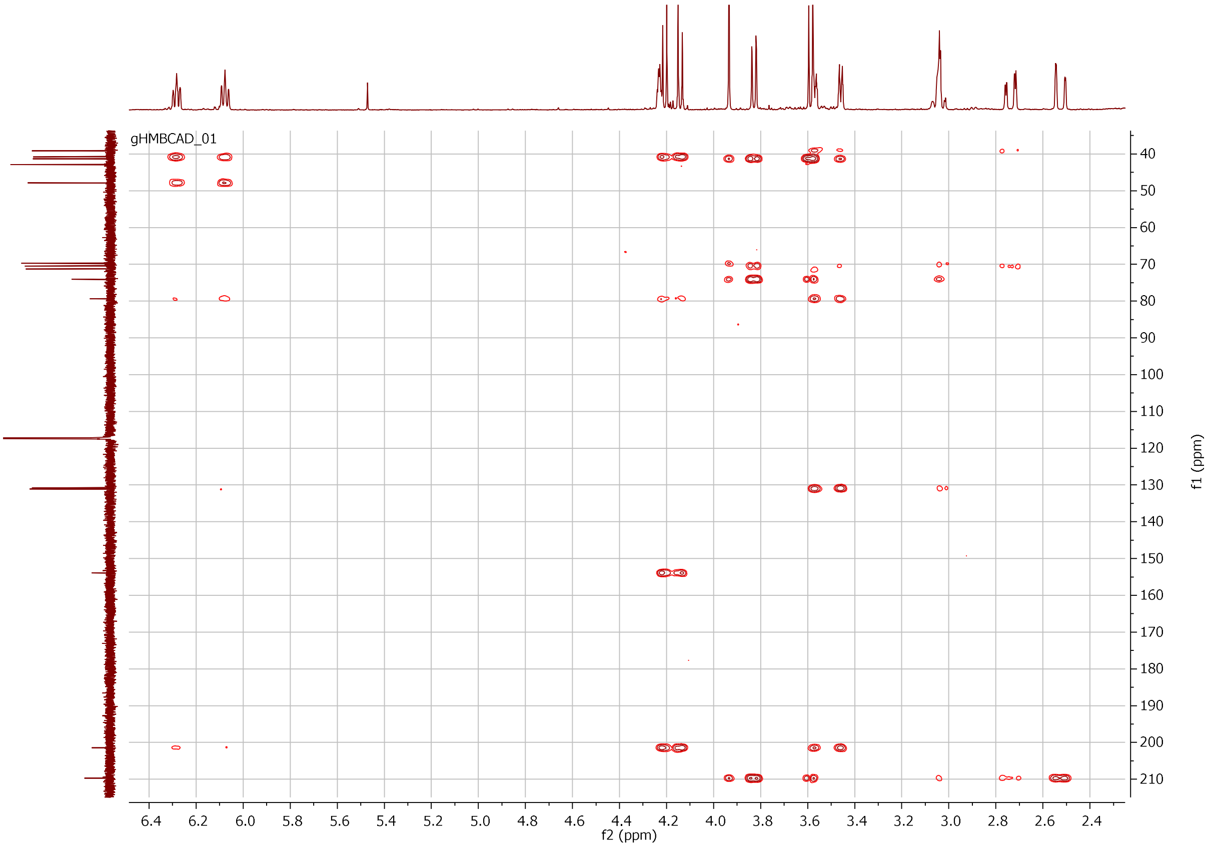
gHMBCAD_01
f2 (ppm)
f1 (ppm)

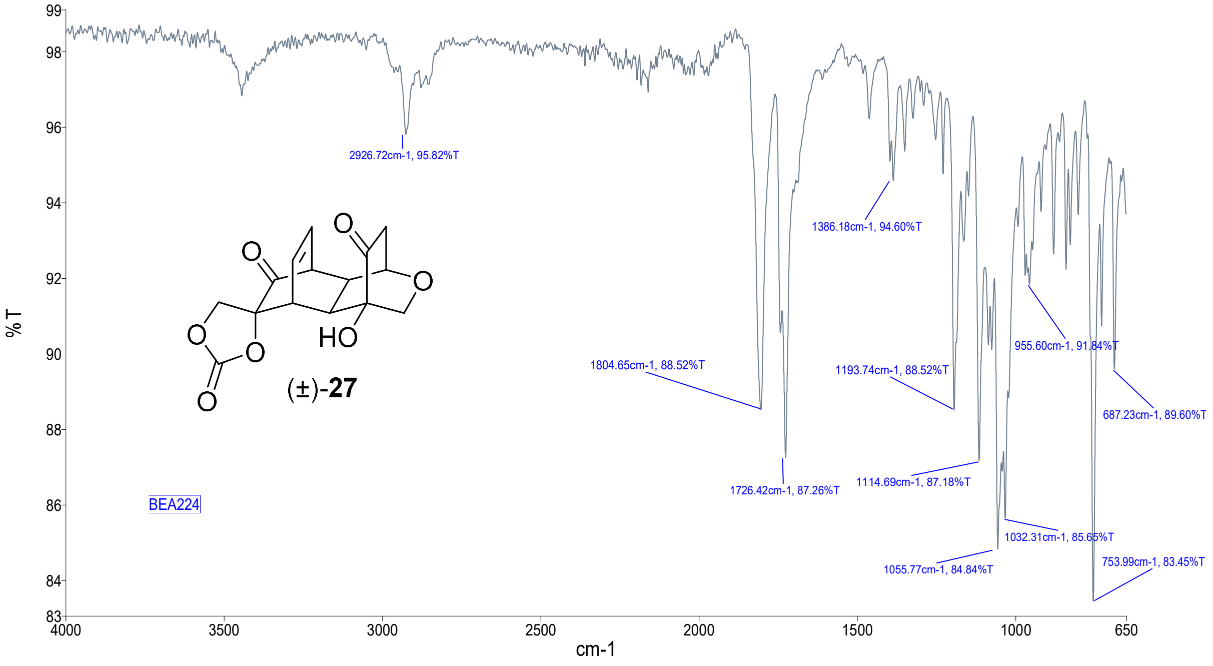

Mass Spectrum SmartFormula Report

Analysis Info

Analysis Name	Z:\ba_sel_BEA224_358358_39_01_64730.d	Acquisition Date	8/14/2018 10:47:18 AM
Method	Confirm Formula Negative 50to500 loop inj.m	Operator	admin
Sample Name	ba_sel_BEA224_358358	Instrument / Ser#	micrOTOF 161
Comment			

Acquisition Parameter

Source Type	ESI	Ion Polarity	Negative	Set Nebulizer	2.2 Bar
Focus	Not active			Set Dry Heater	220 °C
Scan Begin	50 m/z	Set Capillary	4500 V	Set Dry Gas	10.2 l/min
Scan End	750 m/z	Set End Plate Offset	-500 V	Set Divert Valve	Source

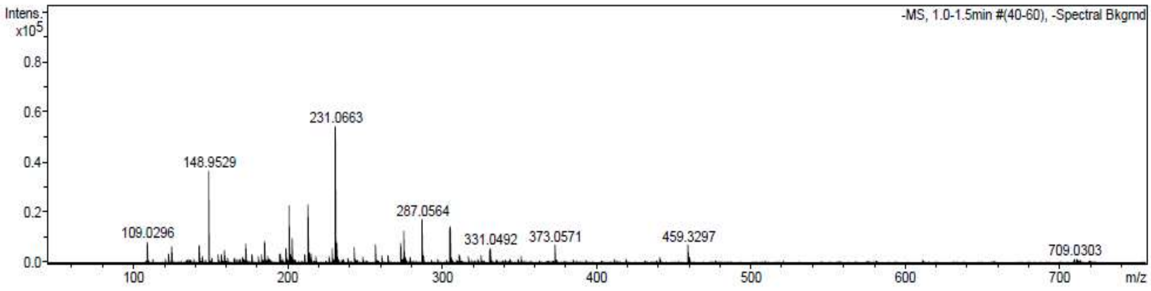

Meas. m/z	#	Formula	Score	m/z	err [mDa]	err [ppm]	mSigma	rdb	e⁻ Conf	N-Rule
287.0564	1	C 12 H 3 N 10	46.93	287.0548	-1.7	-5.8	4.4	16.5	even	ok
	2	C 15 H 11 O 6	100.00	287.0561	-0.3	-1.1	5.0	10.5	even	ok
	3	C 11 H 7 N 6 O 4	13.74	287.0534	-3.0	-10.4	11.0	11.5	even	ok
	4	C 16 H 7 N 4 O 2	56.26	287.0574	1.0	3.6	16.2	15.5	even	ok

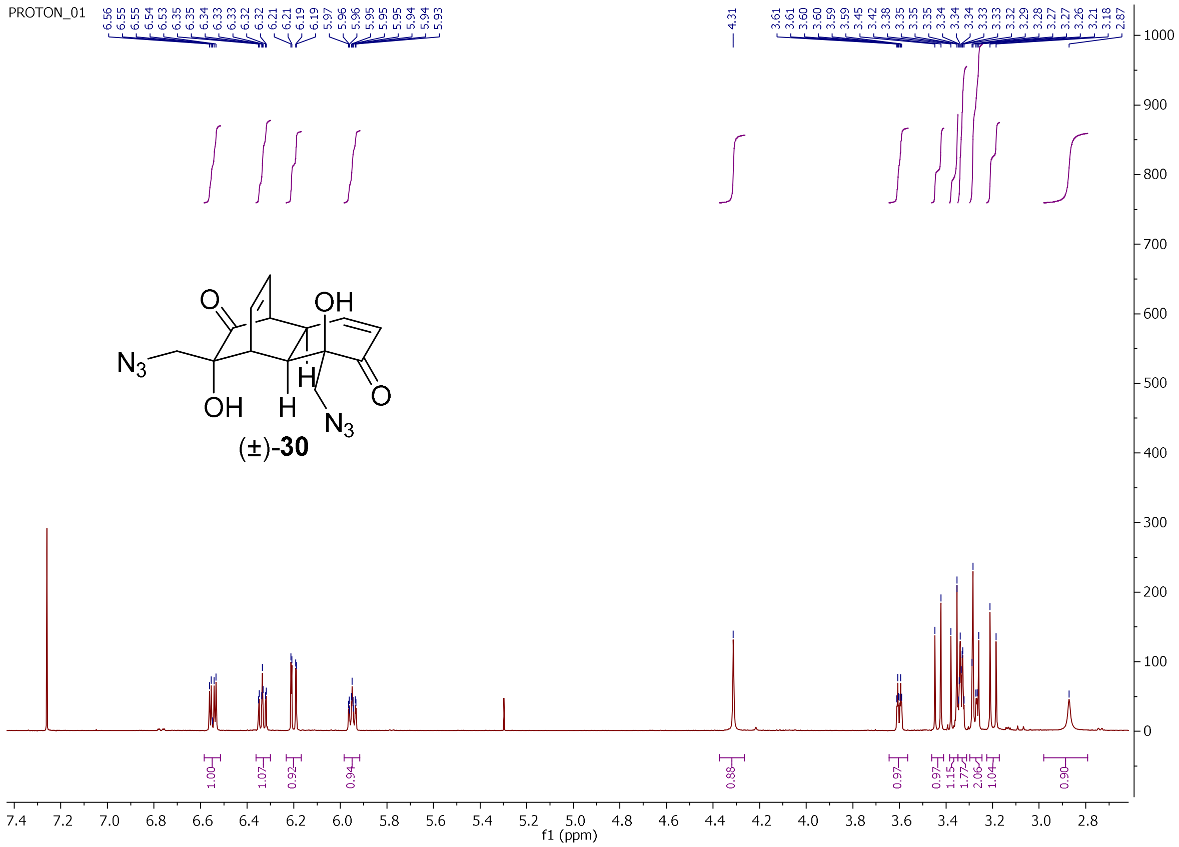
PROTON_01
6.56
6.55
6.55
6.54
6.53
6.35
6.35
6.34
6.33
6.33
6.32
6.32
6.21
6.21
6.19
6.19
5.97
5.96
5.96
5.95
5.95
5.95
5.94
5.94
5.93
4.31
3.61
3.61
3.60
3.60
3.59
3.59
3.45
3.42
3.38
3.35
3.35
3.35
3.34
3.34
3.34
3.33
3.33
3.32
3.29
3.28
3.27
3.27
3.26
3.21
3.18
2.87
1.00
1.07
0.92
0.94
0.88
0.97
0.97
1.15
1.77
2.06
1.04
0.90
(±)-30
f1 (ppm)

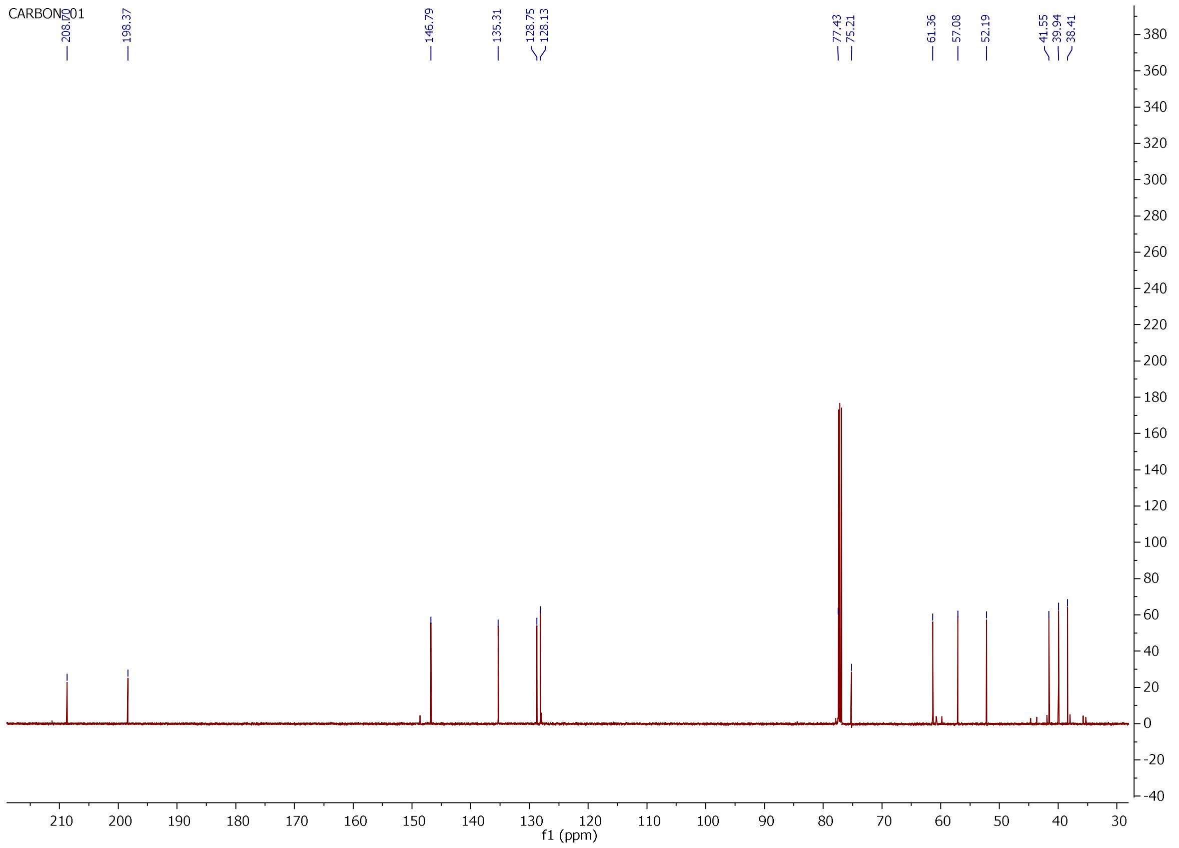
CARBON_01
208.70
198.37
146.79
135.31
128.75
128.13
77.43
75.21
61.36
57.08
52.19
41.55
39.94
38.41
f1 (ppm)

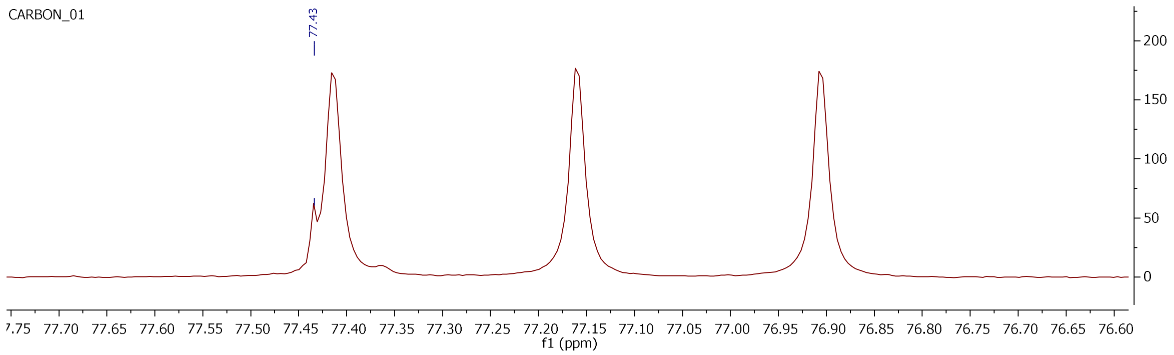

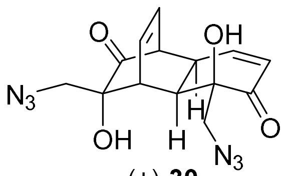

(±)-**30**

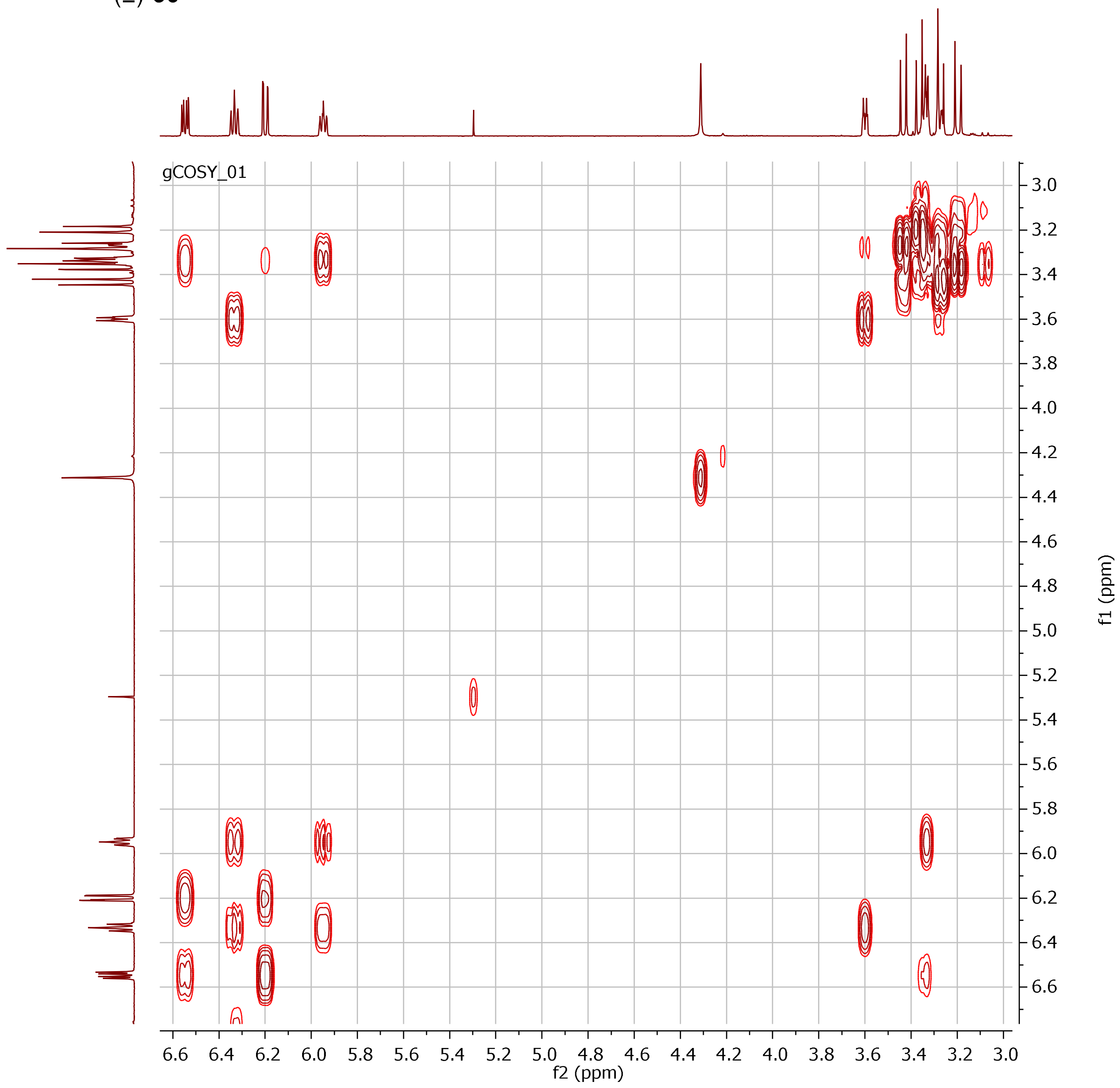

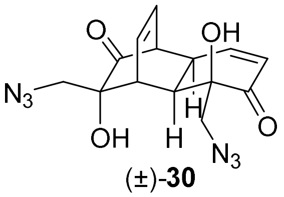

(±)-**30**

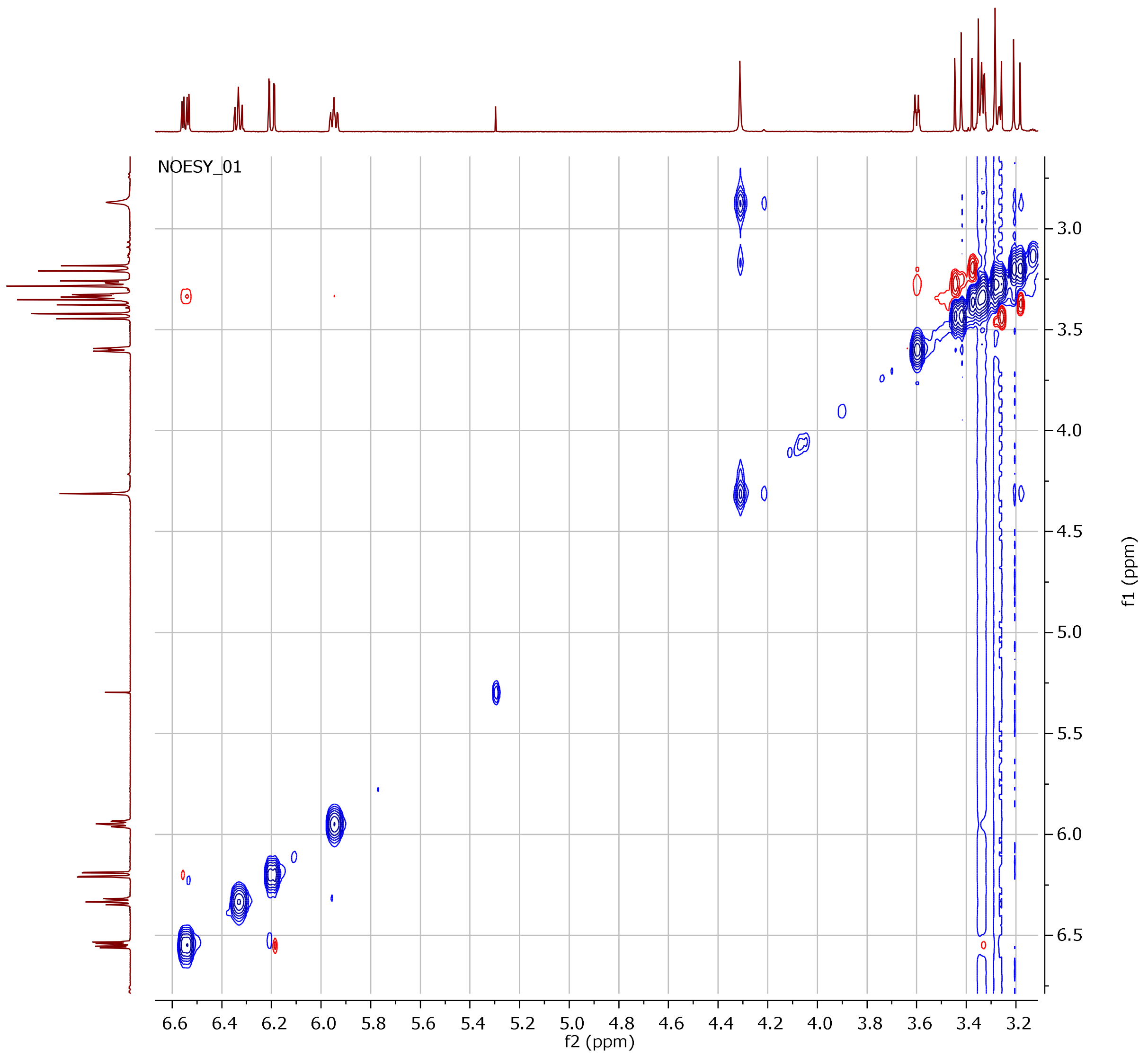

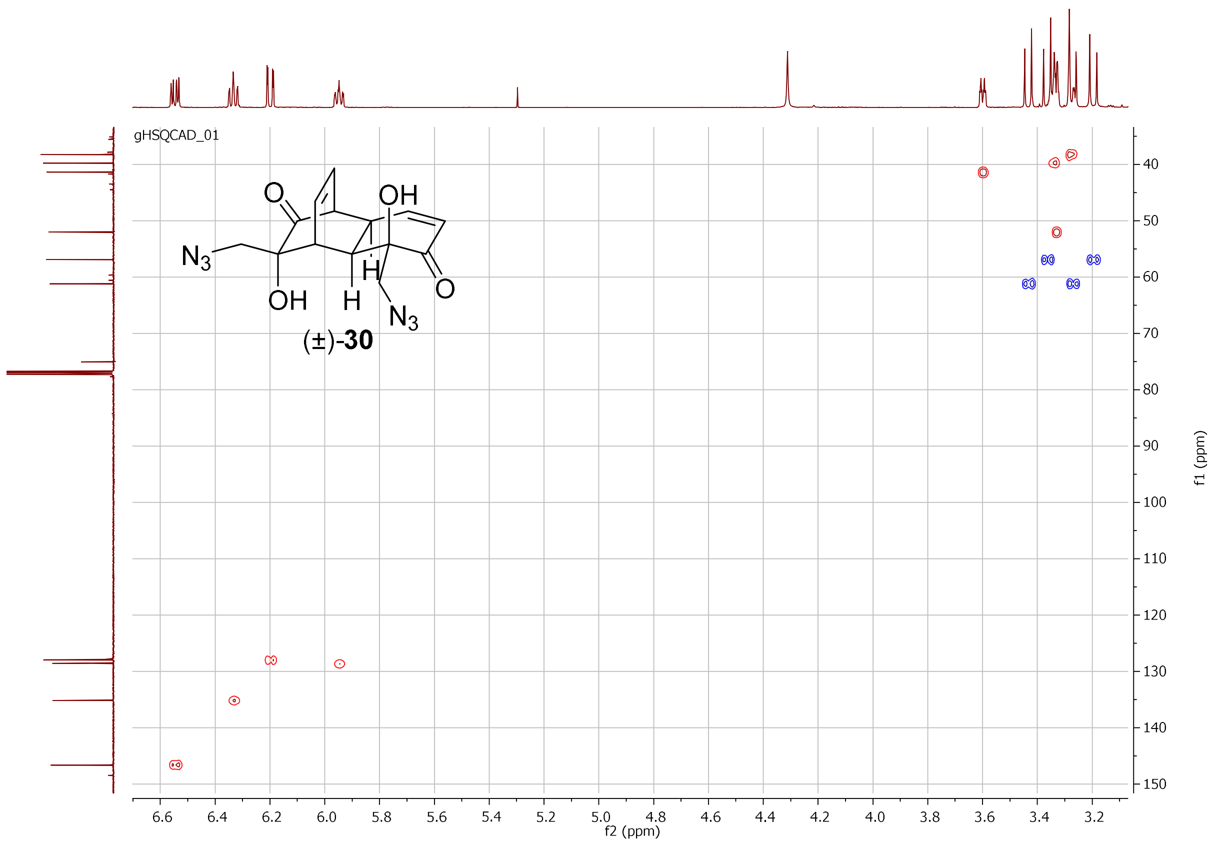
gHSQCAD_01
(±)-30
f2 (ppm)
f1 (ppm)

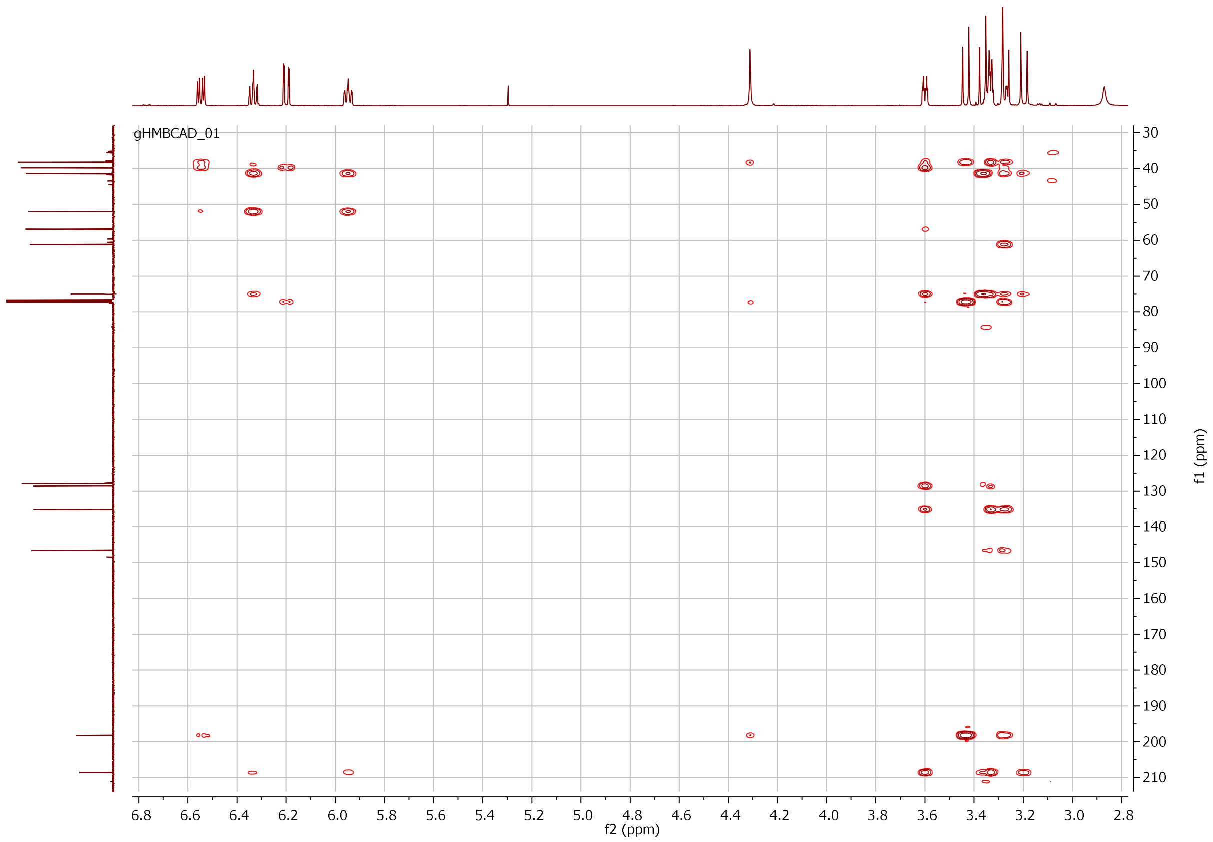
gHMBCAD_01
f2 (ppm)
f1 (ppm)

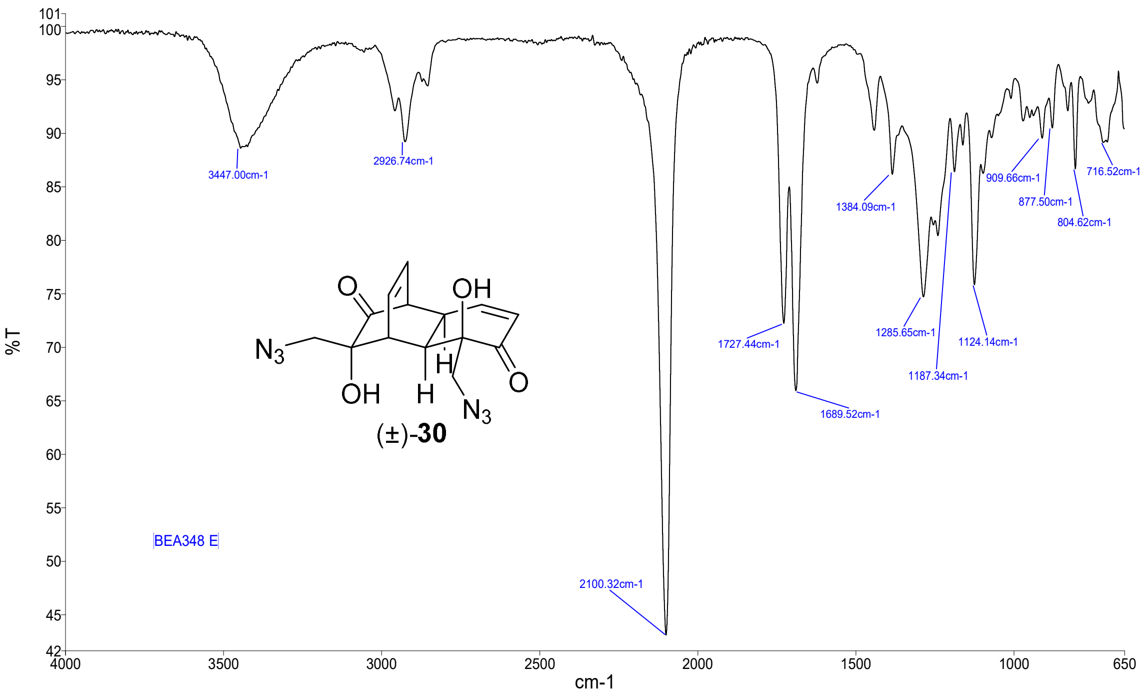

Name	Description
iruser1 6764	Sample 6764 By iruser1 Date Thursday, April 05 2018

Confirmation of Expected Formula

Sample-ID	ba_sel_BEA263D	Submitter	bea23 Ben Alexander
Analysis Name	ba_sel_BEA263D_353278_8_01_58883.d	Supervisor	sl288 Simon Lewis
Method used	Confirm Formula Positive 50to500 loop inj.m	Acquisition Date	12/09/2017 18:03:15
Ionisation Mode	positive electrospray (ESI)		

+MS, 1.0-1.3min #(119-154), -Spectral Bkgrnd

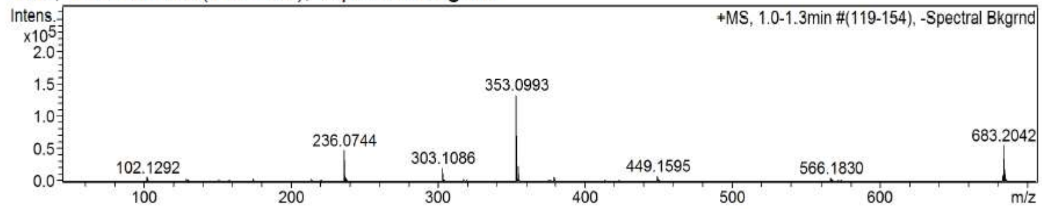

#	m/z	I	I %	Area	S/N
1	102.1292	6285	4.7	139	2896.6
2	236.0744	47576	35.7	1578	3023.7
3	237.0805	5476	4.1	183	341.0
4	303.1086	20746	15.6	1112	1453.3
5	353.0993	133337	100.0	8481	5291.2
6	354.1014	23455	17.6	1390	916.3
7	379.1155	5986	4.5	327	186.5
8	449.1595	8355	6.3	539	695.4
9	683.2042	55598	41.7	7196	3694.4
10	684.2082	19053	14.3	2462	1265.9

Generate Molecular Formula Parameters

Charge	Tolerance	SearchRadius	H/C Ratio min.	H/C Ratio max.	Electron Conf.	Nitrogen Rule	sigma limit
positive	10 ppm	0.05 m/z	0	3	both	true	0.05

Expected Formula C14 H14 N6 O4 **Adduct(s):** H, Na

#	meas. m/z	theo. m/z	Err[ppm]	Sigma	Formula
1	353.0993	353.0969	6.90	0.0043	C 14 H 14 N 6 Na 1 O 4

Note: Sigma fits < 0.05 indicates high probability of correct MF, and mass accuracy of 5ppm or better is generally acceptable for publication

Colony Count Assay – Statistical Analysis

Control 25 µM 50µM 100µM

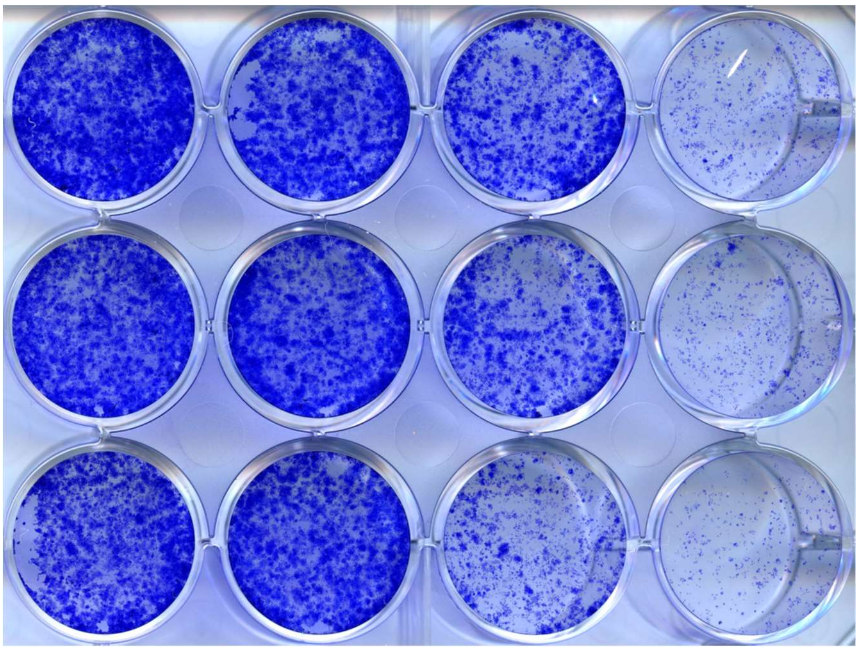

Column B	25 µM
vs.	vs.
Column A	Control
Unpaired t test	
P value	0.5056
P value summary	ns
Significantly different (P < 0.05)?	No
One- or two-tailed P value?	Two-tailed
t, df	t=0.7305 df=4

Column C	50 µM
vs.	vs.
Column A	Control
Unpaired t test	
P value	0.0150
P value summary	*
Significantly different (P < 0.05)?	Yes
One- or two-tailed P value?	Two-tailed
t, df	t=4.086 df=4

Column D	100 µM
vs.	vs.
Column A	Control
Unpaired t test	
P value	0.0007
P value summary	***
Significantly different (P < 0.05)?	Yes
One- or two-tailed P value?	Two-tailed
t, df	t=9.538 df=4

Column C	50 µM
vs.	vs.
Column B	25 µM
Unpaired t test	
P value	0.0084
P value summary	**
Significantly different (P < 0.05)?	Yes
One- or two-tailed P value?	Two-tailed
t, df	t=4.843 df=4

Column D	100 µM
vs.	vs.
Column B	25 µM
Unpaired t test	
P value	<0.0001
P value summary	****
Significantly different (P < 0.05)?	Yes
One- or two-tailed P value?	Two-tailed
t, df	t=87.54 df=4

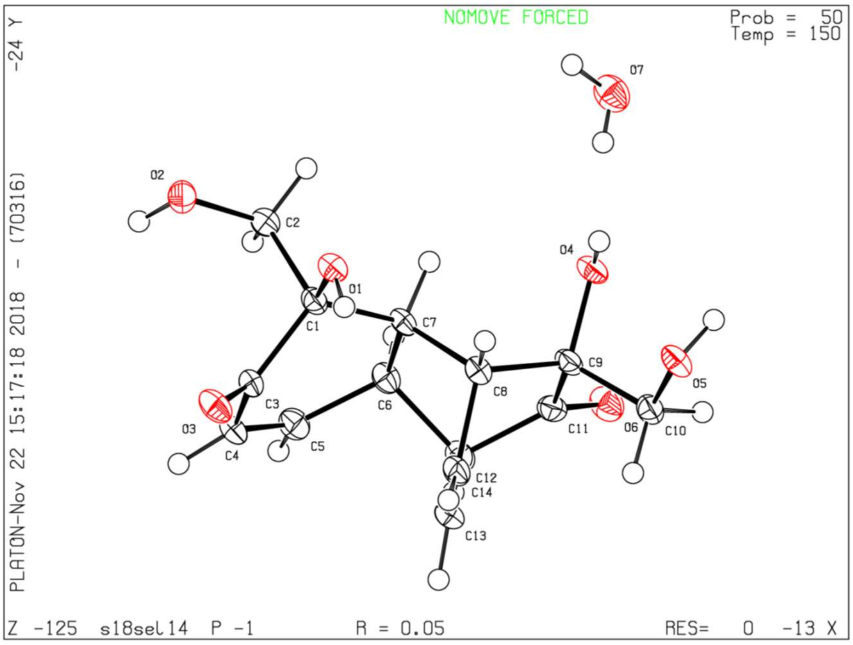

Table S1. Crystal data and structure refinement for (±)-**11**.

CCDC number	1952565	
Empirical formula	$C_{14}H_{18}O_7$	
Formula weight	298.28	
Temperature	150.00(10) K	
Wavelength	1.54184 Å	
Crystal system	Triclinic	
Space group	P-1	
Unit cell dimensions	a = 8.0962(7) Å	α= 75.974(7)°.
	b = 8.8914(7) Å	β= 83.677(7)°.
	c = 9.7024(7) Å	γ = 76.239(7)°.
Volume	657.15(9) $Å^3$	
Z	2	
Density (calculated)	1.507 Mg/m^3	
Absorption coefficient	1.034 mm^{-1}	
F(000)	316	
Crystal size	0.203 x 0.103 x 0.072 mm^3	
Theta range for data collection	4.705 to 72.876°.	
Index ranges	-8<=h<=10, -11<=k<=10, -11<=l<=9	
Reflections collected	4762	
Independent reflections	2570 [R(int) = 0.0369]	
Completeness to theta = 67.684°	99.7 %	
Absorption correction	Semi-empirical from equivalents	
Max. and min. transmission	1.00000 and 0.75501	
Refinement method	Full-matrix least-squares on F^2	
Data / restraints / parameters	2570 / 0 / 212	
Goodness-of-fit on F^2	1.034	
Final R indices [I>2sigma(I)]	R1 = 0.0530, wR2 = 0.1462	
R indices (all data)	R1 = 0.0580, wR2 = 0.1557	
Extinction coefficient	n/a	
Largest diff. peak and hole	0.463 and -0.352 e.$Å^{-3}$	

Table S2. Atomic coordinates (x 10^4) and equivalent isotropic displacement parameters ($Å^2$x 10^3) for (±)-**11**. U(eq) is defined as one third of the trace of the orthogonalized U^{ij} tensor.

	x	y	z	U(eq)
C(1)	3732(2)	7359(2)	3736(2)	17(1)
C(2)	5669(2)	7269(2)	3625(2)	20(1)
C(3)	2807(2)	9082(2)	3260(2)	18(1)
C(4)	3011(2)	9804(2)	1738(2)	21(1)
C(5)	3378(2)	8890(2)	788(2)	22(1)
C(6)	3482(2)	7126(2)	1144(2)	19(1)
C(7)	3408(2)	6316(2)	2760(2)	17(1)
C(8)	1718(2)	5734(2)	3156(2)	17(1)
C(9)	1841(2)	4373(2)	2376(2)	18(1)
C(10)	233(2)	3693(2)	2626(2)	20(1)
C(11)	2092(2)	5081(2)	776(2)	19(1)
C(12)	1949(2)	6863(2)	433(2)	21(1)
C(13)	333(2)	7604(2)	1190(2)	22(1)
C(14)	239(2)	7049(2)	2594(2)	21(1)
O(1)	3309(2)	6760(1)	5183(1)	19(1)
O(2)	6133(2)	8190(2)	4462(1)	23(1)
O(3)	1998(2)	9812(1)	4128(1)	24(1)
O(4)	3279(2)	3113(1)	2745(1)	20(1)
O(5)	71(2)	2830(1)	4060(1)	21(1)
O(6)	2421(2)	4266(2)	-91(1)	25(1)
O(7)	6576(2)	1078(2)	2863(2)	28(1)

Table S3. Bond lengths [Å] for (±)-**11**.

C(1)-O(1)	1.4105(19)	C(8)-H(8)	1.0000
C(1)-C(3)	1.520(2)	C(9)-O(4)	1.4203(19)
C(1)-C(2)	1.544(2)	C(9)-C(10)	1.533(2)
C(1)-C(7)	1.556(2)	C(9)-C(11)	1.538(2)
C(2)-O(2)	1.417(2)	C(10)-O(5)	1.4257(19)
C(2)-H(2A)	0.9900	C(10)-H(10A)	0.9900
C(2)-H(2B)	0.9900	C(10)-H(10B)	0.9900
C(3)-O(3)	1.225(2)	C(11)-O(6)	1.208(2)
C(3)-C(4)	1.469(2)	C(11)-C(12)	1.516(2)
C(4)-C(5)	1.336(2)	C(12)-C(13)	1.516(2)
C(4)-H(4)	0.9500	C(12)-H(12)	1.0000
C(5)-C(6)	1.506(2)	C(13)-C(14)	1.332(3)
C(5)-H(5)	0.9500	C(13)-H(13)	0.9500
C(6)-C(7)	1.560(2)	C(14)-H(14)	0.9500
C(6)-C(12)	1.573(2)	O(1)-H(1)	0.75(3)
C(6)-H(6)	1.0000	O(2)-H(2)	0.86(3)
C(7)-C(8)	1.554(2)	O(4)-H(4A)	0.84(3)
C(7)-H(7)	1.0000	O(5)-H(5A)	0.84(3)
C(8)-C(14)	1.506(2)	O(7)-H(7A)	0.85(4)
C(8)-C(9)	1.554(2)	O(7)-H(7B)	0.74(4)

Table S4. Bond angles [°] for (±)-**11**.

O(1)-C(1)-C(3)	110.63(13)	C(14)-C(8)-H(8)	110.9
O(1)-C(1)-C(2)	106.11(13)	C(7)-C(8)-H(8)	110.9
C(3)-C(1)-C(2)	109.00(13)	C(9)-C(8)-H(8)	110.9
O(1)-C(1)-C(7)	112.36(13)	O(4)-C(9)-C(10)	109.03(13)
C(3)-C(1)-C(7)	111.67(13)	O(4)-C(9)-C(11)	106.45(12)
C(2)-C(1)-C(7)	106.79(12)	C(10)-C(9)-C(11)	108.65(13)
O(2)-C(2)-C(1)	112.39(13)	O(4)-C(9)-C(8)	112.55(13)
O(2)-C(2)-H(2A)	109.1	C(10)-C(9)-C(8)	112.94(13)
C(1)-C(2)-H(2A)	109.1	C(11)-C(9)-C(8)	106.92(13)
O(2)-C(2)-H(2B)	109.1	O(5)-C(10)-C(9)	110.84(13)
C(1)-C(2)-H(2B)	109.1	O(5)-C(10)-H(10A)	109.5
H(2A)-C(2)-H(2B)	107.9	C(9)-C(10)-H(10A)	109.5
O(3)-C(3)-C(4)	123.36(15)	O(5)-C(10)-H(10B)	109.5
O(3)-C(3)-C(1)	120.51(15)	C(9)-C(10)-H(10B)	109.5
C(4)-C(3)-C(1)	116.07(14)	H(10A)-C(10)-H(10B)	108.1
C(5)-C(4)-C(3)	120.07(15)	O(6)-C(11)-C(12)	124.71(15)
C(5)-C(4)-H(4)	120.0	O(6)-C(11)-C(9)	122.14(15)
C(3)-C(4)-H(4)	120.0	C(12)-C(11)-C(9)	113.12(13)
C(4)-C(5)-C(6)	124.49(15)	C(13)-C(12)-C(11)	107.91(14)
C(4)-C(5)-H(5)	117.8	C(13)-C(12)-C(6)	106.81(13)
C(6)-C(5)-H(5)	117.8	C(11)-C(12)-C(6)	105.81(13)
C(5)-C(6)-C(7)	116.01(13)	C(13)-C(12)-H(12)	112.0
C(5)-C(6)-C(12)	106.46(14)	C(11)-C(12)-H(12)	112.0
C(7)-C(6)-C(12)	109.05(13)	C(6)-C(12)-H(12)	112.0
C(5)-C(6)-H(6)	108.4	C(14)-C(13)-C(12)	114.70(15)
C(7)-C(6)-H(6)	108.4	C(14)-C(13)-H(13)	122.6
C(12)-C(6)-H(6)	108.4	C(12)-C(13)-H(13)	122.6
C(8)-C(7)-C(1)	113.76(12)	C(13)-C(14)-C(8)	114.52(14)
C(8)-C(7)-C(6)	109.18(13)	C(13)-C(14)-H(14)	122.7
C(1)-C(7)-C(6)	113.55(13)	C(8)-C(14)-H(14)	122.7
C(8)-C(7)-H(7)	106.6	C(1)-O(1)-H(1)	109.5
C(1)-C(7)-H(7)	106.6	C(2)-O(2)-H(2)	107(2)
C(6)-C(7)-H(7)	106.6	C(9)-O(4)-H(4A)	109.3(19)
C(14)-C(8)-C(7)	109.96(13)	C(10)-O(5)-H(5A)	107.4(16)
C(14)-C(8)-C(9)	107.68(13)	H(7A)-O(7)-H(7B)	104(3)
C(7)-C(8)-C(9)	106.41(13)		

Table S5. Anisotropic displacement parameters ($Å^2$x 10^3) for (±)-**11**. The anisotropic displacement factor exponent takes the form: $-2\pi^2[h^2 a^{*2}U^{11} + ... + 2 h k a^* b^* U^{12}]$

	U^{11}	U^{22}	U^{33}	U^{23}	U^{13}	U^{12}
C(1)	19(1)	12(1)	19(1)	-4(1)	-2(1)	-2(1)
C(2)	19(1)	16(1)	24(1)	-6(1)	-2(1)	-1(1)
C(3)	18(1)	12(1)	23(1)	-5(1)	-3(1)	-2(1)
C(4)	24(1)	11(1)	25(1)	-1(1)	-3(1)	-2(1)
C(5)	24(1)	18(1)	20(1)	-1(1)	0(1)	-5(1)
C(6)	23(1)	15(1)	19(1)	-5(1)	1(1)	-3(1)
C(7)	19(1)	11(1)	19(1)	-4(1)	0(1)	0(1)
C(8)	19(1)	14(1)	19(1)	-4(1)	0(1)	-2(1)
C(9)	19(1)	12(1)	21(1)	-4(1)	-3(1)	1(1)
C(10)	23(1)	14(1)	21(1)	-4(1)	-3(1)	-3(1)
C(11)	18(1)	17(1)	21(1)	-4(1)	-3(1)	0(1)
C(12)	27(1)	16(1)	19(1)	-2(1)	-3(1)	-3(1)
C(13)	23(1)	14(1)	29(1)	-4(1)	-7(1)	1(1)
C(14)	19(1)	14(1)	29(1)	-8(1)	-1(1)	-1(1)
O(1)	21(1)	15(1)	19(1)	-3(1)	-1(1)	-2(1)
O(2)	28(1)	19(1)	24(1)	-3(1)	-6(1)	-8(1)
O(3)	29(1)	16(1)	25(1)	-7(1)	1(1)	2(1)
O(4)	23(1)	13(1)	21(1)	-3(1)	-3(1)	2(1)
O(5)	24(1)	13(1)	24(1)	-3(1)	2(1)	0(1)
O(6)	32(1)	21(1)	22(1)	-9(1)	-1(1)	-1(1)
O(7)	35(1)	24(1)	25(1)	-8(1)	0(1)	-3(1)

Table S6. Hydrogen coordinates (x 10^4) and isotropic displacement parameters ($Å^2$x 10^3) for (±)-**11**.

	x	y	z	U(eq)
H(2A)	6274	6147	3939	24
H(2B)	6036	7647	2618	24
H(4)	2883	10922	1428	25
H(5)	3588	9387	-178	26
H(6)	4568	6601	694	23
H(7)	4352	5345	2897	20
H(8)	1560	5345	4209	21
H(10A)	-781	4572	2417	23
H(10B)	290	2981	1972	23
H(12)	1972	7322	-615	25
H(13)	-544	8401	701	27
H(14)	-686	7451	3193	25
H(1)	2370(30)	6990(30)	5323(8)	29
H(2)	6290(40)	9050(40)	3900(30)	51(8)
H(4A)	3220(40)	2730(30)	3620(30)	38(7)
H(5A)	630(30)	1890(30)	4100(30)	34(6)
H(7A)	6940(40)	1350(40)	3540(40)	60(9)
H(7B)	5790(50)	1690(40)	2670(30)	54(10)

Table S7. Torsion angles [°] for (±)-**11**.

O(1)-C(1)-C(2)-O(2)	-60.58(16)	C(14)-C(8)-C(9)-O(4)	175.47(13)
C(3)-C(1)-C(2)-O(2)	58.58(17)	C(7)-C(8)-C(9)-O(4)	57.60(17)
C(7)-C(1)-C(2)-O(2)	179.37(13)	C(14)-C(8)-C(9)-C(10)	-60.53(17)
O(1)-C(1)-C(3)-O(3)	8.1(2)	C(7)-C(8)-C(9)-C(10)	-178.39(13)
C(2)-C(1)-C(3)-O(3)	-108.23(18)	C(14)-C(8)-C(9)-C(11)	58.92(17)
C(7)-C(1)-C(3)-O(3)	134.03(16)	C(7)-C(8)-C(9)-C(11)	-58.94(15)
O(1)-C(1)-C(3)-C(4)	-174.45(14)	O(4)-C(9)-C(10)-O(5)	56.35(16)
C(2)-C(1)-C(3)-C(4)	69.24(18)	C(11)-C(9)-C(10)-O(5)	171.99(12)
C(7)-C(1)-C(3)-C(4)	-48.5(2)	C(8)-C(9)-C(10)-O(5)	-69.56(16)
O(3)-C(3)-C(4)-C(5)	-157.28(18)	O(4)-C(9)-C(11)-O(6)	51.0(2)
C(1)-C(3)-C(4)-C(5)	25.3(2)	C(10)-C(9)-C(11)-O(6)	-66.28(19)
C(3)-C(4)-C(5)-C(6)	4.7(3)	C(8)-C(9)-C(11)-O(6)	171.53(16)
C(4)-C(5)-C(6)-C(7)	-9.1(3)	O(4)-C(9)-C(11)-C(12)	-126.87(14)
C(4)-C(5)-C(6)-C(12)	112.44(19)	C(10)-C(9)-C(11)-C(12)	115.83(15)
O(1)-C(1)-C(7)-C(8)	41.77(18)	C(8)-C(9)-C(11)-C(12)	-6.36(18)
C(3)-C(1)-C(7)-C(8)	-83.21(17)	O(6)-C(11)-C(12)-C(13)	132.95(17)
C(2)-C(1)-C(7)-C(8)	157.72(13)	C(9)-C(11)-C(12)-C(13)	-49.23(18)
O(1)-C(1)-C(7)-C(6)	167.46(13)	O(6)-C(11)-C(12)-C(6)	-113.01(18)
C(3)-C(1)-C(7)-C(6)	42.48(18)	C(9)-C(11)-C(12)-C(6)	64.81(16)
C(2)-C(1)-C(7)-C(6)	-76.59(16)	C(5)-C(6)-C(12)-C(13)	-66.30(16)
C(5)-C(6)-C(7)-C(8)	112.36(15)	C(7)-C(6)-C(12)-C(13)	59.55(16)
C(12)-C(6)-C(7)-C(8)	-7.77(16)	C(5)-C(6)-C(12)-C(11)	178.90(12)
C(5)-C(6)-C(7)-C(1)	-15.7(2)	C(7)-C(6)-C(12)-C(11)	-55.24(16)
C(12)-C(6)-C(7)-C(1)	-135.86(14)	C(11)-C(12)-C(13)-C(14)	57.0(2)
C(1)-C(7)-C(8)-C(14)	78.81(16)	C(6)-C(12)-C(13)-C(14)	-56.4(2)
C(6)-C(7)-C(8)-C(14)	-49.17(16)	C(12)-C(13)-C(14)-C(8)	-2.9(2)
C(1)-C(7)-C(8)-C(9)	-164.85(13)	C(7)-C(8)-C(14)-C(13)	58.67(19)
C(6)-C(7)-C(8)-C(9)	67.18(15)	C(9)-C(8)-C(14)-C(13)	-56.87(19)

Table S8. Hydrogen bonds for (±)-**11** [Å and °].

D-H...A	d(D-H)	d(H...A)	d(D...A)	<(DHA)
O(1)-H(1)...O(5)#1	0.75	1.98	2.7152(18)	168.5
O(2)-H(2)...O(7)#2	0.86(3)	1.89(3)	2.741(2)	172(3)
O(4)-H(4A)...O(2)#3	0.84(3)	1.92(3)	2.7231(17)	161(3)
O(5)-H(5A)...O(3)#4	0.84(3)	1.91(3)	2.7545(17)	177(3)
O(7)-H(7A)...O(1)#3	0.85(4)	2.28(4)	3.0316(18)	147(3)
O(7)-H(7A)...O(3)#3	0.85(4)	2.41(4)	3.110(2)	140(3)
O(7)-H(7B)...O(4)	0.74(4)	2.13(4)	2.842(2)	163(3)

Symmetry transformations used to generate equivalent atoms:
#1 -x,-y+1,-z+1 #2 x,y+1,z #3 -x+1,-y+1,-z+1
#4 x,y-1,z

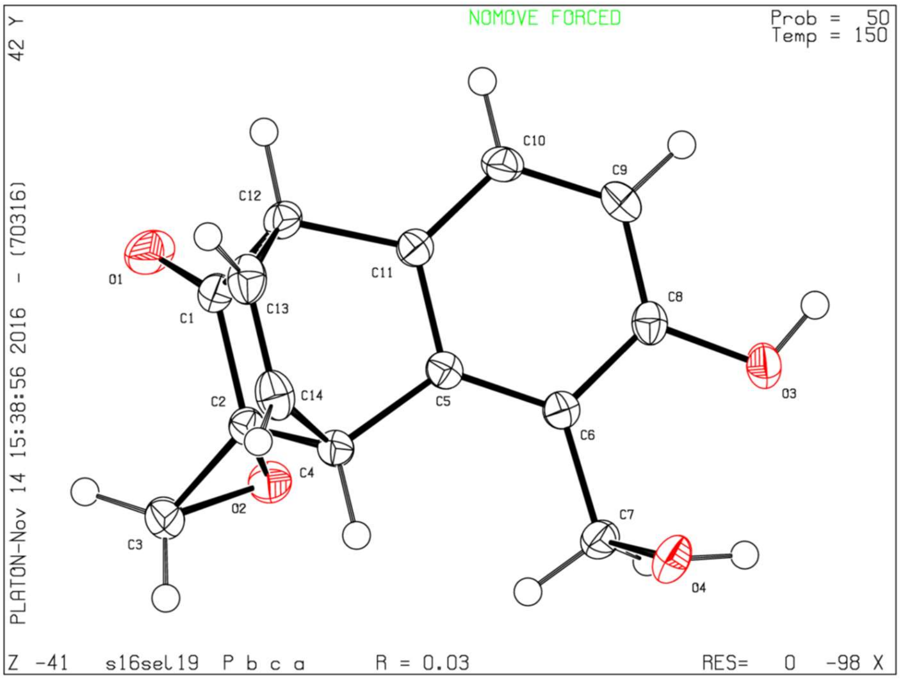

Table S9. Crystal data and structure refinement for (±)-**19**.

CCDC number	1952563	
Empirical formula	$C_{14}H_{12}O_4$	
Formula weight	244.24	
Temperature	150.00(10) K	
Wavelength	1.54184 Å	
Crystal system	Orthorhombic	
Space group	Pbca	
Unit cell dimensions	a = 9.91860(10) Å	α= 90°.
	b = 9.17150(10) Å	β= 90°.
	c = 25.0459(4) Å	γ = 90°.
Volume	2278.39(5) $Å^3$	
Z	8	
Density (calculated)	1.424 Mg/m^3	
Absorption coefficient	0.872 mm^{-1}	
F(000)	1024	
Crystal size	0.100 x 0.080 x 0.050 mm^3	
Theta range for data collection	3.529 to 73.167°.	
Index ranges	-11<=h<=12, -9<=k<=11, -31<=l<=30	
Reflections collected	15259	
Independent reflections	2263 [R(int) = 0.0319]	
Completeness to theta = 67.684°	100.0 %	
Absorption correction	Semi-empirical from equivalents	
Max. and min. transmission	1.00000 and 0.82400	
Refinement method	Full-matrix least-squares on F^2	
Data / restraints / parameters	2263 / 0 / 171	
Goodness-of-fit on F^2	1.069	
Final R indices [I>2sigma(I)]	R1 = 0.0347, wR2 = 0.0870	
R indices (all data)	R1 = 0.0379, wR2 = 0.0893	
Extinction coefficient	n/a	
Largest diff. peak and hole	0.265 and -0.197 e.$Å^{-3}$	

Table S10. Atomic coordinates (x 10^4) and equivalent isotropic displacement parameters (Å^{2}x 10^3) for (±)-**19**. U(eq) is defined as one third of the trace of the orthogonalized U^{ij} tensor.

	x	y	z	U(eq)
O(1)	2818(1)	6824(1)	2719(1)	32(1)
O(2)	2084(1)	7482(1)	3813(1)	23(1)
O(3)	6751(1)	5435(1)	4992(1)	24(1)
O(4)	6271(1)	8557(1)	5210(1)	23(1)
C(1)	3570(1)	7453(1)	3018(1)	20(1)
C(2)	3101(1)	8260(1)	3516(1)	18(1)
C(3)	1732(1)	8840(2)	3552(1)	24(1)
C(4)	4291(1)	8883(1)	3824(1)	19(1)
C(5)	5180(1)	7570(1)	3951(1)	17(1)
C(6)	5549(1)	7089(1)	4456(1)	17(1)
C(7)	5144(1)	7830(1)	4967(1)	21(1)
C(8)	6385(1)	5851(1)	4490(1)	19(1)
C(9)	6807(1)	5112(1)	4035(1)	22(1)
C(10)	6412(1)	5610(1)	3532(1)	21(1)
C(11)	5609(1)	6834(1)	3490(1)	18(1)
C(12)	5108(1)	7506(1)	2972(1)	20(1)
C(13)	5432(1)	9128(2)	2982(1)	24(1)
C(14)	5027(1)	9825(1)	3418(1)	24(1)

Table S11. Bond lengths [Å] for (±)-**19**.

O(1)-C(1)	1.2052(16)	C(5)-C(11)	1.4030(16)
O(2)-C(2)	1.4426(14)	C(6)-C(8)	1.4086(17)
O(2)-C(3)	1.4488(16)	C(6)-C(7)	1.5034(16)
O(3)-C(8)	1.3638(14)	C(7)-H(7A)	0.9900
O(3)-H(3)	0.88(2)	C(7)-H(7B)	0.9900
O(4)-C(7)	1.4370(15)	C(8)-C(9)	1.3895(17)
O(4)-H(4A)	0.83(2)	C(9)-C(10)	1.3966(17)
C(1)-C(2)	1.5225(16)	C(9)-H(9)	0.9500
C(1)-C(12)	1.5299(17)	C(10)-C(11)	1.3810(17)
C(2)-C(3)	1.4611(17)	C(10)-H(10)	0.9500
C(2)-C(4)	1.5208(16)	C(11)-C(12)	1.5219(16)
C(3)-H(3A)	0.9900	C(12)-C(13)	1.5218(18)
C(3)-H(3B)	0.9900	C(12)-H(12)	1.0000
C(4)-C(14)	1.5211(17)	C(13)-C(14)	1.3273(19)
C(4)-C(5)	1.5259(16)	C(13)-H(13)	0.9500
C(4)-H(4)	1.0000	C(14)-H(14)	0.950
C(5)-C(6)	1.3883(16)		

Table S12. Bond angles [°] for (±)-**19**.

C(2)-O(2)-C(3)	60.70(7)	O(4)-C(7)-C(6)	111.20(10)
C(8)-O(3)-H(3)	110.3(12)	O(4)-C(7)-H(7A)	109.4
C(7)-O(4)-H(4A)	109.2(13)	C(6)-C(7)-H(7A)	109.4
O(1)-C(1)-C(2)	123.59(11)	O(4)-C(7)-H(7B)	109.4
O(1)-C(1)-C(12)	125.76(11)	C(6)-C(7)-H(7B)	109.4
C(2)-C(1)-C(12)	110.60(10)	H(7A)-C(7)-H(7B)	108.0
O(2)-C(2)-C(3)	59.86(8)	O(3)-C(8)-C(9)	122.66(11)
O(2)-C(2)-C(4)	117.86(9)	O(3)-C(8)-C(6)	115.92(11)
C(3)-C(2)-C(4)	123.55(11)	C(9)-C(8)-C(6)	121.42(11)
O(2)-C(2)-C(1)	113.29(10)	C(8)-C(9)-C(10)	119.70(11)
C(3)-C(2)-C(1)	120.83(11)	C(8)-C(9)-H(9)	120.2
C(4)-C(2)-C(1)	111.10(10)	C(10)-C(9)-H(9)	120.2
O(2)-C(3)-C(2)	59.44(8)	C(11)-C(10)-C(9)	119.73(11)
O(2)-C(3)-H(3A)	117.8	C(11)-C(10)-H(10)	120.1
C(2)-C(3)-H(3A)	117.8	C(9)-C(10)-H(10)	120.1
O(2)-C(3)-H(3B)	117.8	C(10)-C(11)-C(5)	120.27(11)
C(2)-C(3)-H(3B)	117.8	C(10)-C(11)-C(12)	125.58(11)
H(3A)-C(3)-H(3B)	115.0	C(5)-C(11)-C(12)	114.14(10)
C(2)-C(4)-C(14)	104.30(9)	C(13)-C(12)-C(11)	108.17(10)
C(2)-C(4)-C(5)	104.97(9)	C(13)-C(12)-C(1)	103.94(10)
C(14)-C(4)-C(5)	108.14(10)	C(11)-C(12)-C(1)	104.30(9)
C(2)-C(4)-H(4)	112.9	C(13)-C(12)-H(12)	113.2
C(14)-C(4)-H(4)	112.9	C(11)-C(12)-H(12)	113.2
C(5)-C(4)-H(4)	112.9	C(1)-C(12)-H(12)	113.2
C(6)-C(5)-C(11)	121.07(11)	C(14)-C(13)-C(12)	114.89(11)
C(6)-C(5)-C(4)	126.47(10)	C(14)-C(13)-H(13)	122.6
C(11)-C(5)-C(4)	112.46(10)	C(12)-C(13)-H(13)	122.6
C(5)-C(6)-C(8)	117.81(11)	C(13)-C(14)-C(4)	114.91(11)
C(5)-C(6)-C(7)	124.08(11)	C(13)-C(14)-H(14)	122.5
C(8)-C(6)-C(7)	118.09(10)	C(4)-C(14)-H(14)	122.5

Table S13. Anisotropic displacement parameters (Å^{2}x 10^3) for (±)-**19**. The anisotropic displacement factor exponent takes the form: $-2\pi^2$[h^2 $a^{*2}U^{11}$ + ... + 2 h k a* b* U^{12}]

	U^{11}	U^{22}	U^{33}	U^{23}	U^{13}	U^{12}
O(1)	22(1)	49(1)	27(1)	-14(1)	-5(1)	1(1)
O(2)	20(1)	28(1)	21(1)	3(1)	4(1)	-1(1)
O(3)	24(1)	27(1)	21(1)	5(1)	-3(1)	5(1)
O(4)	32(1)	20(1)	17(1)	2(1)	-5(1)	-6(1)
C(1)	19(1)	24(1)	16(1)	1(1)	-1(1)	2(1)
C(2)	18(1)	20(1)	16(1)	2(1)	0(1)	1(1)
C(3)	20(1)	31(1)	23(1)	2(1)	0(1)	5(1)
C(4)	20(1)	19(1)	18(1)	-2(1)	-2(1)	3(1)
C(5)	14(1)	17(1)	19(1)	-1(1)	-1(1)	-2(1)
C(6)	14(1)	18(1)	18(1)	-1(1)	-1(1)	-2(1)
C(7)	20(1)	24(1)	18(1)	-2(1)	-1(1)	0(1)
C(8)	16(1)	21(1)	20(1)	4(1)	-2(1)	-2(1)
C(9)	17(1)	22(1)	28(1)	0(1)	0(1)	5(1)
C(10)	17(1)	24(1)	22(1)	-4(1)	2(1)	2(1)
C(11)	13(1)	22(1)	18(1)	-1(1)	0(1)	-2(1)
C(12)	17(1)	27(1)	16(1)	0(1)	1(1)	1(1)
C(13)	20(1)	29(1)	24(1)	9(1)	-1(1)	-2(1)
C(14)	22(1)	20(1)	30(1)	4(1)	-5(1)	-2(1)

Table S14. Hydrogen coordinates (x 10^4) and isotropic displacement parameters ($Å^2$x 10 ^{3}) for (±)-**19**.

	x	y	z	U(eq)
H(3)	7350(20)	4720(20)	4977(7)	37(5)
H(4A)	6612(19)	8010(20)	5439(8)	36(5)
H(3A)	1584	9707	3780	29
H(3B)	1158	8791	3230	29
H(4)	4012	9437	4149	23
H(7A)	4425	8550	4892	25
H(7B)	4777	7097	5218	25
H(9)	7361	4271	4067	27
H(10)	6696	5109	3220	25
H(12)	5458	7000	2646	24
H(13)	5891	9604	2698	29
H(14)	5184	10836	3472	29

Table S15. Torsion angles [°] for (±)-**19**.

C(3)-O(2)-C(2)-C(4)	114.58(12)	C(5)-C(6)-C(8)-O(3)	178.49(10)
C(3)-O(2)-C(2)-C(1)	-113.27(12)	C(7)-C(6)-C(8)-O(3)	0.15(16)
O(1)-C(1)-C(2)-O(2)	42.13(17)	C(5)-C(6)-C(8)-C(9)	-1.37(18)
C(12)-C(1)-C(2)-O(2)	-135.50(10)	C(7)-C(6)-C(8)-C(9)	-179.71(11)
O(1)-C(1)-C(2)-C(3)	-25.56(19)	O(3)-C(8)-C(9)-C(10)	-179.00(11)
C(12)-C(1)-C(2)-C(3)	156.82(11)	C(6)-C(8)-C(9)-C(10)	0.86(19)
O(1)-C(1)-C(2)-C(4)	177.49(12)	C(8)-C(9)-C(10)-C(11)	0.09(19)
C(12)-C(1)-C(2)-C(4)	-0.13(13)	C(9)-C(10)-C(11)-C(5)	-0.48(18)
C(4)-C(2)-C(3)-O(2)	-105.27(12)	C(9)-C(10)-C(11)-C(12)	179.68(11)
C(1)-C(2)-C(3)-O(2)	100.72(12)	C(6)-C(5)-C(11)-C(10)	-0.07(18)
O(2)-C(2)-C(4)-C(14)	-170.97(10)	C(4)-C(5)-C(11)-C(10)	-179.43(11)
C(3)-C(2)-C(4)-C(14)	-100.29(13)	C(6)-C(5)-C(11)-C(12)	179.79(10)
C(1)-C(2)-C(4)-C(14)	55.92(12)	C(4)-C(5)-C(11)-C(12)	0.43(14)
O(2)-C(2)-C(4)-C(5)	75.41(12)	C(10)-C(11)-C(12)-C(13)	-129.30(13)
C(3)-C(2)-C(4)-C(5)	146.08(11)	C(5)-C(11)-C(12)-C(13)	50.85(13)
C(1)-C(2)-C(4)-C(5)	-57.71(12)	C(10)-C(11)-C(12)-C(1)	120.50(13)
C(2)-C(4)-C(5)-C(6)	-120.40(12)	C(5)-C(11)-C(12)-C(1)	-59.35(13)
C(14)-C(4)-C(5)-C(6)	128.71(12)	O(1)-C(1)-C(12)-C(13)	126.52(14)
C(2)-C(4)-C(5)-C(11)	58.93(12)	C(2)-C(1)-C(12)-C(13)	-55.92(12)
C(14)-C(4)-C(5)-C(11)	-51.97(13)	O(1)-C(1)-C(12)-C(11)	-120.22(14)
C(11)-C(5)-C(6)-C(8)	0.97(17)	C(2)-C(1)-C(12)-C(11)	57.34(12)
C(4)-C(5)-C(6)-C(8)	-179.76(11)	C(11)-C(12)-C(13)-C(14)	-51.26(14)
C(11)-C(5)-C(6)-C(7)	179.20(11)	C(1)-C(12)-C(13)-C(14)	59.18(13)
C(4)-C(5)-C(6)-C(7)	-1.53(19)	C(12)-C(13)-C(14)-C(4)	-0.93(16)
C(5)-C(6)-C(7)-O(4)	-108.14(13)	C(2)-C(4)-C(14)-C(13)	-57.96(13)
C(8)-C(6)-C(7)-O(4)	70.09(14)	C(5)-C(4)-C(14)-C(13)	53.38(14)

Table S16. Hydrogen bonds for (±)-**19** [Å and °].

D-H...A	d(D-H)	d(H...A)	d(D...A)	<(DHA)
O(3)-H(3)...O(4)#1	0.88(2)	1.83(2)	2.6672(13)	157.1(17)
O(4)-H(4A)...O(2)#2	0.83(2)	1.98(2)	2.7476(13)	152.0(18)

Symmetry transformations used to generate equivalent atoms:
#1 -x+3/2,y-1/2,z #2 x+1/2,-y+3/2,-z+1

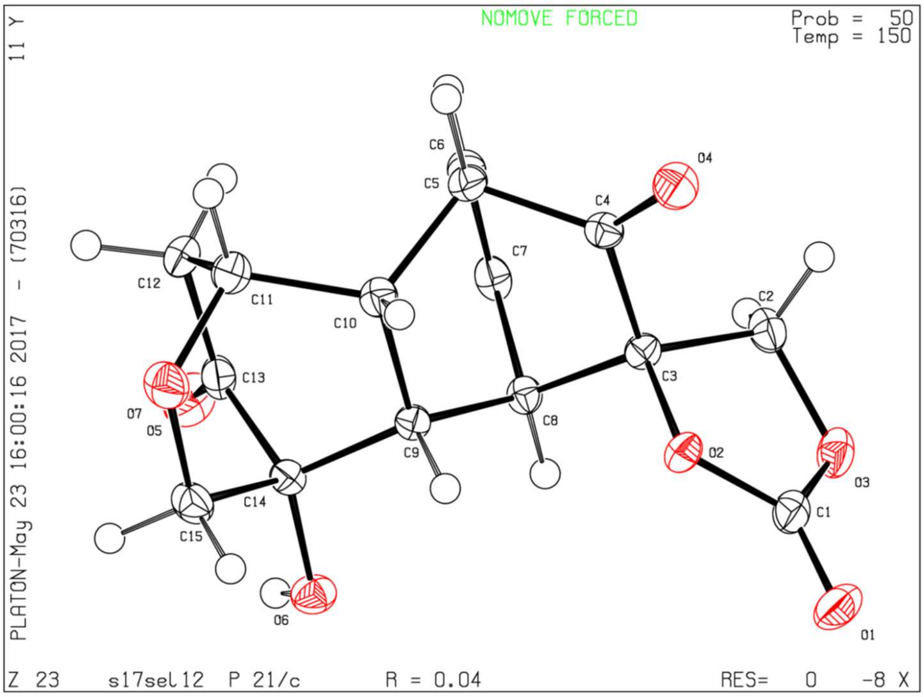

Table S17. Crystal data and structure refinement for (±)-**27**.

CCDC Number	1952564	
Empirical formula	$C_{15}H_{14}O_7$	
Formula weight	306.26	
Temperature	150.00(10) K	
Wavelength	1.54184 Å	
Crystal system	Monoclinic	
Space group	$P2_1/c$	
Unit cell dimensions	a = 11.0103(2) Å	α= 90°.
	b = 10.83020(10) Å	β= 103.155(2)°.
	c = 10.8212(2) Å	γ = 90°.
Volume	1256.50(4) $Å^3$	
Z	4	
Density (calculated)	1.619 Mg/m^3	
Absorption coefficient	1.110 mm^{-1}	
F(000)	640	
Crystal size	0.200 x 0.200 x 0.050 mm^3	
Theta range for data collection	4.123 to 73.151°.	
Index ranges	-13<=h<=13, -13<=k<=10, -12<=l<=13	
Reflections collected	14877	
Independent reflections	2509 [R(int) = 0.0305]	
Completeness to theta = 1.000°	0.0 %	
Absorption correction	Semi-empirical from equivalents	
Max. and min. transmission	1.00000 and 0.86486	
Refinement method	Full-matrix least-squares on F^2	
Data / restraints / parameters	2509 / 0 / 203	
Goodness-of-fit on F^2	1.111	
Final R indices [I>2sigma(I)]	R1 = 0.0386, wR2 = 0.1004	
R indices (all data)	R1 = 0.0407, wR2 = 0.1019	
Extinction coefficient	n/a	
Largest diff. peak and hole	0.302 and -0.244 e.$Å^{-3}$	

Table S18. Atomic coordinates (x 10^4) and equivalent isotropic displacement parameters ($Å^2$x 10^3) for (±)-**27**. U(eq) is defined as one third of the trace of the orthogonalized U^{ij} tensor.

	x	y	z	U(eq)
O(1)	7248(1)	4190(1)	2958(1)	28(1)
C(1)	6335(1)	4791(1)	2546(2)	19(1)
O(2)	5441(1)	4998(1)	3197(1)	17(1)
C(2)	4990(2)	6127(2)	1338(2)	19(1)
O(3)	6047(1)	5317(1)	1401(1)	22(1)
C(3)	4393(1)	5619(1)	2375(1)	14(1)
O(4)	4666(1)	7391(1)	3751(1)	24(1)
C(4)	3951(1)	6645(1)	3165(1)	16(1)
O(5)	45(1)	3301(1)	1122(1)	28(1)
C(5)	2566(1)	6543(1)	3125(2)	17(1)
O(6)	2268(1)	2212(1)	2195(1)	20(1)
C(6)	1937(1)	6454(2)	1730(2)	19(1)
O(7)	1300(1)	3860(1)	4784(1)	24(1)
C(7)	2294(1)	5516(1)	1105(1)	17(1)
C(8)	3305(1)	4718(1)	1895(1)	14(1)
C(9)	2902(1)	4223(1)	3080(1)	14(1)
C(10)	2468(1)	5300(1)	3825(1)	16(1)
C(11)	1177(2)	4990(2)	4052(2)	20(1)
C(12)	184(1)	4774(2)	2835(2)	21(1)
C(13)	632(1)	3748(2)	2105(1)	18(1)
C(14)	1882(1)	3229(1)	2807(1)	15(1)
C(15)	1668(2)	2828(1)	4114(2)	20(1)

Table S19. Bond lengths [Å] and angles [°] for (±)-**27**.

O(1)-C(1)	1.195(2)	C(5)-C(4)-C(3)	111.85(12)
C(1)-O(3)	1.334(2)	C(6)-C(5)-C(4)	105.11(12)
C(1)-O(2)	1.3534(18)	C(6)-C(5)-C(10)	111.25(12)
O(2)-C(3)	1.4517(17)	C(4)-C(5)-C(10)	103.22(12)
C(2)-O(3)	1.446(2)	C(6)-C(5)-H(5)	112.2
C(2)-C(3)	1.526(2)	C(4)-C(5)-H(5)	112.2
C(2)-H(2A)	0.9900	C(10)-C(5)-H(5)	112.2
C(2)-H(2B)	0.9900	C(14)-O(6)-H(1)	109.4(19)
C(3)-C(8)	1.541(2)	C(7)-C(6)-C(5)	115.82(14)
C(3)-C(4)	1.547(2)	C(7)-C(6)-H(6)	122.1
O(4)-C(4)	1.2026(19)	C(5)-C(6)-H(6)	122.1
C(4)-C(5)	1.520(2)	C(15)-O(7)-C(11)	112.58(11)
O(5)-C(13)	1.213(2)	C(6)-C(7)-C(8)	114.48(13)
C(5)-C(6)	1.515(2)	C(6)-C(7)-H(7)	122.8
C(5)-C(10)	1.561(2)	C(8)-C(7)-H(7)	122.8
C(5)-H(5)	1.0000	C(7)-C(8)-C(3)	104.15(12)
O(6)-C(14)	1.4007(18)	C(7)-C(8)-C(9)	110.76(12)
O(6)-H(1)	0.86(3)	C(3)-C(8)-C(9)	106.75(11)
C(6)-C(7)	1.328(2)	C(7)-C(8)-H(8)	111.6
C(6)-H(6)	0.9500	C(3)-C(8)-H(8)	111.6
O(7)-C(15)	1.4389(19)	C(9)-C(8)-H(8)	111.6
O(7)-C(11)	1.4472(19)	C(14)-C(9)-C(8)	114.83(12)
C(7)-C(8)	1.512(2)	C(14)-C(9)-C(10)	109.13(11)
C(7)-H(7)	0.9500	C(8)-C(9)-C(10)	110.52(12)
C(8)-C(9)	1.5446(19)	C(14)-C(9)-H(9)	107.3
C(8)-H(8)	1.0000	C(8)-C(9)-H(9)	107.3
C(9)-C(14)	1.5353(19)	C(10)-C(9)-H(9)	107.3
C(9)-C(10)	1.5537(19)	C(11)-C(10)-C(9)	108.98(12)
C(9)-H(9)	1.0000	C(11)-C(10)-C(5)	115.79(13)
C(10)-C(11)	1.534(2)	C(9)-C(10)-C(5)	109.42(11)
C(10)-H(10)	1.0000	C(11)-C(10)-H(10)	107.4
C(11)-C(12)	1.526(2)	C(9)-C(10)-H(10)	107.4
C(11)-H(11)	1.0000	C(5)-C(10)-H(10)	107.4
C(12)-C(13)	1.509(2)	O(7)-C(11)-C(12)	107.55(13)
C(12)-H(12A)	0.9900	O(7)-C(11)-C(10)	107.28(12)
C(12)-H(12B)	0.9900	C(12)-C(11)-C(10)	113.72(13)
C(13)-C(14)	1.520(2)	O(7)-C(11)-H(11)	109.4
C(14)-C(15)	1.547(2)	C(12)-C(11)-H(11)	109.4
C(15)-H(15A)	0.9900	C(10)-C(11)-H(11)	109.4
C(15)-H(15B)	0.9900	C(13)-C(12)-C(11)	108.06(12)
		C(13)-C(12)-H(12A)	110.1
O(1)-C(1)-O(3)	125.56(15)	C(11)-C(12)-H(12A)	110.1
O(1)-C(1)-O(2)	123.01(15)	C(13)-C(12)-H(12B)	110.1
O(3)-C(1)-O(2)	111.42(13)	C(11)-C(12)-H(12B)	110.1
C(1)-O(2)-C(3)	109.20(11)	H(12A)-C(12)-H(12B)	108.4
O(3)-C(2)-C(3)	102.93(12)	O(5)-C(13)-C(12)	125.48(14)
O(3)-C(2)-H(2A)	111.2	O(5)-C(13)-C(14)	122.13(14)
C(3)-C(2)-H(2A)	111.2	C(12)-C(13)-C(14)	112.15(13)
O(3)-C(2)-H(2B)	111.2	O(6)-C(14)-C(13)	113.07(12)
C(3)-C(2)-H(2B)	111.2	O(6)-C(14)-C(9)	110.77(12)
H(2A)-C(2)-H(2B)	109.1	C(13)-C(14)-C(9)	112.09(12)
C(1)-O(3)-C(2)	108.73(11)	O(6)-C(14)-C(15)	109.73(12)
O(2)-C(3)-C(2)	101.62(11)	C(13)-C(14)-C(15)	104.60(12)
O(2)-C(3)-C(8)	111.23(11)	C(9)-C(14)-C(15)	106.15(12)
C(2)-C(3)-C(8)	114.38(12)	O(7)-C(15)-C(14)	111.17(12)
O(2)-C(3)-C(4)	107.47(11)	O(7)-C(15)-H(15A)	109.4
C(2)-C(3)-C(4)	112.93(12)	C(14)-C(15)-H(15A)	109.4
C(8)-C(3)-C(4)	108.85(11)	O(7)-C(15)-H(15B)	109.4
O(4)-C(4)-C(5)	126.56(14)	C(14)-C(15)-H(15B)	109.4
O(4)-C(4)-C(3)	121.56(13)	H(15A)-C(15)-H(15B)	108.0

Table S20. Anisotropic displacement parameters ($Å^2$x 10^3) for (±)-**27**. The anisotropic displacement factor exponent takes the form: $-2\pi^2[h^2 a^{*2}U^{11} + ... + 2 h k a^* b^* U^{12}]$

	U^{11}	U^{22}	U^{33}	U^{23}	U^{13}	U^{12}
O(1)	16(1)	27(1)	40(1)	-9(1)	5(1)	3(1)
C(1)	15(1)	18(1)	25(1)	-9(1)	6(1)	-4(1)
O(2)	12(1)	21(1)	17(1)	0(1)	3(1)	2(1)
C(2)	21(1)	19(1)	19(1)	-1(1)	8(1)	-4(1)
O(3)	20(1)	26(1)	22(1)	-7(1)	11(1)	-5(1)
C(3)	14(1)	15(1)	13(1)	0(1)	2(1)	1(1)
O(4)	22(1)	22(1)	28(1)	-9(1)	5(1)	-4(1)
C(4)	18(1)	14(1)	15(1)	1(1)	4(1)	1(1)
O(5)	21(1)	34(1)	26(1)	-4(1)	-2(1)	0(1)
C(5)	16(1)	15(1)	21(1)	-2(1)	6(1)	1(1)
O(6)	20(1)	17(1)	21(1)	-4(1)	3(1)	1(1)
C(6)	15(1)	19(1)	23(1)	6(1)	2(1)	1(1)
O(7)	31(1)	23(1)	21(1)	2(1)	13(1)	0(1)
C(7)	16(1)	20(1)	15(1)	3(1)	1(1)	-4(1)
C(8)	15(1)	15(1)	13(1)	-1(1)	4(1)	-1(1)
C(9)	14(1)	14(1)	13(1)	1(1)	2(1)	0(1)
C(10)	17(1)	17(1)	14(1)	-1(1)	5(1)	0(1)
C(11)	22(1)	20(1)	22(1)	1(1)	10(1)	1(1)
C(12)	14(1)	21(1)	27(1)	2(1)	8(1)	2(1)
C(13)	15(1)	20(1)	20(1)	3(1)	4(1)	-3(1)
C(14)	16(1)	13(1)	16(1)	1(1)	5(1)	0(1)
C(15)	25(1)	17(1)	19(1)	3(1)	7(1)	-1(1)

Table S21. Hydrogen coordinates (x 10^4) and isotropic displacement parameters ($Å^2$x 10^3) for (±)-**27**.

	x	y	z	U(eq)
H(1)	1670(30)	1990(30)	1570(30)	55(8)
H(2A)	4406	6083	496	23
H(2B)	5262	6993	1515	23
H(5)	2253	7259	3548	21
H(6)	1326	7033	1326	23
H(7)	1942	5360	233	21
H(8)	3551	4033	1382	17
H(9)	3654	3844	3647	16
H(10)	3062	5345	4673	19
H(11)	900	5671	4549	24
H(12A)	-616	4540	3041	25
H(12B)	52	5537	2318	25
H(15A)	1011	2186	3991	24
H(15B)	2446	2465	4627	24

Table S22. Torsion angles [°] for (±)-**27**.

O(1)-C(1)-O(2)-C(3)	-172.66(14)	C(3)-C(8)-C(9)-C(10)	60.33(14)
O(3)-C(1)-O(2)-C(3)	6.03(16)	C(14)-C(9)-C(10)-C(11)	0.91(16)
O(1)-C(1)-O(3)-C(2)	-170.54(15)	C(8)-C(9)-C(10)-C(11)	128.11(13)
O(2)-C(1)-O(3)-C(2)	10.81(16)	C(14)-C(9)-C(10)-C(5)	-126.61(13)
C(3)-C(2)-O(3)-C(1)	-21.88(15)	C(8)-C(9)-C(10)-C(5)	0.58(16)
C(1)-O(2)-C(3)-C(2)	-18.84(14)	C(6)-C(5)-C(10)-C(11)	-73.28(16)
C(1)-O(2)-C(3)-C(8)	103.30(13)	C(4)-C(5)-C(10)-C(11)	174.47(12)
C(1)-O(2)-C(3)-C(4)	-137.64(12)	C(6)-C(5)-C(10)-C(9)	50.32(16)
O(3)-C(2)-C(3)-O(2)	23.82(14)	C(4)-C(5)-C(10)-C(9)	-61.93(14)
O(3)-C(2)-C(3)-C(8)	-96.13(14)	C(15)-O(7)-C(11)-C(12)	59.88(16)
O(3)-C(2)-C(3)-C(4)	138.64(12)	C(15)-O(7)-C(11)-C(10)	-62.82(16)
O(2)-C(3)-C(4)-O(4)	51.78(18)	C(9)-C(10)-C(11)-O(7)	60.13(15)
C(2)-C(3)-C(4)-O(4)	-59.47(19)	C(5)-C(10)-C(11)-O(7)	-176.04(12)
C(8)-C(3)-C(4)-O(4)	172.36(14)	C(9)-C(10)-C(11)-C(12)	-58.66(16)
O(2)-C(3)-C(4)-C(5)	-126.35(12)	C(5)-C(10)-C(11)-C(12)	65.17(17)
C(2)-C(3)-C(4)-C(5)	122.40(13)	O(7)-C(11)-C(12)-C(13)	-61.57(15)
C(8)-C(3)-C(4)-C(5)	-5.77(16)	C(10)-C(11)-C(12)-C(13)	57.08(17)
O(4)-C(4)-C(5)-C(6)	131.00(16)	C(11)-C(12)-C(13)-O(5)	176.32(15)
C(3)-C(4)-C(5)-C(6)	-50.99(15)	C(11)-C(12)-C(13)-C(14)	1.83(17)
O(4)-C(4)-C(5)-C(10)	-112.31(17)	O(5)-C(13)-C(14)-O(6)	0.6(2)
C(3)-C(4)-C(5)-C(10)	65.70(14)	C(12)-C(13)-C(14)-O(6)	175.27(12)
C(4)-C(5)-C(6)-C(7)	58.46(17)	O(5)-C(13)-C(14)-C(9)	126.64(15)
C(10)-C(5)-C(6)-C(7)	-52.58(18)	C(12)-C(13)-C(14)-C(9)	-58.66(16)
C(5)-C(6)-C(7)-C(8)	-1.89(19)	O(5)-C(13)-C(14)-C(15)	-118.79(16)
C(6)-C(7)-C(8)-C(3)	-58.58(16)	C(12)-C(13)-C(14)-C(15)	55.92(15)
C(6)-C(7)-C(8)-C(9)	55.84(17)	C(8)-C(9)-C(14)-O(6)	58.44(16)
O(2)-C(3)-C(8)-C(7)	178.06(11)	C(10)-C(9)-C(14)-O(6)	-176.85(11)
C(2)-C(3)-C(8)-C(7)	-67.53(15)	C(8)-C(9)-C(14)-C(13)	-68.88(16)
C(4)-C(3)-C(8)-C(7)	59.83(14)	C(10)-C(9)-C(14)-C(13)	55.84(15)
O(2)-C(3)-C(8)-C(9)	60.83(14)	C(8)-C(9)-C(14)-C(15)	177.50(12)
C(2)-C(3)-C(8)-C(9)	175.24(12)	C(10)-C(9)-C(14)-C(15)	-57.79(15)
C(4)-C(3)-C(8)-C(9)	-57.40(14)	C(11)-O(7)-C(15)-C(14)	2.25(17)
C(7)-C(8)-C(9)-C(14)	71.54(16)	O(6)-C(14)-C(15)-O(7)	178.54(12)
C(3)-C(8)-C(9)-C(14)	-175.69(12)	C(13)-C(14)-C(15)-O(7)	-59.88(15)
C(7)-C(8)-C(9)-C(10)	-52.44(15)	C(9)-C(14)-C(15)-O(7)	58.81(16)

Table S23. Hydrogen bonds for (±)-**27** [Å and °].

D-H...A	d(D-H)	d(H...A)	d(D...A)	<(DHA)
O(6)-H(1)...O(5)	0.86(3)	2.25(3)	2.7264(17)	115(2)
